# Nonamer dependent RAG cleavage at CpGs can explain mechanism of chromosomal translocations associated to lymphoid cancers

**DOI:** 10.1371/journal.pgen.1010421

**Published:** 2022-10-13

**Authors:** Amita M. Paranjape, Sagar S. Desai, Mayilaadumveettil Nishana, Urbi Roy, Namrata M. Nilavar, Amrita Mondal, Rupa Kumari, Gudapureddy Radha, Vijeth Kumar Katapadi, Bibha Choudhary, Sathees C. Raghavan

**Affiliations:** 1 Department of Biochemistry, Indian Institute of Science, Bangalore, India; 2 Institute of Bioinformatics and Applied Biotechnology, Electronics City, Bangalore, India; 3 Manipal Academy of Higher Education, Manipal, Karnataka, India; 4 Indian Institute of Science Education and Research, Thiruvananthapuram, Kerala, India; UT Health San Antonio: The University of Texas Health Science Center at San Antonio, UNITED STATES

## Abstract

Chromosomal translocations are considered as one of the major causes of lymphoid cancers. RAG complex, which is responsible for V(D)J recombination, can also cleave non-B DNA structures and cryptic RSSs in the genome leading to chromosomal translocations. The mechanism and factors regulating the illegitimate function of RAGs resulting in oncogenesis are largely unknown. Upon *in silico* analysis of 3760 chromosomal translocations from lymphoid cancer patients, we find that 93% of the translocation breakpoints possess adjacent cryptic nonamers (RAG binding sequences), of which 77% had CpGs in proximity. As a proof of principle, we show that RAGs can efficiently bind to cryptic nonamers present at multiple fragile regions and cleave at adjacent mismatches generated to mimic the deamination of CpGs. ChIP studies reveal that RAGs can indeed recognize these fragile sites on a chromatin context inside the cell. Finally, we show that AID, the cytidine deaminase, plays a significant role during the generation of mismatches at CpGs and reconstitute the process of RAG-dependent generation of DNA breaks both *in vitro* and inside the cells. Thus, we propose a novel mechanism for generation of chromosomal translocation, where RAGs bind to the cryptic nonamer sequences and direct cleavage at adjacent mismatch generated due to deamination of ^me^CpGs or cytosines.

## Author summary

Chromosomal translocations are hallmark of lymphoid cancers. Recombination activating genes (RAGs) and activation induced cytidine deaminase (AID) are known to be responsible for chromosomal translocations. RAGs bind to DNA sequence called recombination signal sequence (RSS) consisting of heptamer, nonamer and spacer, and are involved in antigen receptor rearrangements. RAGs can also recognize and cleave other non-B DNA structures, thus causing DNA breaks. CpG dinucleotides are frequent in mammalian genome, which upon AID mediated deamination may form single nucleotide mismatches. In the present study, based on analysis of over 3500 chromosomal translocations seen in lymphoid cancer patients, we report that majority of breakpoints are seen when distance between CpG and nonamer like sequences (cryptic nonamer) is less than 80 bp. Using various biochemical and cellular assays, we show that mismatches generated due to deamination at CpGs or cytosines get cleaved by RAGs if cryptic nonamer is present in proximity. We propose a novel mechanism by which both AID and RAGs act together leading to generation of chromosomal translocation.

## Introduction

Chromosomal abnormalities play a central role in oncogenesis. Among different abnormalities associated with lymphoid cancers, chromosomal translocations are widely studied [1,2] and are considered as a hallmark of hematopoietic neoplasms. Translocations are also seen in nonlymphoid cancers, particularly in sarcomas and epithelial tumors [2,3].

Immunoglobulin genes serve as one of the partners in several reciprocal chromosomal translocations characteristic of B-cell malignancies, like follicular lymphoma, acute lymphoblastic leukemia, and chronic lymphocytic leukemia [4,5]. Similarly, T lymphocyte malignancies are associated with translocations involving T cell receptors [6]. As a consequence of the translocation, transcriptional deregulation of the partner genes occurs, leading to growth advantage which may result in malignancies [7].

RAG (Recombination activating gene) complex, comprising RAG1 and RAG2, is known to be regulated by transcription factors, epigenetic regulators and microRNAs [8–10]. Deregulation of RAGs is known to play a major role in the generation of translocations in lymphoid tissues [4,5,11–13]. In the normal physiological scenario, RAGs are involved in generation of antigen receptor diversity. RAGs bind to the nonamer (ACAAAAACC) of the recombination signal sequence (RSS) and generate a nick at the 5’ end of the heptamer (CACAGTG). This in turn is followed by recombination events that lead to antibody diversity [10,14–18]. Reports indicate that only first 3 nucleotides of heptamer and 5^th^ and 6^th^ position of nonamer show 100% conservation in RSS and are crucial for V(D)J recombination [19–22]. RSS with sequences which deviate from consensus sequence is called as cryptic RSS (cRSS), and individually referred to as cryptic heptamer and cryptic nonamer. A study reported that the binding and cleavage efficiency of RAGs at cryptic RSS is 30 to 20,000 folds less than consensus RSSs [23–25]. Such sequences result in reduced recombination efficiency. It is estimated that ~10 million cRSSs are present in the human genome that support V(D)J recombination to ≥1% of that of the canonical RSS [26].

RAGs generate translocations via inter-chromosomal V(D)J recombination involving a set of compatible RSSs located on two different chromosomes [2,27,28]. Illegitimate RAG cleavage at cryptic RSS, present outside the antigen receptor loci, has been a well-established cause of chromosomal translocations [24–26, 29]. Certain “hotspots” or fragile regions on the DNA are more prone to breakage and can lead to translocations. Since convincing RSS motifs are seldom found near patient breakpoints, it is unlikely that the sole process involved is RSS-dependent DNA cleavage. Several models have been proposed to explain the fragility at non-antigen receptor loci.

One of the most plausible scenarios is RAGs acting as a structure-specific endonuclease, recognizing non-B DNA structures and nicking DNA at sites of double-stranded to single-stranded DNA transition, following which the nicks are converted to double-strand breaks (DSBs) by several mechanisms [12,28,30–34]. Studies on structure-specific nuclease activity of RAGs have revealed the importance of sequence in determining the affinity of RAGs to non-B DNA [35–37]. It was reported that nonamers adjacent to a non-B DNA could enhance RAG-mediated cleavage [38]. However, relevance of this observation was unclear within cells.

CpG islands are stretches of DNA sequence with high number of CpG dinucleotides usually located in the vicinity of coding regions in the genome. In mammals, cytosine in CpG di-nucleotides is predominantly methylated [39]. Activation induced cytidine deaminase (AID), primarily expressed in germinal B cells, is responsible for somatic hypermutation and class switch recombination [40]. Cytosine can get converted to uracil via oxidative deamination by AID, whereas 5^m^C gets deaminated to thymine [41,42]. Constitutive AID activation in malignant cells further raises potential for inducing mismatches in the genome [43,44]. Thus, AID activity at CpG islands is speculated to have a role in various chromosomal translocations [45–47].

Previous *in silico* studies have revealed the presence of CpGs adjacent to many chromosomal translocation junctions, including *BCL2* MBR in follicular lymphoma and *BCL1* in mantle cell lymphoma [48,49]. It was hypothesized that mismatches generated at CpG sites could make those chromosomal regions fragile. We have previously reported that RAGs can cleave at a single nucleotide mismatch, when present adjacent to a nonamer [38]. Therefore, we sought to investigate how often such canonical/cryptic nonamers and CpGs are present near translocation breakpoints. We also investigated whether a combination of RAG and AID activities can explain mechanism of chromosomal fragility in regions associated with several leukemias and lymphomas.

In the present study, a comprehensive search for the presence of cryptic nonamers associated with CpGs proximal to chromosomal translocation breakpoints associated with lymphoid cancer patients, revealed the frequency of occurrence of cryptic nonamers near CpG is significantly high compared to random occurrence of such sequences elsewhere in the genome. Using biochemical assays, we establish that these breakpoints can indeed be recognized and cleaved by RAGs. Further, we show that nonamer binding domain (NBD), of RAG1 is responsible for binding to cryptic nonamers present at translocation breakpoint regions. RAG1 ChIP assays demonstrate RAG1 binding to various fragile regions within lymphoid cells. Further we show that AID plays a major role in the generation of mismatches in CpGs, which were recognized and cleaved by RAGs. Thus, we propose a novel mechanism for the generation of lymphoid-specific chromosomal translocations, where RAGs bind to a cryptic nonamer and cleave at an adjacent mismatch generated due to deamination of methylated CpG sites.

## Results

Pathologic rearrangements of chromosomes through DNA translocations have been reported in the majority of lymphoid malignancies [2,50]. Previously, it was shown that nonamer of RSS or even a cryptic nonamer when present adjacent to a non-B DNA can enhance the efficiency of RAG cleavage significantly [38]. However, the physiological relevance of this remained unexplored. Another study had shown a remarkable correlation between occurrence of chromosomal translocations and CpGs [48]. Owing to this, we investigated the mechanism of chromosomal fragility in the context of CpGs and nonamers in lymphoid cancer.

### Cryptic nonamers and CpGs are present adjacent to chromosomal translocation breakpoints in patients with lymphoid malignancy

**Fig 1:**
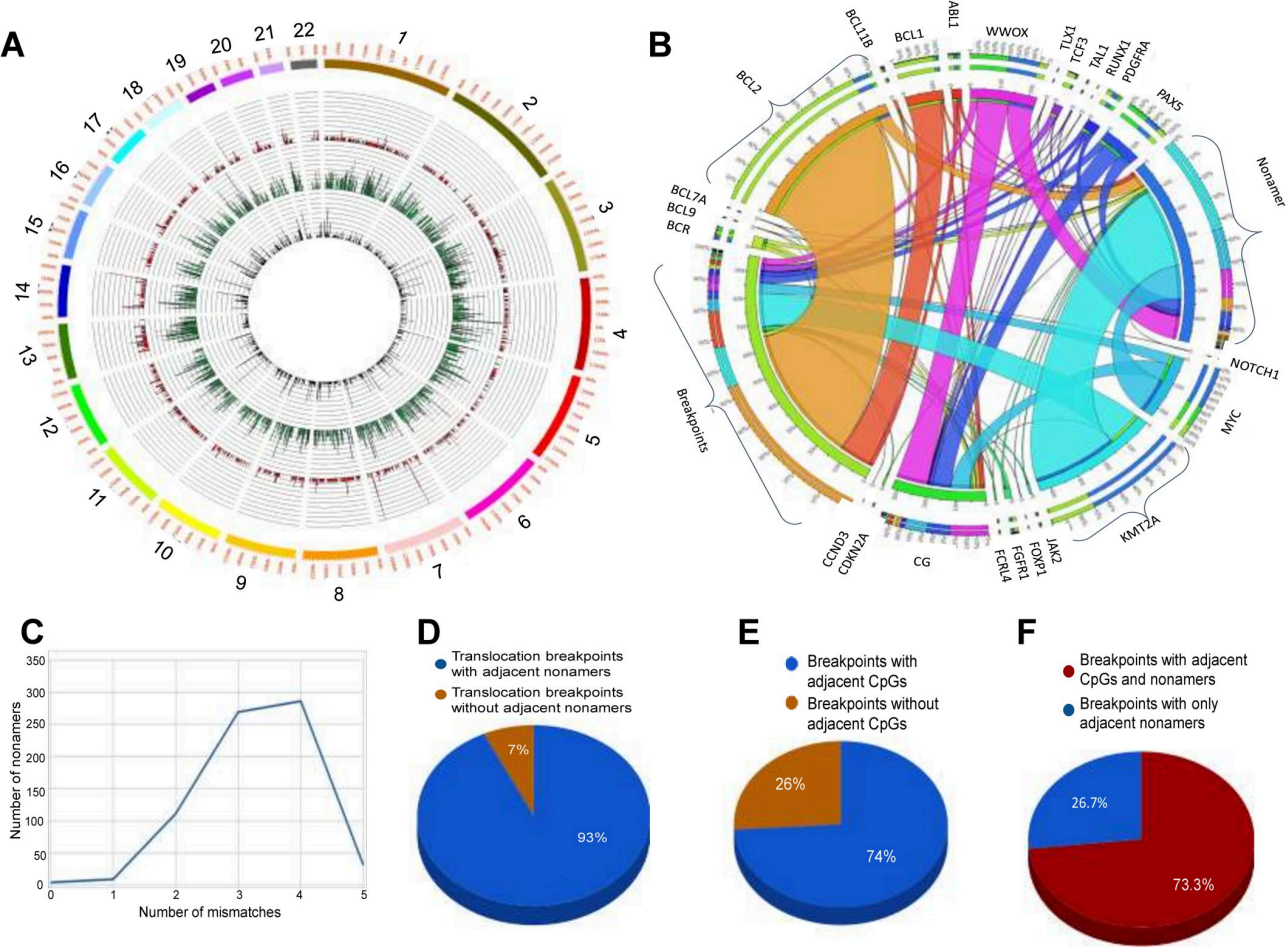
Chromosomal translocation breakpoints in patients with lymphoid malignancies and frequency of occurrence of CpGs and cryptic nonamer at or near breakpoint regions. **A**. Circos plot comparing the number of chromosomal translocation breakpoints, cryptic nonamers and CpGs across the chromosomes in a window of 1 Kb. The inner most track displays the number of CpGs, middle one represents number of cryptic nonamers and the outermost track is for number of translocation breakpoint pairs from patients (n = 3760). **B**. Circos plot shows genes having fragile sites and their significant association with adjacent CpG and cryptic nonamer sequences. Individual gene on the Circos plot connects the breakpoints to corresponding cryptic nonamers and CG sites present within 80 base pairs from the breakpoints. **C.** A line plot representing distribution of cryptic nonamers when mismatches allowed are 1–5 nt at the vicinity of chromosomal translocation breakpoints. **D**. Pie chart depicting number of patients (in %) with cryptic nonamers adjacent to known chromosomal translocation breakpoint pairs in lymphoid patients analyzed (n = 3760). **E.** Pie chart showing number of patients (in %) having CpG site/s adjacent to translocation breakpoints analyzed in panel B. **F.** Pie chart showing number of patients (in %) having both CpG site/s and adjacent cryptic nonamer/s at or near translocation points analyzed in panel B.

We analyzed databases, TICdb (Translocation breakpoints In Cancer database) [51,52], COSMIC (Catalogue Of Somatic Mutations In Cancer) (http://www.sanger.ac.uk/genetics/CGP/cosmic/) [51,52] and published literature for the genes from the most common chromosomal rearrangements in human lymphoid malignancies [48,53,54]. Genomic data analysis of 3760 chromosomal translocation breakpoints associated with lymphoid malignancies and 1420 genes harboring these breakpoints, revealed clustering of patient breakpoints around CpG sites and cryptic nonamers (Fig 1A and 1B). Examples of genes involved in these recurrent translocations include *ABL1*, *BCL1*, *BCL2*, *BCL9*, *BCL11B*, *BCL7A*, *BCR*, *E2A*, *MYC*, *FGFR1*, *KMT2A*, *CCND1*, *CCND3*, *FOXP1*, *LCK* and *SIL* (Figs 1B, S1 and S1 Table).

The translocation breakpoints junctions retrieved from TICdb [51,52], COSMIC [51,52] and published literature [48,53,54] were mapped on to hg19 and the regions with breaks were examined for the presence of cryptic nonamers and CpGs within a distance of 100 bp from the breakpoints. Our results revealed that breakpoints are clustered around cryptic nonamers in several translocations including those seen in malignancies like mantle cell lymphoma, B/T-cell acute lymphoblastic leukemia, mucosa-associated lymphoid tissue lymphoma, chronic myelogenous leukemia, follicular lymphoma, *etc* (S1 Table). Cryptic nonamers with 2, 3, 4 or 5 mismatches in the sequence were observed adjacent to the breakpoint peaks and the ones with 3 and 4 mismatches were most abundant in number (Fig 1C). Therefore, nonamers with upto 4 nt variation were considered as a cryptic nonamer in our study and used for further investigation. Interestingly, 93% of the translocations in lymphoid cancers have breakpoints adjacent to cryptic nonamers, of which 73% had CpGs in close vicinity to breakpoints (Fig 1D–1F).

Considering that enrichment of CpGs have been seen in several gene promoters, we examined how often chromosomal translocation breakpoints are observed in promoters. Analysis showed that out of all the breakpoints only 2% (74) were distributed in promoter regions, in which just 36 breaks (~49%) have CpG-cryptic nonamer clustering in their vicinity (S2A Fig). This suggests that although promoters are enriched with CpGs, majority of the breakpoints seen in the patients occurred elsewhere in the gene.

To analyze if there was any correlation among the breakpoints, nonamers and CpG at the genomic level, regions with the unique break points were binned separate from the genomic regions without breaks. Interestingly, the genomic regions with the breaks were nonrandom and clustered majorly on specific regions of chromosomes 14, 7, 9, 8, 18 etc. (Fig 1A), and the control regions were from the entire genome not harboring any breaks. We began the analysis by computing breaks/ Mb of the genome. We got a direct correlation of the frequency of breaks to the size of the genomic region (1.6 breaks /Mb of genome, correlation value r = 0.6). Following which, we performed similar analysis on regions of the entire genome split into windows of 1 kb each. On comparing the frequency between regions with and without breaks, we found that the number of cryptic nonamers were significantly higher (chi-square statistic 57.98, p-value<0.00001) in the windows having breaks with a median of 73 cryptic nonamers/kb. In contrast, in regions with no breaks a median of 40 cryptic nonamers/kb was observed (Mann Whitney coefficient W = 3302, p-value = 5.54e-05) (S2B Fig) Further analysis was done in windows of 100 bp across the genome. There were 30 million windows, which were segregated into bins with unique breaks and without breaks (control). Results revealed that there were only 55 bins with atleast 3 unique breaks, and all the other regions were controls.

Further, we investigated the occurrence of cryptic nonamers or CpGs within a window of 100 bp from the breakpoint and observed that 93% of the translocations in lymphoid cancers have breakpoints adjacent to cryptic nonamers, out of which 73% had CpGs in close vicinity to breakpoints as described above (Fig 1D–1F).

**Fig 2:**
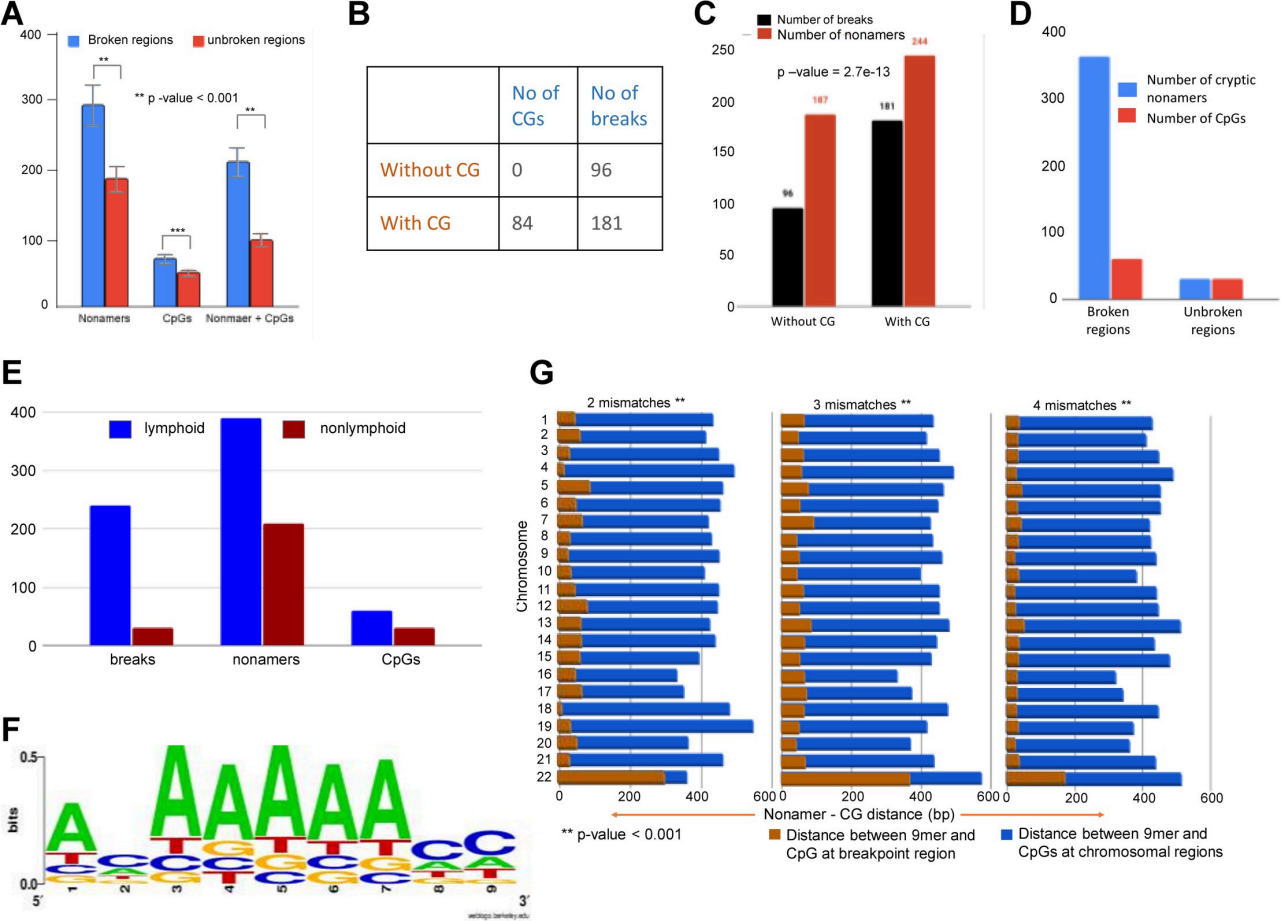
Evaluation of occurrence of cryptic nonamer and CpGs in human genome and their correlation with incidence of patient breakpoints. **A.** A histogram depicting the number of cryptic nonamers or CpGs or CpGs and cryptic nonamers in broken (blue) and unbroken (red) windows within genic regions. In the case of unbroken region, an equivalent number of random regions with similar GC contents were considered. The difference in numbers was found to be significant as per t-test (p-value < 0.001). **B, C.** Table and graph showing the number of CpGs, cryptic nonamers and translocation breakpoints, when a length of 100 bp was considered from the breakpoint region. **D.** Bar diagram showing the occurrence of CpGs and cryptic nonamers around breakpoints within a distance of 100 base pairs compared to random, unbroken regions. **E.** A bar graph comparing the number of breaks, cryptic nonamers, and CpGs seen between regions in lymphoid and nonlymphoid cancers. It is seen that all the three numbers are higher in the case of lymphoid breaks. **F.** Position weight matrix logo showing conservation in nucleotide positions in various translocation breakpoint junctions. **G.** Bar graphs showing the distance between CpG and cryptic nonamers next to patient break points (brown) as compared to the same at random (blue) regions as per chi-square test (p-value < 0.001).

For further study, we focused on only those windows where the number of breaks was at least 3 per 100 bases. We performed a comparative analysis between 100 base pair windows with break clusters (3 breaks or more) and windows without breaks in gene bodies. 39 broken windows from genic regions and same number of unbroken windows with comparable GC content out of a total of 24293 unbroken regions from genic regions were considered for the study. Results showed that 39 broken windows had ~8 cryptic nonamers (300 total), 1.8 CpGs (71 total), and a total of 215 cryptic nonamers had accompanying CpGs (Fig 2A). In contrast no-break region had only 190 cryptic nonamers, 50 CpGs, and only 99 cryptic nonamers had adjacent CpGs (t = 8.10931, p-value< 0.000629) (Fig 2A). Besides, out of a total of 39 windows with break clusters within gene bodies, 74% contained CpGs, and 69% contained both cryptic nonamers and CpGs, while out of 39 no-break windows, only 58% contained CpGs, and 53% contained both cryptic nonamers and CpGs (S2C Fig). Since there were 24293 no break windows, and we could select only one set of 39 windows in the previous case, we performed the similar analysis on 1000 iterations of random 39 windows with the same AT-GC content. Interestingly, results obtained were comparable in all the cases and ranged between 56–58% windows containing CpGs while 52–54% had both cryptic nonamer and CpGs (S2C Fig).

Interestingly, when the break windows were analyzed irrespective of genic regions, we observed that the number of breaks was significantly higher (chi-square statistic = 37.9 and p-value = 7.4e-10) when CpGs were present (181 breaks) as compared to regions with no CpGs (96 breaks) (Fig 2B). When a comparison was made with presence of nonamer (244) and CpG (84), to cryptic nonamer (187) and no CpG (0) in the vicinity of break, we obtained a chi-square statistic = 57.84 and p-value = 2.7e-13) (Fig 2B and 2C); indicating contribution of cryptic nonamers and CpGs to the occurrence of breaks (Fig 2C).

Further, we analyzed the number of nonamers and CpGs in windows of 100 bp with and without breaks. Results revealed 55 windows of 100 bp each where atleast 3 breaks were present. We checked for the presence of CpG and nonamers in the 55 windows with breaks. Similarly, windows of 100 bp without breaks were selected and checked for CpGs and nonamers, and chi-square test was performed. Interestingly, we observed that the 100 bp windows had an average of 13 cryptic nonamers, 2 CpGs and 8 breaks, while it was only 1 cryptic nonamer and 1 CpG when there was no break (chi square statistic = 94.43 and p-value = 3.1 e-21), indicating a greater number of CpGs and cryptic nonamers near breakpoints, accounting for a total of 390 cryptic nonamers and 60 CpGs in 30 windows (Fig 2D). In contrast, unbroken regions of the genome have fewer number of CpGs and cryptic nonamers present in proximity (Fig 2D).

To address whether the region with breaks (all breakpoints) and no-breaks were equally distributed in euchromatin and heterochromatin, we intersected the break and no-break regions with euchromatin and heterochromatin regions of the genome. Heterochromatin and euchromatin regions of the genome were extracted from the Giemsa staining information of the chromosomes using the Table Browser utility of the UCSC Genome Browser (https://genome.ucsc.edu/cgi-bin/hgTables). Analysis revealed that while 37% of the breakpoint regions were in heterochromatin regions, 63% of the breaks were in euchromatin regions (S2D [a] Fig). When a similar analysis was performed considering DNA of similar length from no-break control regions it was seen that 70% came from euchromatin and 30% came from heterochromatin (S2D [b] Fig). Further analysis of 70% no-break regions from the euchromatin, revealed that only 46% contained CpG and adjacent cryptic nonamers. In contrast, of the 63% of breakpoint regions harbored in the genic region, 74% had CpG and adjacent cryptic nonamers (S2D [c,d] Fig). This reveals that, the regions with breakpoints had a significantly higher percentage of CpGs and adjacent cryptic nonamers as compared to that of no-break regions when genic regions were analyzed (chi-square = 5.1, p-value = 0.02).

To investigate whether the co-occurrence of CpGs and cryptic nonamers seen was specific to the translocation of hematopoietic cell origin, we performed similar analysis with bins of 100 bp in patient breakpoints from nonlymphoid tumors. For this, we have analyzed 558 breakpoints covering 279 chromosomal translocations, in 145 genes, in nonlymphoid tumors. Results showed that while 7 cryptic nonamers, 1 CpG and 1 break were seen in the case of nonlymphoid translocations per 100 bp regions (chi square statistic = 53.99 and p-value = 1.9e-12), in the case of lymphoid translocations, the number of cryptic nonamers (13), CpGs (2) and breaks (8) occurred were significantly higher (Figs 2E and S2C). This suggests that the cryptic nonamer-CpG signature feature are characteristic of lymphoid translocation breakpoints. To find out the number of translocated genes common to cancers of lymphoid and nonlymphoid origin, we intersected all the genes with translocations and found only 1.5% common and 98.5% to be unique to both (S2E Fig). We were curious to find if common genes had differences in the sequence features; therefore, a 100 bp window around the breaks in both the cases were analysed. Interestingly, the number of nonamers per region were comparable, but the number of CpGs were double in the case of lymphoid cancers (1.3/region) as compared to nonlymphoid tumors (0.6/region) in those 1.5% overlapping genes (S2F and S2G Fig).

To confirm the presence of cryptic nonamers in RAG binding sites, we downloaded the wiggle files from a human thymocyte RAG1-ChIP experiment (GSM1701805) and observed RAG1 binding in the form of enriched peaks in TCRVβ and genes known to undergo translocations, which included ABL1, KMT2A FGFR1, etc. (S2H Fig). Importantly, the bound regions coincided with the translocation breakpoints that have been reported. Further, we observed that the stretch of nucleotides, where the enriched RAG1 peaks were present, consisted majorly of cryptic nonamers with 3–4 mismatches and no canonical nonamers (S2H Fig). Next, we investigated whether there was a preference of nucleotides within the cryptic nonamers for RAG1 to bind. To check for the conserved signature, we generated a position weight matrix logo which showed that in 60–70% of the cases, the string of adenines at 3^rd^, 4^th^, 5^th^ and 6^th^ positions (canonical nonamer being ACAAAAACC) and the C at 9^th^ position, were largely conserved (Fig 2F). Cryptic nonamers with at least 5 out of the nine 9 conserved nucleotides, were considered for further analysis [55,56].

Since we observed the clustering of patient breakpoints near cryptic nonamers and CpGs, we wondered whether distance between CpGs and cryptic nonamers is critical for genomic regions to become fragile. To address this, we analyzed the average distance between CpG and cryptic nonamers for fragile regions with that of random genomic regions, which are devoid of breakpoints. This was individually analyzed for cryptic nonamers with 2, 3 and 4 mismatches. Results showed that distance between CpGs and cryptic nonamers adjacent to breakpoints was within 10 to 80 bp. Interestingly, among these 73% of the CpG-nonamer pairs were <50 bp from breakpoint-rich regions for all chromosomes except chromosome 22. In contrast, this distance was 320–500 bp for nonfragile random regions, devoid of breakpoints (chi-squared value: 50–100, p-value <0.001) (Fig 2G). Thus, it appears that for a translocation breakpoint to occur, distance between CpGs and cryptic nonamers must be within 80 bp.

Overall, our results indicate that in several lymphoid cancers, chromosomal translocation breakpoints are flanked by cryptic nonamers and CpG sequences, indicating that the chromosomal fragility in those cases may be contributed by a RAG-mediated mechanism.

### RAGs can bind to cryptic nonamers and cleave at an adjacent T/G or U/G mismatch

Results presented above indicate that cryptic nonamers in the human genome when present close to a fragile region may facilitate aberrant RAG nicking leading to chromosomal rearrangements. To test this hypothesis, firstly core RAG1/2 were coexpressed in HEK293T cells, purified using GST or MBP tag and identity was confirmed through western blotting (S3A and S3B Fig). The functional activity of the purified RAGs was determined by its ability to bind and cleave at the standard RSS (S3C and S3D Fig).

**Fig 3:**
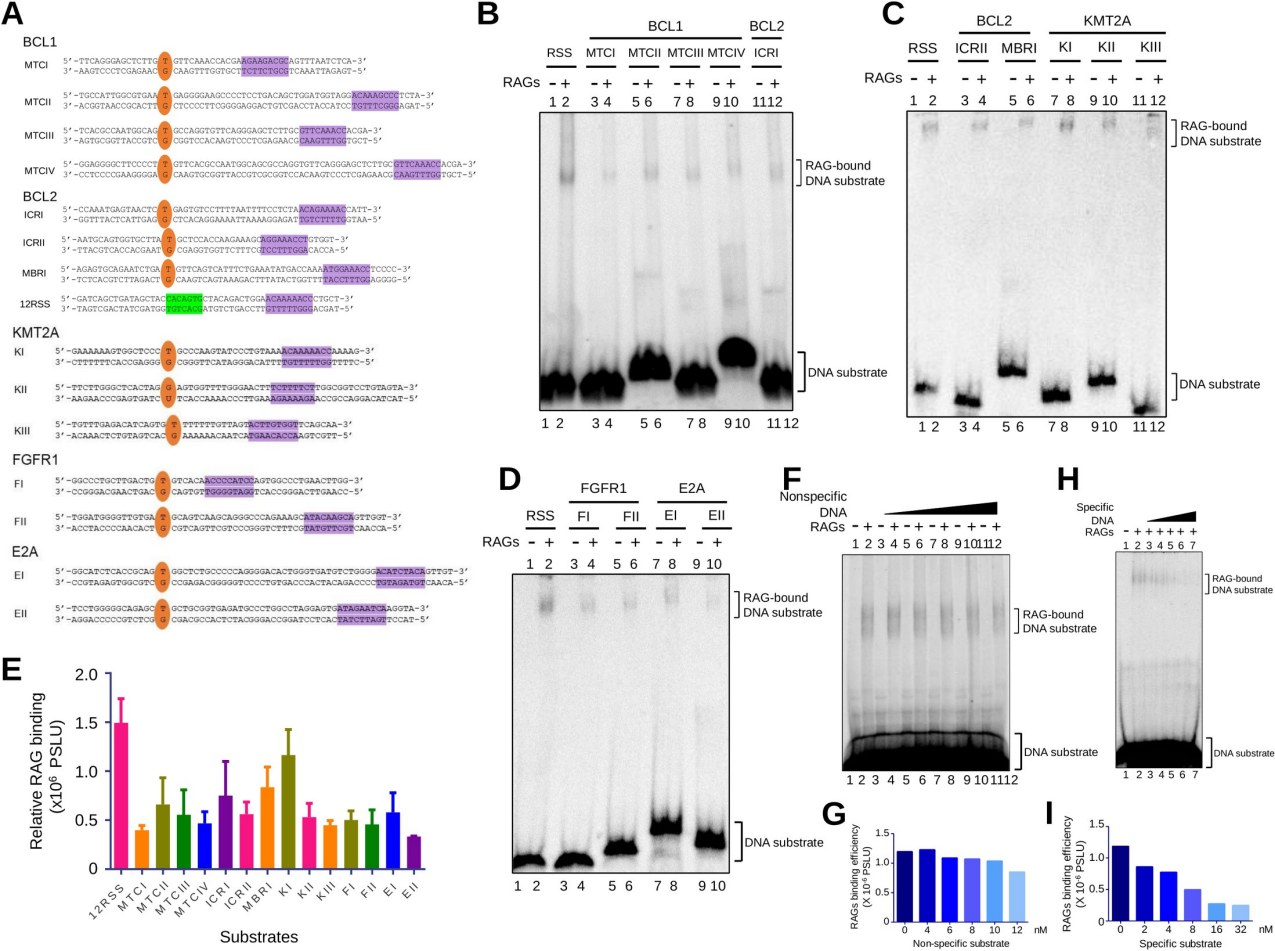
EMSA studies to investigate binding of RAGs with heteroduplex DNA containing T/G mismatch and a cryptic nonamer derived from patient breakpoint region. **A.** Schematic showing DNA substrates used in the assay. Oligomeric DNA are derived from translocation breakpoints of genes associated with lymphoid malignancy. Oligomers were annealed to form a double stranded DNA with either T/G or U/G mismatch (shown in orange), which mimics action of AID or spontaneous deamination of methylated CpG next to nearby cryptic nonamer (shown in purple). DNA showing 12RSS is also depicted. Heptamer in this DNA is indicated in green. **B-D.** Gel profile showing binding of cRAGs to heteroduplex DNA containing T/G mismatch and cryptic nonamer at breakpoint regions of genes, *BCL1* MTC and *BCL2* ICRI (B), *BCL2* ICRII, *BCL2* MBR and *KMT2A* (C) and *FGFR1* and *E2A* (D). **E.** Bar graph representing binding efficiency of cRAGs to DNA substrates. Each experiment was repeated atleast three times. Error bar was calculated as mean ± SEM. PSLU is photo-stimulated luminescence unit. **F-I.** EMSA studies showing evaluation of specificity of RAG binding to *BCL1* MTCII. EMSA was performed by incubating labeled DNA representing *BCL1* MTCII (8 nM) and cRAGs along with increasing concentration (0, 4, 6, 8, 10 and 12 nM) of unlabeled nonspecific DNA (F). EMSA with DNA containing *BCL1* MTCII (8 nM), cRAGs and increasing concentrations (0, 2, 4, 8, 16 and 32 nM) of the specific unlabeled DNA (H). Bar graphs showing quantification of RAG binding efficiency in presence of non-specific substrate (G) and specific substrate (I). In panels F and H, following RAG binding, the products were resolved on a 5% native polyacrylamide gel. The bands due to RAG binding are indicated by a square bracket in the gel.

To determine if the above hypothesis holds true, we designed double-stranded DNA (dsDNA) substrates derived from fragile regions of *BCL1 MTC* (Major translocation cluster), *BCL2 ICR* (Intermediate cluster region), *BCL2 MBR* (Major breakpoint region), *E2A*, *FGFR1*, and *KMT2A* (Figs 3A, S4A and S4B). DNA substrates were designed such that they harbor either a T/G or U/G mismatch mimicking the deamination of methylated cytosines/cytosine and cryptic/canonical nonamer at a distance of 6–42 bp from the mismatch (Figs 3A and S4A). In the case of each heteroduplex DNA, one of the oligomers was radiolabelled and annealed with its complementary strand (Figs 3A and S4A).

The radiolabelled heteroduplex DNA derived from the patient breakpoints were incubated with cRAGs at 25°C and the products were resolved on 5% native PAGE. EMSA results revealed that cRAGs bound to all DNA substrates containing T/G mismatch and cryptic nonamer (Fig 3B–3E). However, efficiency of RAG binding differed among fragile regions (Fig 3E). Nonetheless, the binding was distinct and appreciable in most of the heteroduplex DNA investigated when compared with the binding efficiency of 12RSS, the standard physiological substrate (Fig 3E). Among the substrates used, MTCI and EII showed lowest binding. Further specificity of the binding was confirmed by performing competition assay using one of the fragile sites, *BCL1* MTCII (8 nM) by incubating either with specific (unlabeled; 0, 2, 4, 8, 16 and 32 nM) or non-specific DNA (unlabeled; 0, 4, 6, 8, 10 and 12 nM) (Fig 3F–3I).

**Fig 4:**
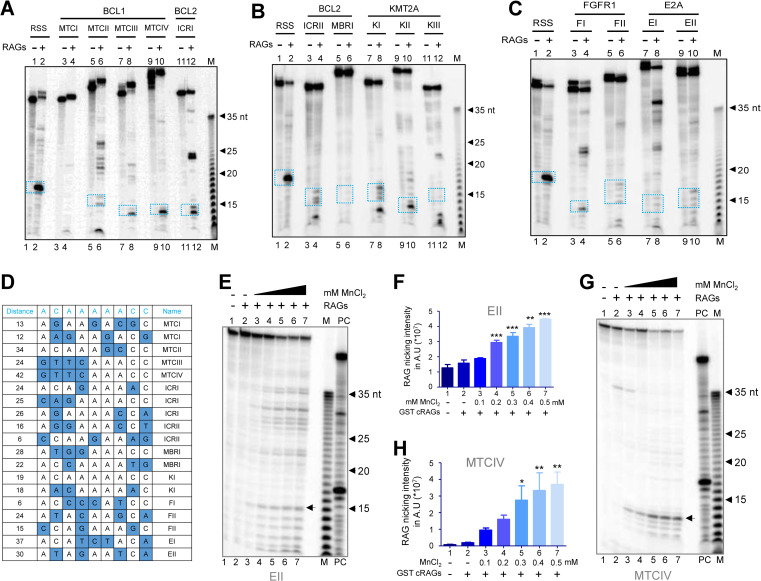
Analysis of cleavage by cRAGs on heteroduplex DNA containing T/G mismatch and a cryptic nonamer derived from patient breakpoint region. **A-C.** Evaluation of RAG cleavage and its efficacy on different DNA substrates derived from patient breakpoint regions when incubated with purified core RAGs. Gel profile showing the cleavage by cRAGs of heteroduplex DNA containing T/G mismatch and cryptic nonamer observed at breakpoint regions of *BCL1* MTC and *BCL2* ICRI (A), *BCL2* ICRII, MBR and *KMT2A* (B) and *FGFR1* and *E2A* (C). **D.** Tabular representation showing conservation in nucleotide sequences of cryptic nonamer in heteroduplex substrate used in the study. Blue color box represents deviation from canonical nonamer sequence. **E-G.** Effect of manganese ion on RAG cleavage of DNA heteroduplex containing T/G mismatch and cryptic nonamer. Gel profile showing RAG cleavage at increasing concentration of MnCl_2_ (100, 200, 300, 400 and 500 μM) for DNA substrate EII (E) and MTCIV (G). Bar graph showing quantification of RAG nicking intensity at increasing concentration of MnCl_2_ for substrate EII (F) and MTCIV (H). Error bar was calculated as mean ± SEM. *p < 0.05, **p < 0.005, ***p <0.0001. ns, not significant; AU is arbitrary unit. In panels, A-C, E and G, an oligomer representing standard 12RSS served as control. In these panels, 5’ end labelled, double-stranded DNA were incubated with purified cRAGs at 37°C for 1 h and products were resolved on 15% denaturing polyacrylamide gel. The specific cleavage product around 14–17 nt is indicated by either blue rectangular box (A-C) or arrowheads (E, G). M denotes 1 nt Klenow ladder.

To assess the ability of RAGs to cleave at fragile regions, purified RAG1/2 was incubated with the radiolabeled DNA substrates containing the breakpoints (37°C for 1 h) and the products were resolved on denaturing PAGE (15%). Results showed weak, but distinct RAG nicking at T/G mismatch (Fig 4A–4C). Although nicking occurred at the mismatch present at 15 nt position in every case, there were additional bands due to nicking of 1, 2 nt away from T/G mismatch (Fig 4A–4C). Examples include heteroduplex DNA substrates derived from KI, MTCII, III, IV etc. In the case of heteroduplex DNA KII, a distinct nicking was observed at 13 nt position followed by multiple faint nicking at proceeding nucleotides. It is important to note that predominant cleavage in some of these fragile regions occurred 1–3 nucleotide away from the mismatch, although there was nicking present exactly at the mismatch. Interestingly, even in the case of patient break points multiple breaks were seen in and around CpGs (S1 Fig). Thus, the presence of few bands due to cleavage at other sites on heteroduplex DNA apart from that at the mismatch perhaps may be a result of cleavage at breakpoints around CpGs, when cryptic nonamers with various sequences are placed nearby (Figs 4D and S1).

RAG cleavage buffer used in the present study was also supplemented with MnCl_2_ (5 mM), besides MgCl_2_ (5 mM). It is well established that Mn^2+^ ions can cause RAG mediated nicking in the absence of 12/23 RSS coupled pairing [57]. To study if the observed nicking can be seen at physiological concentration of MnCl_2_, the cleavage reactions were performed using a gradient of MnCl_2_ (100, 200, 300, 400 and 500 μM) (Fig 4E and 4G). In both fragile regions EII and MTCIV, the cleavage products were observed even at the lowest concentration of MnCl_2_ (100 μM), which is the physiological level of manganese within cells (Fig 4E–4H). Further, the efficiency of cleavage increased in a MnCl_2_ concentration dependent manner (Fig 4E–4H).

Spontaneous deamination is known to convert cytosine in the genome to uracil. Hence, we tested if RAG cleavage could occur at U/G mismatches and assessed the impact of a nonamer adjacent to it (S4B–S4E Fig). Heteroduplex DNA with U/G mismatches alone or with a canonical nonamer (separated by 6, 12 and 23 bp) (S4B Fig) were designed and synthesized. Binding and cleavage assays showed that RAGs could bind (S4C Fig) and cleave (S4D–S4E Fig) at U/G mismatches on heteroduplex DNA when canonical nonamer was present. Cleavage at U/G mismatch progressively decreased with an increase in distance from the canonical nonamer (S4D–S4E Fig), where U/G mismatch 6 bp away from nonamer showed the highest cleavage. The control sequence with only U/G mismatch showed no cleavage (S4D Fig, lanes 3,4). Similarly, a random ds DNA also had no cleavage upon incubation with RAGs (S4E Fig, lanes 7,8).

### Presence of cryptic nonamer is critical for RAG mediated cleavage at CpGs associated with mismatches

**Fig 5:**
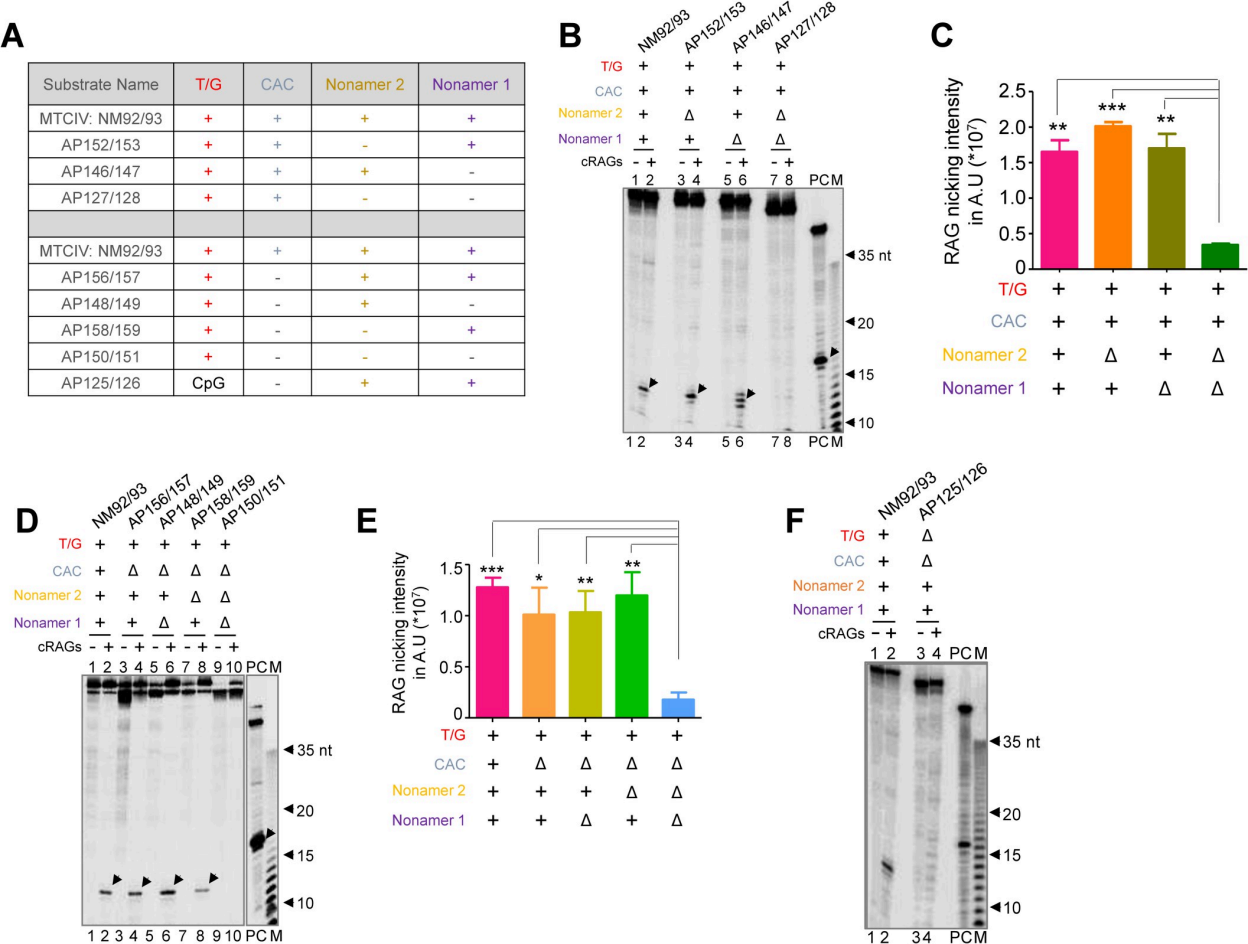
Evaluation of RAG nicking specificity on DNA substrates with T/G mismatch when an adjacent cryptic nonamer/s is present and impact of CAC on efficiency of RAG mediated cleavage. **A.** Table indicating mutations generated on DNA substrates based on MTCIV having mutation either on CAC, cryptic nonamer 1, cryptic nonamer 2 or both. Refer S5A Fig for details. **B.** Gel profile showing RAG nicking on MTCIV derived DNA substrates with mutations either in one or both the cryptic nonamers. Δ denotes mutation in the nonamer 1, 2 or CAC sequence. **C.** Bar graph showing quantification of RAG nicking intensity on MTCIV derived DNA substrates with mutated cryptic nonamers. **D.** Gel profile showing RAG nicking on MTCIV derived DNA substrates when either CAC, cryptic nonamer 1 or 2 were mutated. **E.** Bar graph showing quantification of RAG nicking intensity on MTCIV derived DNA substrates with mutated CAC and cryptic nonamers as indicated. In both panels C and E, error bar was calculated as mean ± SEM. *p < 0.05, **p < 0.005, ***p <0.0001. ns, not significant; AU is arbitrary unit. **F.** Comparison of gel profile showing RAG nicking on MTCIV derived DNA substrates containing either CpG or T/G mismatch along with cryptic nonamer 1 and 2. In panels, B, D, F, ‘PC’ is positive control in which RAG cleavage reaction was performed using labelled 12RSS. ‘M’ is 1 nt Klenow ladder. The specific cleavage product around 12–13 nt is indicated by arrowheads.

To determine the dependence of RAG mediated cleavage on heteroduplex DNA with a T/G mismatch on cryptic nonamers, we designed DNA substrates derived from MTCIV containing T/G mismatch with one of the 2 cryptic nonamers mutated or both (Fig 5A and S5). Results revealed that RAG nicking was completely abolished only when both cryptic nonamers were mutated (Fig 5B and 5C). Mutation in either cryptic nonamer 1 or 2 did not affect overall cleavage efficiency compared to control, although there was difference in the cleavage in case of substrate DNA in which cryptic nonamer 1 was mutated (Fig 5B). This suggests that presence of at least one cryptic nonamer is important for RAG cleavage.

Previous studies have shown that mere presence of ‘CAC’ of heptamer associated with recombination signal sequence (RSS) involved in V(D)J recombination is sufficient for RAG mediated cleavage [10,18]. Since there is a CAC sequence present just 3 nt downstream to T/G mismatch derived from MTCIV, we wondered whether this could have an impact on observed RAG cleavage at T/G mismatch. To address this, oligomeric DNA were designed with mutation in CAC alone, CAC and cryptic nonamer 1, CAC and cryptic nonamer 2 or all three (Figs 5A and S5). Further, we did not observe any significant change in RAG nicking when CAC and either of the cryptic nonamers were mutated (Fig 5D and 5E). However, no cleavage was observed where all three were mutated (Fig 5D and 5E). Besides, we observed significantly reduced cleavage when the T/G mismatch was replaced with a CpG site (Fig 5F). Taken together, our results suggest that RAG nicking occurs only when T/G mismatch is placed with either of the cryptic nonamers. Further, removal of CAC did not affect RAG nicking activity.

To address the question whether RAG nicking could occur at regions which are not known to undergo rearrangements in lymphoid cancer patients, but possesses CpG and cryptic nonamers, we selected 3 random regions (ERP44, CNTN5 and an unannotated region) of the human genome (S6A Fig). Oligomeric DNA containing either CpG or T/G mismatch with adjacent cryptic nonamer was designed and synthesized as described before and subjected to RAG cleavage assay. Results revealed no specific RAG nicking in any of the DNA substrates investigated at the mismatch or around it (S6B and S6C Fig). However, there were multiple faint bands due to nonspecific nicking in presence of RAGs particularly in the case of Region 1 when heteroduplex DNA was present (S6B Fig). To gauge whether overall AT/ GC composition makes region susceptible to RAG mediated fragility, we selected 2 AT (two unannotated regions) and GC (DYNC2H1 and NDOR1) rich regions each and designed oligomeric DNA substrates harboring either CpG or T/G mismatch nearby cryptic nonamer (S6D Fig). No RAG specific nicking was observed at expected position in all the substrates, although in both AT rich regions, non-specific faint nicking was seen in presence of RAGs (S6E and S6F Fig).

Thus, above results suggest that the fragile sites reported in lymphoid malignancies, present outside the Ig/TCR loci could be recognized by RAGs through a cryptic nonamer and cleaved at an adjacent mismatch if deamination occurred at a CpG or cytosine site. However, the efficiency of RAG cleavage varied and was much lower than standard RSS. Considering that such translocations occur only at a low frequency, the observed weak RAG1 cleavage activity is understandable.

### RAG binding at fragile regions is due to nonamer binding domain of RAG1

**Fig 6:**
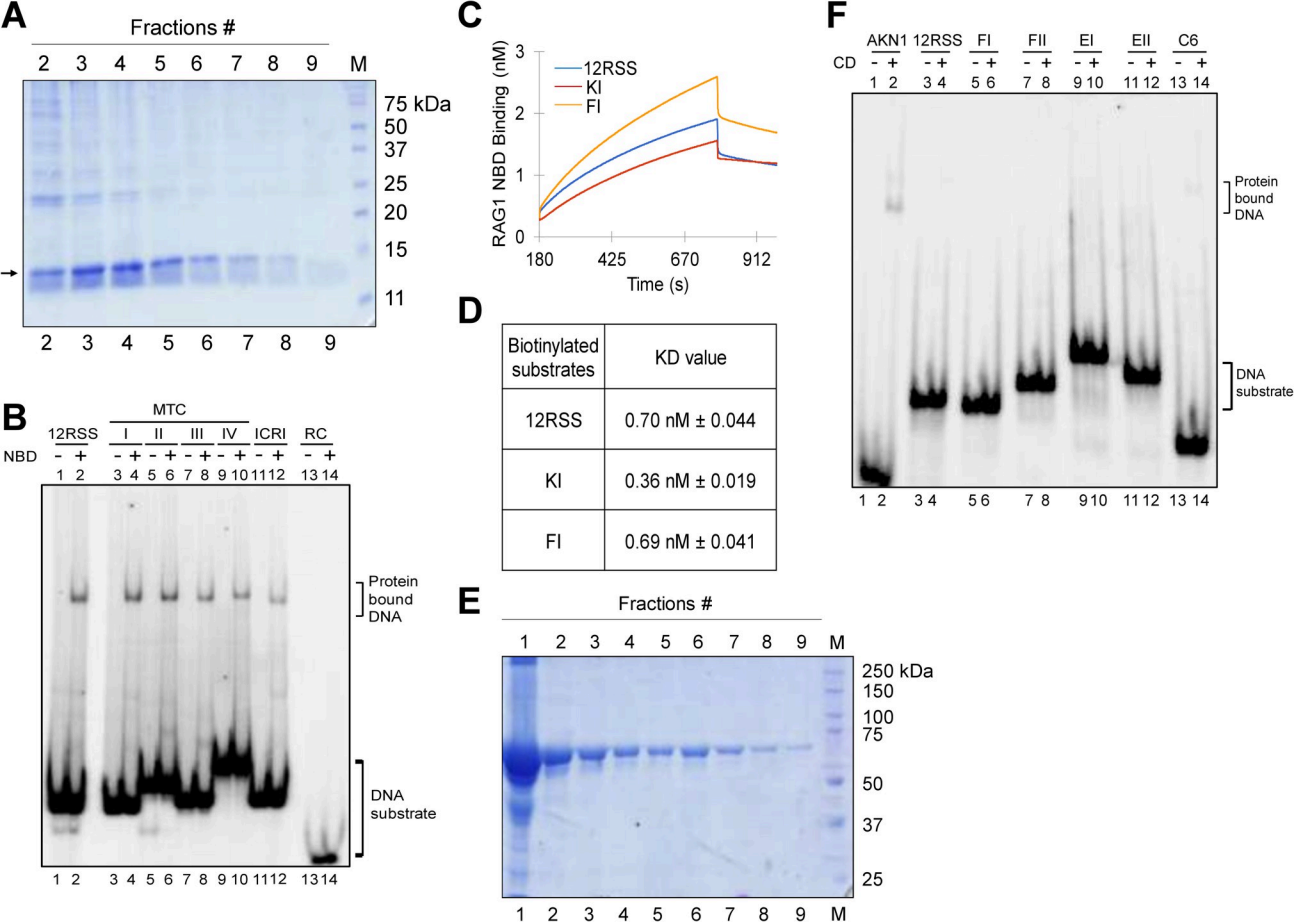
Affinity of various domains of RAGs to heteroduplex DNA containing U/G or T/G mismatch. **A.** Overexpression and purification of nonamer binding domain (NBD) of RAG1. The purified protein is indicated by an arrowhead. “M” is molecular weight ladder. **B.** Gel profile showing binding of NBD to heteroduplex DNA containing T/G mismatch and cryptic nonamer observed at breakpoint region of *BCL1* MTC and *BCL2* ICR. “RC” is random DNA control. **C, D.** Biolayer interferometry studies showing binding of NBD of RAG1 to selected heteroduplex DNA substrates containing T/G mismatch and cryptic nonamer representing breakpoint region of KMT2A (KI), FGFR1 (FI). Biolayer interferometry sensorgrams depicts the real time binding of NBD of RAG1 (0.9 nM) to heteroduplex DNA substrates KI, FI and, 12RSS immobilized to SAX sensors. The sensorgrams curves depict the association, followed by dissociation of NBD-RAG1 (C). The real time binding curves were used to compute equilibrium dissociation constant (KD) by globally fitting the rate equation for 1:1 kinetics to the data. (D). **E.** Overexpression and purification of central domain of RAG1. “M” is molecular weight ladder**. F.** Gel profile showing binding of RAG1 central domain to heteroduplex DNA containing T/G mismatch and cryptic nonamer derived from breakpoint regions of FGFR1 (FI, FII) and E2A (EI, EII). AKN1 represents single-stranded 12RSS. C6 represent a heteroduplex DNA with cytosine (6 nt) at the bubble region.

Having established that RAGs could recognize and bind to the cryptic nonamer present in the fragile regions reported in lymphoid malignancies, we were interested in studying the domain of RAG1 responsible for its binding to heteroduplex DNA. EMSA studies were performed with heteroduplex DNA harboring either T/G or U/G mismatch and cryptic/canonical nonamer (Figs 3A and S4B) in presence of purified central domain, NBD of RAG1, or NBD-deleted cRAG1 (Figs 6 and S7).

Results showed that purified NBD could recognize and bind to the cryptic nonamers present in the fragile regions (Fig 6A and 6B). Efficient binding of NBD to heteroduplex DNA possessing cryptic nonamers were seen in the case of MTCI, MTCII, MTCIII and MTCIV and the efficacy of binding was comparable to that of 12RSS. Among the substrates studied, ICRI had the lowest intensity, while random ds DNA control region (RC) showed no NBD binding (Fig 6B). These results indicate that nonamer binding domain could bind and differentiate between the variation among the sequences. In case of heteroduplex DNA containing U/G mismatch and canonical nonamer, there was an enhanced efficiency of NBD binding with the increase in distance between the mismatch and nonamer (S7A and S7B Fig). Importantly, NBD failed to bind to DNA when there was no nonamer reinforcing the specificity of binding (S7B Fig, lanes 1, 2). Thus, our results suggest that RAGs can recognize and bind to the fragile regions by virtue of NBD of RAG1 and the efficiency of binding decreased when the cryptic nonamer was placed far apart.

Biolayer interferometry (BLI), is an analytical technique based on change in light interference, which allows studying macromolecular interactions. We used BLI to study the interaction of NBD to oligomeric DNA containing T/G mismatch and cryptic nonamer derived from fragile regions (KI, FI). Oligomeric DNA containing RSS served as the control. DNA substrates were immobilized onto SAX (High Precision Streptavidin) sensors and NBD was used as an analyte. BLI allows to measure the dissociation constant (K_D_) of binding of NBD with the DNA substrates. NBD bound with a K_D_ of 0.7 nM, when it was incubated with 12RSS, while the K_D_ was 0.36 nM and 0.69 nM for KI and FI, respectively (Fig 6C and 6D). The K_D_ of KI was lower than 12RSS, suggesting a stronger interaction, which is understandable as it possessed a canonical nonamer. Interestingly, even the substrate FI, which possess a cryptic nonamer also exhibited binding affinity comparable to that of 12RSS (Fig 6D). Consistent to this result, prominent RAG cleavage at the mismatch was also observed (Fig 4B, lanes 7,8 and 4C, lanes 3, 4). These results confirm that binding and cleavage of fragile region by RAGs is mediated through its nonamer binding domain.

The NBD deleted cRAGs failed to bind to heteroduplex DNA containing U/G mismatches and canonical nonamer (S7A and S7D Fig, lanes 3–10) though it bound weakly to heteroduplex DNA containing 6 nt mismatch (S7D Fig, lanes 1, 2), which is understandable as cRAGs is known to function as a structure-specific nuclease [37,58]. In contrast, NBD exhibited a significant enhancement in binding to heteroduplex DNA containing U/G mismatches when nonamer was present (S7B Fig), which was consistent with the above observation.

The central domain of RAG1 did not bind to fragile regions with T/G or U/G mismatches when cryptic or canonical nonamer were used (Figs 6E, 6F and S7C). According to the crystal structure published recently, RAG1 CD domain contains Pre-R, RNH and ZnC_2_ modules which begins from amino acids 528 to 760 of full length RAG1 [59]. The observed failure of binding is not surprising as the central domain is known to have a very low efficiency of binding to dsDNA as compared to ssDNA [58,60]. Thus, our findings suggest that NBD, but not CD of RAG1 is responsible for RAG binding at cryptic nonamer present adjacent to the CpG site.

### RAGs can bind to fragile regions that harbors CpGs and cryptic nonamer within cells

Above results suggest a possible RAG mediated fragility at chromosomal translocation breakpoint regions when CpGs and cryptic nonamers were placed within 80 bp from each other. Therefore, we investigated whether RAGs can bind to such regions within cells. We selected Nalm6, a pre-B leukemic cell line, known to express RAGs, for our analysis. 8 fragile regions known to undergo translocation viz., *BCL1 MTC*, *BCL2 ICR*, *BCL2 MBR*, *E2A*, *FGFR1*, *KMT2A*, *PDGFRA and ABL1* were selected for the study. Random control gene sequences devoid of CpG and cryptic nonamer enrichment, namely, *VEGF*, *MyoD*, *Myc* were tested, while IgHV, IgHD served as positive control.

**Fig 7:**
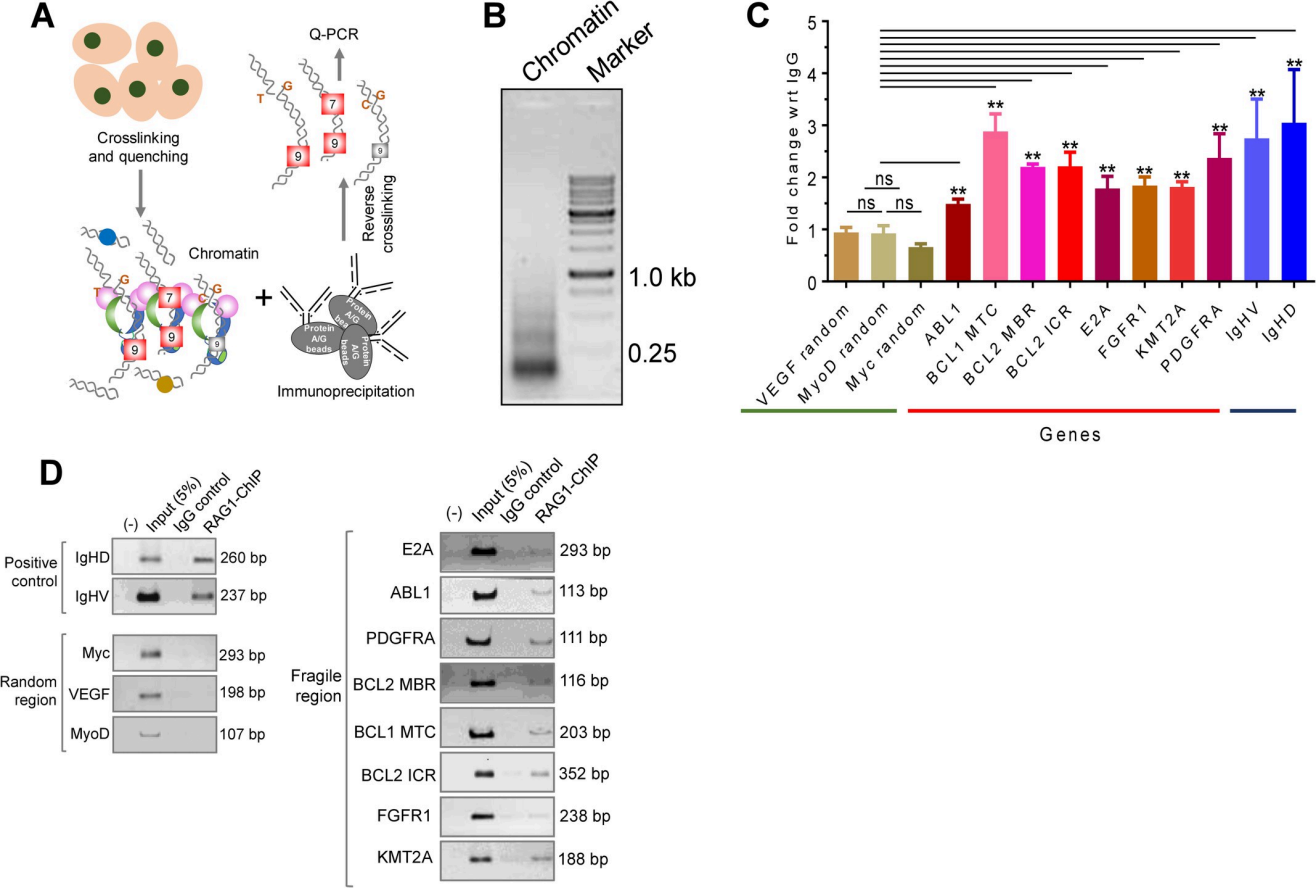
Chromatin immunoprecipitation of RAG1 for fragile genomic regions rich in CpGs and cryptic nonamers. **A.** Pictorial representation of ChIP. Nalm6 cells were crosslinked, chromatin was isolated, and immunoprecipitated using RAG1 antibody. The chromatin samples were used for either semi-quantitative PCR or qPCR using specific primers against fragile regions and other region of interest. **B.** Chromatin digestion of Nalm6 cell line used for RAG1 ChIP. **C.** Graph illustrating qPCR analysis of RAG1 ChIP of gene regions rich in CpGs and cryptic nonamers. Result was plotted as fold enrichment with respect to IgG for each gene analyzed. Each experiment was repeated 3 times independently. Statistical analysis was performed by non-parametric two tailed T test assuming non-gaussian distribution (ns, not significant, **p<0.01). **D.** Semi-quantitative PCR following RAG1 ChIP in Nalm6 cells. 13 regions from various genes were investigated for binding. *IgHV* and *IgHD* are positive control DNA sequences, which represent 12RSS and 23RSS, respectively. *ABL1*, *BCL1 MTC*, *BCL2 MBR*, *BCL2 ICR*, *E2A*, *FGFR1*, *KMT2A*, *PDGFRA* are DNA sequences enriched in CpGs and nonamers. *VEGF*, *MyoD* and *Myc* are negative control random sequences devoid of CpGs, nonamers and breakpoint enrichment. ‘(-)’ indicates ‘No Template Control’. ‘5% Input’ indicates 5% of total input chromatin used in RAG1 ChIP. ‘IgG’ indicates normal rabbit IgG antibody control used in ChIP. ‘RAG1’ indicates RAG1 ChIP sample.

For chromatin immunoprecipitation (ChIP), chromatin from Nalm6 cells was crosslinked, cells were lysed and the chromatin was digested with micrococcal nuclease, followed by sonication to obtain DNA fragments of 100–500 bp size. (Figs 7A, 7B and S8A). ChIP was performed against RAG1 pulled down. DNA was analyzed by real time PCR using appropriate primers for detecting regions of interest. Results showed a RAG-specific enrichment for all the tested fragile regions compared to IgG control, although the efficiency of enrichment varied among fragile regions (Fig 7C). In contrast, such an enrichment was not observed when random control regions such as VEGF and MyoD were analyzed using qPCR (Fig 7C). As expected, the positive control regions, IgHV and IgHD showed approximately 3-fold change with respect to IgG control. Importantly, all *BCL2* regions selected for the study showed >2-fold enrichment when compared to its IgG control (P value < 0.01) (Fig 7C). Representative amplification curves with melt curves and average Ct values are also presented (S8B, S8C and S9 Figs). Thus, above results suggest that RAGs can indeed bind to the fragile regions within cells.

The results were also confirmed using semi-quantitative PCR of ChIP-ed DNA (Fig 7D). We observed that while the positive controls, particularly IgHD, showed a strong amplification, for random control regions the bands were undetectable. Interestingly, all the fragile regions showed robust amplification, which was comparable to that of positive controls (Fig 7D). Thus, RAG1 indeed binds to the fragile regions containing cryptic nonamers and CpGs within cells, indicating the possibility of RAG mediated cleavage at genome level.

### Chromosomal fragile regions containing cryptic nonamers with CpGs and RSS present in the V(D)J locus do not undergo synapsis

**Fig 8:**
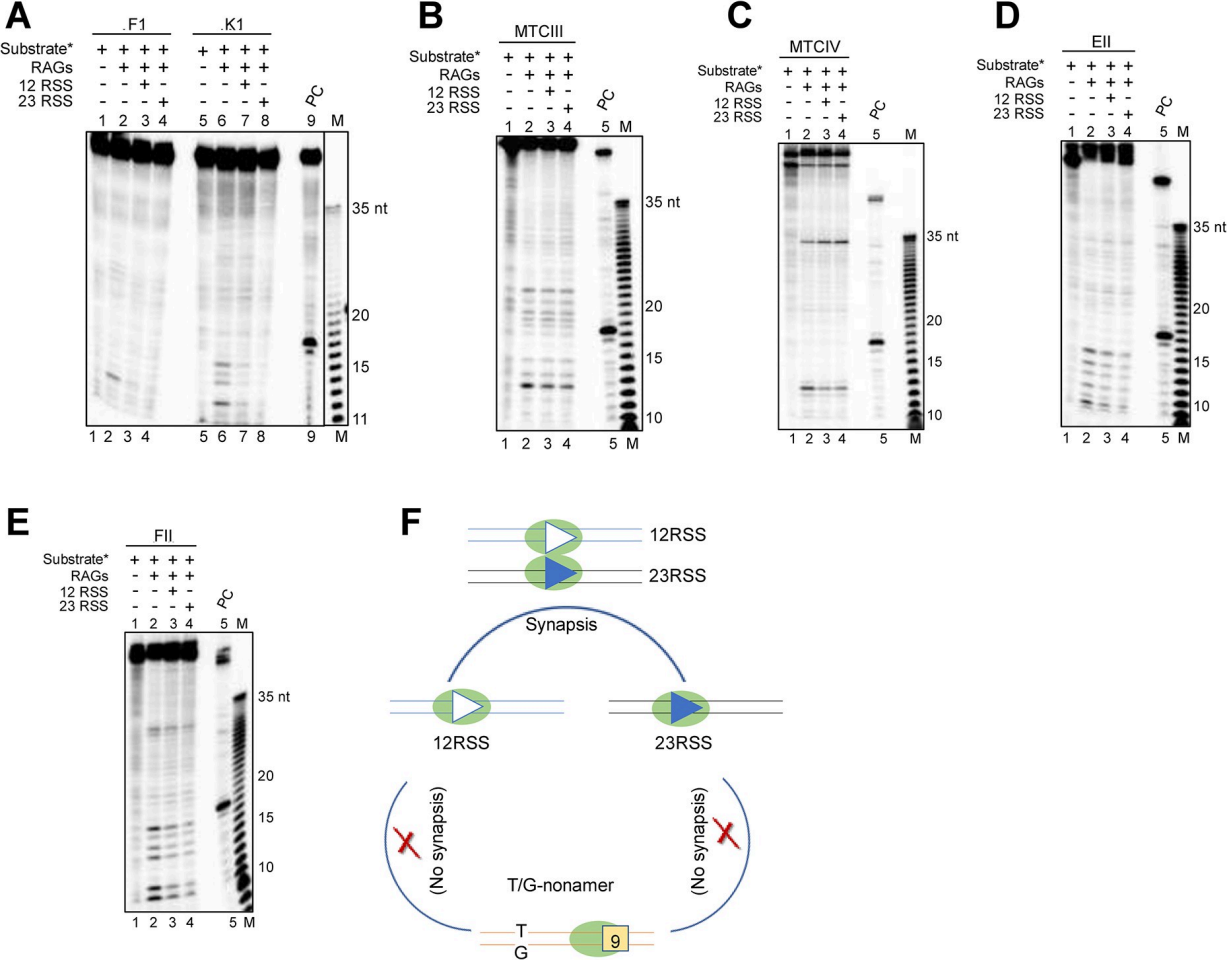
Intermolecular coupled cleavage by RAGs on heteroduplex DNA containing T/G mismatch and a cryptic nonamer on DNA derived from patient breakpoint region in presence of a 12 or 23RSS. **A-E.** Coupled RAG cleavage between 12/23 RSS and fragile regions of FI, KI (A), MTCIII (B), MTCIV (C), EII (D), FII (E). The reactions contained [γ-^32^P] labeled oligomers derived from fragile region was paired with cold 12 or 23RSS and incubated with core RAGs at 37°C for 1 h and the samples were analyzed on a 15% denaturing polyacrylamide gel. FI and FII are from *FGFR1*, KI is from *KMT2A*, EII is from *E2A*, MTCIII and MTCIV are from BCL1. ‘PC’ is RAG cleavage reaction using labelled 12RSS. ‘M’ is Klenow ladder used as 1 nt ladder. **F.** Pictorial representation summarizing requirement of synapsis for RAG induced cleavage at fragile regions. While synapsis occurs between 12RSS with 23RSS, there was no synapsis observed between a T/G mismatch-nonamer substrate with a 12RSS or 23RSS.

During V(D)J recombination, following RAG binding, a paired complex involving both 12 and 23RSS is formed [61]. We wondered if such complex is formed between the standard 12 or 23RSS and fragile regions containing a deaminated CpG and a cryptic nonamer. To test this, coupled RAG cleavage was performed with radiolabeled DNA substrates FI, KI, MTCIII, MTCIV, EII, FII (Fig 8A–8E) and unlabeled 12 or 23RSS. Results showed that all substrates exhibited RAG mediated cleavage at the T/G mismatch present near cryptic nonamer, which was not enhanced in presence of standard 12/23RSS (Fig 8A–8E). This suggests that coupled cleavage between a standard RSS in the Ig/TCR loci and patient breakpoint adjacent to a cryptic nonamer and CpG does not occur (Fig 8F). The decrease seen in the cleavage band in the presence of 12 or 23RSS could be due to the use of cold RSS as a substrate instead of the radiolabeled RSS by cRAGs.

### Reconstitution of RAG cleavage at fragile regions reveal the involvement of AID induced mismatches at CpGs

**Fig 9:**
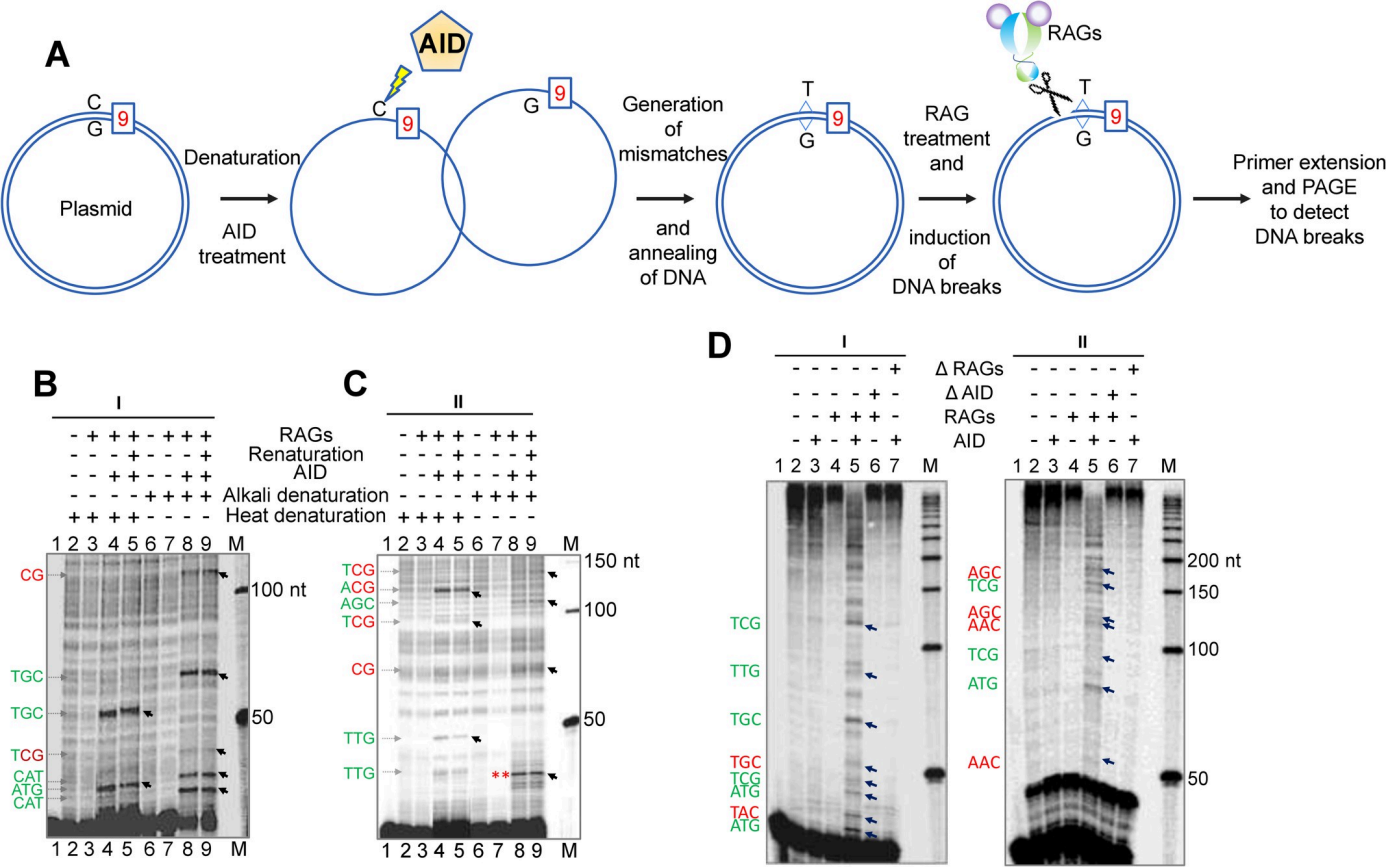
*In vitro* reconstitution assay for RAG cleavage upon AID treatment on fragile region harboring cryptic nonamer and CpG sites. **A.** Schematic of the experimental procedure. **B-C.** Primer extension profile of reconstitution assay on pAP15 (KMT2A fragile region) using ‘I’ forward primer AP72 (B) and reverse primer AP73 ‘II’ (C). Pause site sequences are provided on left of the gel. Red color indicates CpG sites, WRC/GYW motifs are in green, ** indicates potential RAG mediated cleavage at secondary DNA structure. **D.** Primer extension profile of reconstitution assay when either AID deaminase domain mutant (ΔAID) or nuclease dead mutant of cRAG1 and cRAG2 (ΔRAGs) was used. The pause sites are indicated by arrows in the gel. “M” indicates 50 bp ladder.

We designed reconstitution assay for RAG cleavage following AID treatment on fragile region harboring cryptic nonamers and CpG sites. AID mediated deamination at CpG sites could result in U/G or T/G mismatches on a plasmid depending on the methylation status, which could be nicked by RAGs and scored. Towards this, one of the fragile regions, *KMT2A* was cloned into a plasmid to generate pAP15 and used for reconstitution assay as outlined (Fig 9A). GST tagged AID was overexpressed, purified and activity was confirmed by deamination assay (S10B–S10F Fig). Plasmid harboring *KMT2A* was denatured to generate single-stranded DNA and was incubated with purified AID (Figs 9A, S10A and S10B). Following AID treatment, deaminated plasmid was renatured and incubated with RAGs. Reaction products were purified, and RAG induced breaks were detected using primer extension followed by electrophoresis on denaturing PAGE (Figs 9A, S10A and S10D). There were four CpG sites and multiple WRC (A/T A/G C)/GYW (G C/T A/T) motifs were present in the *KMT2A* region in pAP15. Interestingly, two pause sites observed were at CpG sites and matched with the patient breakpoints reported in literature. The observed pause sites were ~110 nt, when forward primer (AP72) was used, and at ~75 nt when reverse primer (AP73) was used. Importantly, on both the strands there were adjacent cryptic nonamers, which were 18 nt each away from the CpG, for top and bottoms strands. Besides, additional RAG induced breaks were detected as pause sites, which were matched to WRC or GYW motifs (Fig 9B and 9C). WRC/GYW motifs are established deamination sites for AID [62]. Such RAG induced breaks were absent when the plasmid was not incubated with AID, suggesting that AID induced deamination was important for RAG activity (Figs 9B lanes 3,7 and 7C lanes 3,7). We further used deaminase domain mutant of AID and nuclease dead mutant of RAGs to confirm observed pause sites are indeed because of a combined action of AID and RAGs (S11A–S11E Fig). Importantly, pause sites were observed only when wild type AID and RAG proteins were used. There were no specific pause sites when either AID or RAG alone was used for the assay (Fig 9D). These results indicate that AID mediated deamination of CpGs and cytosines at fragile regions when present adjacent to cryptic nonamers can become sites for RAG induced breaks.

### RAGs could induce a second break on complementary strand opposite to first one leading to generation of a double-strand DNA break

**Fig 10:**
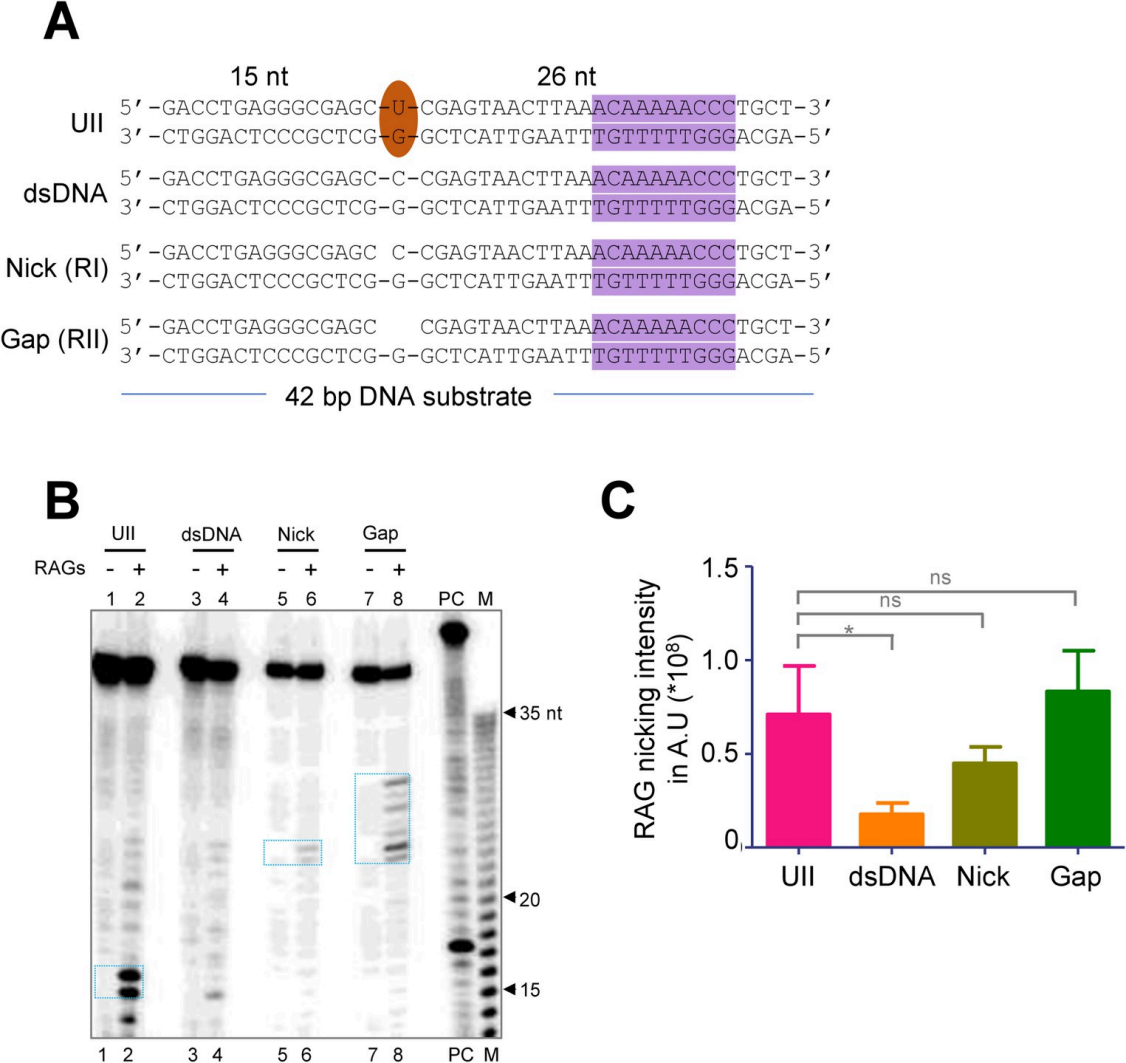
Evaluation of RAG mediated cleavage on DNA substrates mimicking intermediate stages of DNA repair with a nick or a gap. **A.** Schematic showing sequences of double-stranded DNA (dsDNA), nick or gap substrates designed to mimic repair intermediates following repair of U/G mismatches by BER or MMR pathway. Brown oval indicates U/G mismatch, purple highlight indicates nonamer. **B.** RAG cleavage on substrates harboring either U/G mismatch (UII), double-stranded DNA (dsDNA), nick or gap substrates designed to mimic repair intermediates following repair of U/G mismatches. ‘PC’ is RAG cleavage reaction using labelled 12RSS. ‘M’ is 1 nt ladder. **C.** Quantification showing RAG cleavage efficacy on DNA substrates bearing U/G mismatches with nonamer, ds DNA with nonamer alone, nick, or gap. AU is Arbitrary Unit.

AID mediated deamination of cytosine leading to a U/G mismatch (or T/G mismatch if it is methylated cytosine) when occurs inside the cells is normally repaired by base excision repair (BER) resulting in the generation of abasic site which can get converted as longer single-stranded gap by mismatch repair pathway (MMR) [63,64] in some instances. Considering that this can act as another mode of generation of a single-strand break, besides RAG induced direct nicking, we explored the possibility of RAGs inducing a second nick in the complementary strand. To test whether such repair intermediates (nick/gap) are readily converted by RAG1/RAG2 into double-strand breaks, we designed oligomeric substrate RII (with a nick) and RII (with a gap) which mimic repair intermediates generated during BER and MMR, respectively based on UII heteroduplex DNA used in the study (Figs 10A and S4B). Double-stranded DNA with no U/G mismatch derived from UII served as control (Fig 10A). RAG cleavage assay using these substrates showed cleaved products at ~26 nt position and at neighbouring nucleotides (Fig 10B) when the complementary strand was radiolabeled. These bands can be generated only if RAGs induce additional nick/s at the already existing nick or gap further suggesting that such repair intermediates when present on a DNA can be converted to DSBs by RAGs (Fig 10C). It is also noted that when the substrate DNA possessed a gap, the cleavage by RAGs occurred at multiple positions in the complementary strand (Fig 10B, lanes 7,8). Further, there was no significant RAG nicking observed when the U/G mismatch was absent, although a canonical nonamer was present (Fig 10B).

### Detection of double-stranded breaks inside the cell by ligation-mediated PCR (LM PCR)

**Fig 11:**
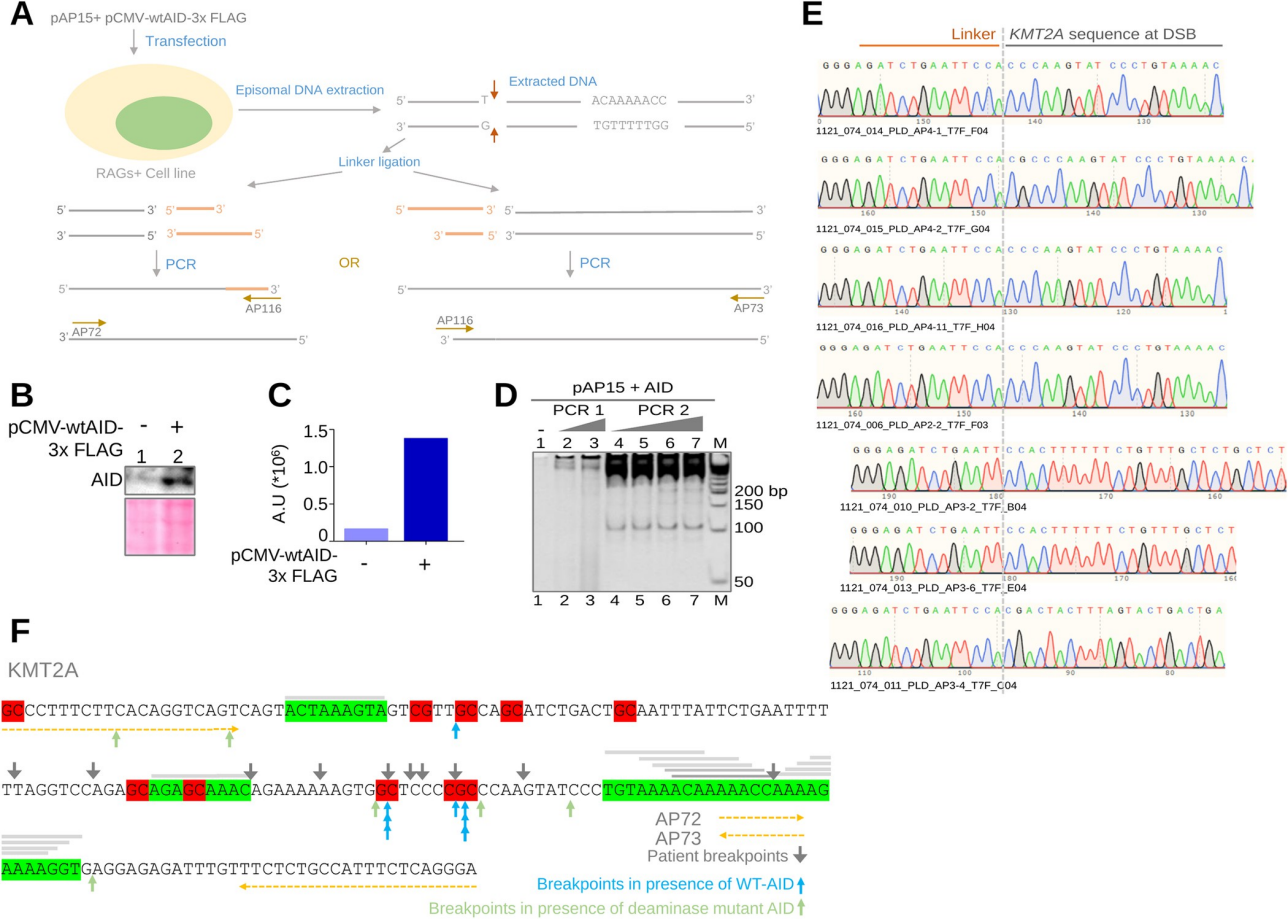
Detection of double-stranded DNA breaks in KMT2A fragile region inside cells by ligation mediated PCR (LM-PCR). **A.** Schematic diagram showing experimental procedure used for detection of DSBs. Construct pAP15 containing KMT2A fragile region was cotransfected with pCMV-wtAID-3x FLAG in RAG expressing T-cell leukemia cell line, CEM to detect DSBs in presence of both AID and RAGs. **B.** Western blot analysis to examine overexpression of AID in CEM cells. CEM cells (4*10^5^) were transfected with pCMV-wtAID-3x FLAG construct (6 μg) to overexpress AID. Cell extracts were prepared at 48 h post-transfection and loaded on 10% SDS-PAGE. Blots were probed with AID antibody. **C.** Bar graph showing quantification of AID expression after normalizing with ponceau stained membrane as loading control. **D.** LM-PCR analysis of KMT2A fragile region in CEM cells following AID overexpression. Episomal DNA was extracted at 48 h and subjected to linker ligation. Ligated DNA was used for PCR amplification using gene specific primer (AP73) and linker specific primer (AP116). Following first PCR, reamplification (2^nd^ PCR) was also performed. **E.** Chromatogram of ligated KMT2A sequences obtained following cloning and sequencing of LM-PCR products. Left side of the chromatogram represents linker sequence whereas right side indicates KMT2A sequence. **F.** Schematic presentation of KMT2A breakpoint sequence seen in patients compared to LM-PCR mapped breakpoints based on our assay. Red highlighted sequence indicates CpG sites. Green highlighted sequence indicates cryptic nonamers. Different intensity grey lines on the top of the sequence correspond to various permutations of cryptic nonamers. Grey downward arrows represent patient breakpoints. Blue and light green upward arrows indicate breakpoints determined based on LM-PCR following AID overexpression and deaminase domain mutant AID overexpression, respectively.

We employed ligation-mediated PCR approach to test whether RAG induced DSBs can be detected within cells at a fragile region, when CpGs and cryptic nonamers are present (Fig 11A). For this, the T-cell leukemic cell line, CEM, known to have RAG1 expression [9,65] was transfected with extrachromosomal episomal construct, pAP15 containing KMT2A fragile region along with AID overexpression construct pCMV-wtAID-3x FLAG (Fig 11A). In an independent experiment deaminase domain mutant AID was overexpressed along with pAP15 in CEM cell line as a control. Following confirmation of AID overexpression by western blotting, episomal DNA was harvested and subjected to linker ligation (Fig 11A–11C). PCR was performed on ligated DNA using linker specific primer and gene specific primer and products were resolved on denaturing PAGE (Fig 11D). Results revealed multiple amplification products, which were cloned and sequenced. Particularly distinct amplification product was seen around 100 bp and weak amplification products at 150 and 200 bp positions upon AID overexpression (Fig 11D). Sequencing results revealed joining of linker to CpGs adjacent to cryptic nonamer cluster when wild type AID was overexpressed indicating accumulation of DSBs at and around the CpG sites. (Figs 11E, 11F and S11C). In the control with deaminase mutant AID, many of the DSBs obtained were at CAC/ GTG, while few were near CCC. This suggest in absence of AID, RAG mediated nicking is the main mechanism of fragility whereas when both AID and RAGs are present, CpGs adjacent to cryptic nonamers are more prone to DSB (Fig 11F). Interestingly, all the DSBs identified in our study overlapped with breakpoints reported in patients (Fig 11F).

We also performed similar analysis in the pre-B cell line (Nalm6), where robust expression of RAGs and low expression of AID are known to be present [66]. Cloning and sequencing of PCR products revealed fusion of linkers at proximity of CpG sites with cryptic nonamers in KMT2A fragile region (S12A and S12B Fig) indicating that linker ligated to DSBs present in the fragile region following transfection. Importantly, several DSBs identified overlapped with patient breakpoints even in this case as well (S12A Fig). This suggests that CpGs present adjacent to cryptic nonamer rich fragile region are susceptible to RAG mediated cleavage following AID mediated deamination of cytosines or methylated cytosines, which could result into DSBs inside the cell when both the proteins work in tandem.

**Fig 12:**
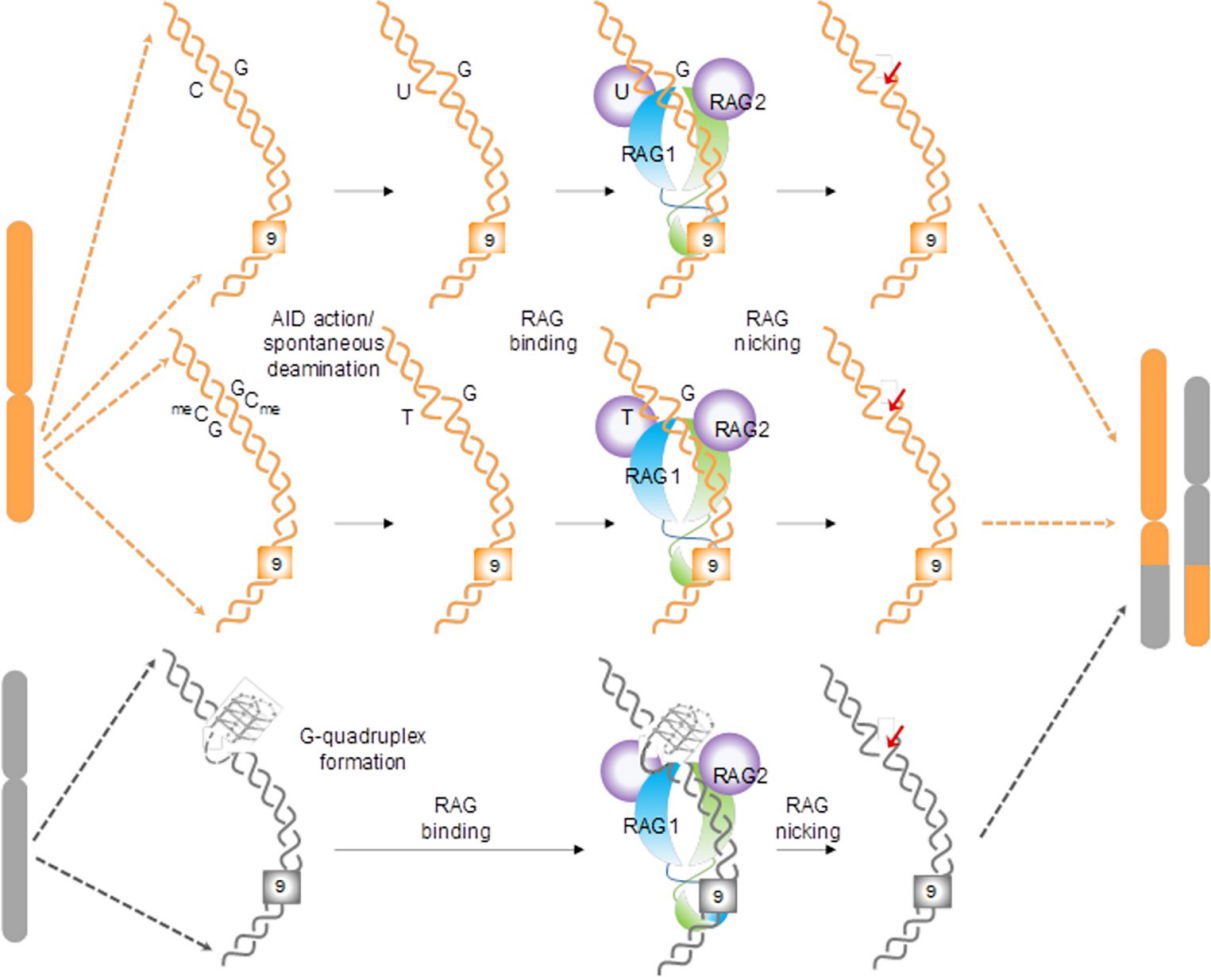
Model for plausible mechanism of RAG-induced chromosomal translocation when CpG sites or cytosines and cryptic nonamers are present adjacent to a breakpoint. Spontaneous or AID mediated deamination/mutations can lead to transient single-strandedness in the genome resulting in U/G or T/G mismatch (shown in orange). Importantly, our results suggest that when such mismatches are present in the proximity of a cryptic nonamer sequence, RAGs can bind and induce a nick at the mismatch leading to a DNA double-strand break, eventually culminating in chromosomal translocations, when a partner chromosome (shown in grey) is present at a close vicinity. This model adds to previous hypothesis that non-B DNA structures like G-quadruplexes, when in proximity to a nonamer can be susceptible to RAG nicking at the non-B DNA structure, which could initiate a translocation event.

Based on the above results in conjunction with previous studies [38,48], we propose a novel mechanism of chromosomal rearrangements which is dependent on CpGs and nonamers and mediated by both AID and RAGs. This may help explain the mechanism of several unreported chromosomal translocations in healthy lymphoid cells and cancer cells associated with lymphoid system (Fig 12).

## Discussion

The analysis of breakpoint junctions from patients with lymphoid malignancies uncovered several examples of translocation breakpoint regions with nearby cryptic nonamers and CpGs. The observation that the stretch of adenines at the 3^rd^, 4^th^, 5^th^ and 6^th^ positions are conserved in 70% of the cases, suggests that even though cryptic nonamers allow binding of RAG1, a certain level of sequence conservation is important for signature sequence recognition. Our analysis also suggests that there is a high chance of DNA breakage in the genome when the distance between cryptic nonamer and CpG is <80 bp, with even higher possibility if the distance is less than 50 bp. However, when whole genome was analyzed for random occurrence of a CpG close to a cryptic nonamer in regions not known to break, the distance between CpGs and cryptic nonamer was 3–5 folds higher (300–500 bp).

Importantly, almost 93% of the chromosomal translocations in lymphoid cancers had breakpoint regions adjacent to cryptic nonamers, of which 73% had CpGs in close proximity to the breakpoints. Interestingly, only 2% of total lymphoid cancer patient breakpoints, occurred in the promoter of genes, although promoters are known to be enriched for CpG sites. Importantly, of that only ~49% possessed CpG and cryptic nonamer in close vicinity, therefore, it is likely that CpG-nonamer mediated RAG cleavage may not explain all the breakpoints seen in promoter region. There could be additional mechanism(s) responsible for generation of chromosomal translocation associated with promoter regions.

Whole genome analysis for random occurrence of cryptic nonamers revealed that density of cryptic nonamers were significantly higher in euchromatin) with breaks as compared to regions with no breaks. The 100 bp window with breaks had an average of 13 nonamers, 2 CpGs and 8 breaks while it was only 1 nonamer and 1 CpG when there was no break (chi square statistic = 94.43 and p-value = 3.1 e-21). This indicates that when multiple CpGs and nonamers are present within a distance of 100 bp, number of breakpoints seen is significantly higher. In contrast, unbroken regions of the genome have fewer numbers of CpGs and nonamers present in close proximity.

When we compared distribution of breakpoints and CpG-cryptic nonamer in euchromatin and heterochromatin regions, we observed that propensity of breakpoint occurrence remained comparable (60–70%) for both the cases. Interestingly, CpG-cryptic nonamer clustering was significantly higher (p-value = 0.02) in euchromatin (74%), compared to heterochromatin region (46%).

Comparison of CpG-cryptic nonamer clustering in lymphoid and nonlymphoid cancers revealed that there was significant difference (8-fold). Further, the occurrence of CpGs was almost double in lymphoid cancer (1.3/ region in lymphoid, 0.6/ region in nonlymphoid), while the occurrence of cryptic nonamer did not differ significantly. This suggests that the proposed AID and RAG mediated translocation mechanism holds greater value for lymphoid translocation.

Biochemical experiments showed that RAG proteins could bind to DNA harboring such nonamers and when a mismatch is present at adjacent CpG, it can induce nick at single to double-strand DNA transition. Although the cleavage was precise in many instances, it was also seen 2–3 nt away from the mismatches. However, there were also examples of fragile regions where RAG nicking was weak even when a mismatch at CpG and cryptic nonamers were present. These results suggest that even if cryptic nonamer/s when present adjacent to a CpG can facilitate the RAG mediated cleavage, the efficiency and precision varied significantly and was dependent on the sequence of cryptic nonamer and the distance from the CpGs. Further, introduction of mutation in CAC sequence (cryptic heptamer), cryptic nonamer 1 and 2 in the case of BCL1 MTCIV revealed that, presence of a T/G mismatch and a nonamer was sufficient for RAG mediated cleavage. Presence of the CAC did not show any impact on RAG cleavage efficiency. We observed that the binding affinity of RAGs to cryptic nonamer of heteroduplex DNA was by virtue of nonamer binding domain of RAG1 and not by the central domain.

Reconstitution assay confirmed our hypothesis as RAGs were able to cleave at AID induced mismatches at AID-hotspot motifs (WRC/GYW) or at CpGs when present near cryptic nonamers both *in vitro* and *in vivo*. Importantly, in most of the instances the cleavage sites observed by us overlapped with previously reported patient breakpoint regions indicating that we are able to recapitulate several features of patient gene rearrangement in our study. Chromatin immunoprecipitation of RAG1 indicated binding of RAGs at cryptic nonamer rich fragile regions. Thus, our results, in conjunction with other studies [38,48], suggest that when cryptic nonamers are present near CpGs, RAG can bind and introduce cleavage at deaminated CpG or cytosines, resulting in the generation of DSBs, that may act as initiating event for chromosomal translocations (Fig 12).

An earlier study had shown clustering of patient breakpoints around CpG sites in the genome [48]. The ability of RAGs to cleave at a single nucleotide mismatch alone was either undetectable or very weak [38,48]. This indicates that the probability of RAGs to cleave at such mismatches, and hence cause chromosomal translocation could be minimal when CpGs are present alone. Our findings suggest presence of a nonamer adjacent to the CpG could enhance RAG activity at a mismatch by several folds. Consistent to this, we observed that mutation or alteration of cryptic nonamers abrogated the efficiency of RAG cleavage significantly. Thus, presence of cryptic nonamers could serve as an additional regulatory motif in determining the incidence of RAG mediated breaks.

Mismatches in the genome could be generated in an AID dependent or independent manner. Spontaneous deamination of cytosines or methylated cytosines present at CpGs may lead to generation of U/G and T/G mismatches, respectively. These reactions are known to occur at a high frequency with the rate constants of 5.8 x 10^−13^ s^-1^ and 2.6 x 10^−13^ s^-1^ for 5-methylcytosine and cytosine in double-stranded DNA, respectively [67]. Constitutive AID activation in malignant cells further raises the potential for inducing mismatches in the genome [44,68]. Our study also revealed that U/G and T/G mismatches on a heteroduplex DNA was crucial for targeted cleavage by RAGs. When such mismatches were absent, RAG cleavage was severely compromised even when a cryptic nonamer or a canonical nonamer was present.

Recent studies have characterized the genome-wide spectrum of translocations that arise from a single double-stranded break and have earmarked several specific loci to have an intrinsic predisposition for frequent chromosomal rearrangements [54]. Our analyses of AID dependent and independent hotspots for the presence of cryptic nonamers have uncovered several cryptic nonamers at the region of chromosomal rearrangements (S1 Table). This further suggests that RAG can indeed act at the mismatches generated due to deamination either by AID or spontaneously, resulting in breaks which could lead to chromosomal translocations.

Although AID and RAGs are expressed in non-overlapping stages during B cell development, previous studies do suggest an overlapping window of AID and RAG expression [66,69–71]. It could either be due to delayed ceasing of RAG expression or its re-induction during receptor editing [72]. This is consistent to our hypothesis that the mismatches generated close to a nonamer can become a preferred target for RAGs.

In case of RSS, distance between heptamer and nonamer is either 12 or 23 nt. DNA bending protein, HMGB1 helps in DNA bending and synapsis during V(D)J recombination within the cells. However, in the case of fragile regions, RAG binding was observed when the distance between cryptic nonamers and CpGs was upto 80 bp, although no synapsis between sequences from partnering chromosomes were observed. However, in cases of fragile regions where low efficiency RAG nicking at mismatches was observed, distance was variable, although generally <80 bp, indicating possibility of DNA bending.

The lower incidence of malignancies due to illegitimate action of RAGs could be due to the lower efficiency of RAG nicking observed at such fragile sites. Besides, status of the chromatin and cell cycles stages may act as additional regulatory mechanisms. Although nicking could be observed at MnCl_2_ concentration as low as 100 μM, this implies that the local ion concentrations may also act as an additional regulatory mechanism.

Although RAGs may be the primary cause of breakage at fragile regions when cryptic nonamers are present adjacent to CpGs, there are number of examples of breakpoints at CpGs where no cryptic nonamers were present within 80 bp. It is likely that failed base excision repair or mismatch repair might have induced the first break in such cases [63,64]. Our results suggest that even in such cases, RAGs may induce a second nick to generate a DSB. Alternatively, replication or transcription across the fragile region may also lead to generation of a DSB [73].

Thus, our study sheds light on a novel mechanism of RAG induced chromosomal translocation outside the antigen receptor loci. *In silico* analysis revealed that several chromosomal translocation breakpoints in various patients are clustered around cryptic nonamers and CpG sites in close vicinity. We demonstrate that the presence of such nonamers can indeed direct RAG binding and cleavage to an adjacent mismatch generated due to spontaneous or AID mediated deamination of a methylated cytosine which may lead to malignancies in lymphoid cells in certain instances.

## Materials and methods

### Enzymes, chemicals, and reagents

Chemicals and reagents used in the study were obtained from Millipore-Sigma, (USA), and SRL (India). Bacterial culture media was from HiMedia (India). Restriction enzymes and DNA modifying enzymes were purchased from New England Biolabs (USA). Culture media were from Sera Laboratory International Limited (UK) and Lonza (Switzerland). Fetal bovine serum and PenStrep were from GIBCO (USA). Antibodies were from Santa Cruz Biotechnology (USA). γP^32^-ATP was purchased from BRIT (India).

### Mammalian cell culture

Human embryonic kidney epithelial cell line expressing Simian Virus 40 large tumour (T) antigen (293T) was grown in DMEM high glucose with L-glutamine containing 10% FBS. Pre-B cell lines, Nalm6, REH and T-cell leukemic cell line, CEM were grown in RPMI medium containing 10% FBS supplemented with 100 μg/ml Penicillin G and streptomycin. Cells were incubated at 37°C in a humidified atmosphere containing 5% CO_2_.

### Oligomers

Oligomers used in the study were synthesized from Sigma-Aldrich (India) and Juniper Life Sciences, (India) and sequences are listed in S2 Table. When required, the oligomers were purified using 10–15% denaturing PAGE. Radiolabeled duplex DNA was prepared by annealing labeled strand with 5-fold excess of its complementary strand in 100 mM NaCl and 1 mM EDTA in a boiling water bath, followed by gradual cooling, as described earlier [74,75]. The oligomers mimicking patient breakpoints, wherein cryptic nonamers are present adjacent to CpGs, were chemically synthesized. T/G mismatch was introduced at the CpG site to mimic the physiological scenario of deamination. Substrates were synthesized corresponding to 5 genes, *BCL1 MTC*, *BCL2 ICR*, *BCL2 MBR*, *E2A*, *FGFR1*, and *KMT2A* along with other control oligomers as detailed below.

### 5′ end-labeling of oligomeric DNA and annealing

5′-end labelling of oligomeric DNA was carried out using T4 polynucleotide kinase in a buffer containing 20 mM Tris-acetate (pH 7.9), 10 mM magnesium acetate, 50 mM potassium acetate, 1 mM dithiothreitol (DTT), and [γ-P^32^] ATP at 37°C for 1 h. The labelled substrates were purified using a Sephadex G-25 size exclusion column as described [29,35] and stored at −20°C until further use.

Klenow ladder was prepared by digesting 5’ end labelled MS3 oligomer (Poly T, 35 nt) with Klenow polymerase in buffer (10 mM Tris-HCl [pH 8], 10 mM MgCl_2_, 150 mM NaCl, 1 mM DTT) at 37°C for 1 h. 0.3 volume of reaction was removed at 15 min, 30 min and 1 h timepoint and terminated by adding equal amount of formamide containing dye. All 3 fractions were then merged, diluted, and loaded alongside 15% denaturing PAGE to correlate RAG cleavage products.

Substrate containing the 12RSS sequence was prepared by annealing γ-^32^P-labelled AKN1 oligomer with unlabelled AKN2 in a ratio of 1:5 in the presence of 100 mM NaCl and 1 mM EDTA [29,35]. Substrate with 23RSS was prepared by annealing 5′-labelled AKN3 to AKN4 as described before. C6 bubble substrate was prepared by annealing 5’ end labelled AKN46 with cold AKN20. Control substrate (C1) harboring U/G mismatch was prepared by annealing 5’ end labelled AKN99 with AKN102. Control substrate C2 (30 bp) was prepared by annealing 5’ end labelled MN37 with MN38. Control DNA, C3, containing random sequence, was prepared by annealing 5’ end labelled VK7 with VK8. Control DNA, C4 was made by annealing RT17 and MN89. Substrates UI, UII and UIII were designed to harbor U/G mismatch and nonamer separated by 6, 12 and 23 bp distance, respectively. These were prepared by annealing 5’ end labelled NM20, NM22 and NM24, respectively with their complementary sequences NM21, NM23 and NM25. Sequences derived from BCL1 MTC namely MTCI, MTCII, MTCIII and MTCIV were prepared by annealing 5’ end labelled NM50 with NM51, 5’ end labelled NM88 with NM89, 5’ end labelled NM90 with NM91 and 5’ end labelled NM92 with NM93, respectively. Substrates derived from BCL2 ICR namely ICRI and ICRII was prepared by annealing 5’ end labelled NM96 with NM97 and 5’ end labelled AP62 with AP63, respectively. MBRI was derived from BCL2 MBR and prepared by annealing 5’ end labelled AP64 with cold AP65. Substrates derived from KMT2A namely, KI, KII and KIII were prepared by annealing 5’ end labelled AP66 with AP67, 5’ end labelled HK2 with HK1 and 5’ end labelled HK4 with HK3, respectively. Substrates derived from E2A namely EI and EII were prepared by annealing 5’ end labelled AP68 with AP69 and 5’ end labelled AP70 with AP71. Substrates designed based on FGFR1 namely, FI and FII were prepared by annealing 5’ end labelled HK5 with HK6 and 5’ end labelled HK7 with HK8, respectively.

MTCIV derived control substrates with mutation only at CAC or cryptic nonamer were prepared as follows. Cryptic nonamer 2 mutated substrates AP152/153 was prepared by annealing 5’ end labelled AP152 with AP153. Cryptic nonamer 1 mutated substrate AP146/147 was prepared by annealing 5’ end labelled AP146 with AP147. AP127/128 (T/G alone) have both cryptic nonamers mutated whereas AP125/126 (nonamer alone) have CpG adjacent to cryptic nonamer and mutated CAC. CAC mutated substrate AP156/157 was prepared by annealing 5’ end labelled AP156 with AP157. CAC and cryptic nonamer 1 mutated substrate AP148/149 was prepared by annealing 5’ end labelled AP148 with AP149. CAC and cryptic nonamer 2 mutated substrates AP158/159 was prepared by annealing 5’ end labelled AP158 with AP159. Only T/G harboring substrate (CAC and both cryptic nonamers mutated) AP150/151 was prepared by annealing 5’ end labelled AP150 with AP151.

DNA substrates containing 3 random regions devoid of patient breakpoints, but containing CpGs and cryptic nonamers were designed and annealed as follows. Region 1 (ERP44) derived ‘CpG-9mer’ or ‘T/G-9mer’ were prepared by annealing 5’ end labelled AP137 with AP139 and 5’ end labelled AP138 with AP139. Region 2 (CNTN5) derived substrates, CpG-9mer’ and ‘T/G-9mer’ were prepared by annealing 5’ end labelled AP140 with AP142 and 5’ end labelled AP141 with AP142. Furthermore, region 3 (chr7:99995000–99995100) derived substrates CpG-9mer’ and ‘T/G-9mer’ were prepared by annealing 5’ end labelled AP143 with AP145 and 5’ end labelled AP144 with AP145, respectively.

DNA substrates containing random regions (2 AT rich, 2 GC rich) devoid of patient breakpoints, but containing CpGs and cryptic nonamers were prepared as follows. AT rich region 1 (chr4:187419800–187419900, GC 31.8%) derived ‘CpG-9mer’ and ‘T/G-9mer’ were prepared by annealing 5’ end labelled AP166 with AP168 and 5’ end labelled AP167 with AP168. AT rich region 2 (chr12:111925800–111925900, GC 36.5%) derived ‘CpG-9mer’ and ‘T/G-9mer’ were prepared by annealing 5’ end labelled AP169 with AP171 and 5’ end labelled AP170 with AP171. GC rich region 1 (DYNC2H1, GC 63.6%) derived ‘CpG-9mer’ and ‘T/G-9mer’ were prepared by annealing 5’ end labelled AP172 with AP174 and 5’ end labelled AP173 with AP174. GC rich region 2 (NDOR1, GC 67.3%) derived ‘CpG-9mer’ and ‘T/G-9mer’ were prepared by annealing 5’ end labelled AP175 with AP177 and 5’ end labelled AP176 with AP177.

UII derived substrates ‘nick’ and ‘gap’ were designed to mimic base excision and mismatch repair intermediates. In these cases, 5’ end labelled NM23 was annealed with AP134 and AP136 (U/G mismatch is replaced by nick), or AP134 and AP135 (single nucleotide gap in place of U/G mismatch), U/G mismatch from UII substrates were removed to generate double stranded DNA (dsDNA) by annealing 5’ end labelled AP133 with NM23. Sequences for all the oligomers used in the study are presented in S2 Table.

### Plasmid constructs

pAP15 was constructed by cloning *KMT2A* fragile region. A 574 bp region (118481959–118482532 of Accession No. NC_000011.10) from *KMT2A* fragile region was amplified from REH genomic DNA using primers, AP60 and AP61. The PCR product was TA cloned in pTZ57R/T vector to generate pAP15 plasmid construct and identity of the clone was confirmed by restriction digestion followed by DNA sequencing.

GST tagged human core RAGs expression constructs were from Dr. M. Lieber (USA); MBP tagged cRAGs and HMGB1 constructs were from Dr. P. Swanson (USA); pRS3 construct encoding RAG1-CD was from Dr. K.K. Rodgers (USA); RAG1-NBD construct and pEBGRAG1 D708A were from Dr. D.G. Schatz (USA); GST-AID expression construct pGEX-5X-3p was from Dr. A. Martin (Canada); pCMV-wtAID-3x FLAG was from Dr. T. Honjo (Japan). NBD deleted RAG1 containing amino acid 402–1040 (pAKN2) was generated in the lab [37].

Plasmid containing mutant of AID deaminase domain, putative-pRetroX-TRE3G-hAID-H56R,E58Q-puro(AI) was a kind gift from Dr. D.G. Schatz (USA). In order to overexpress mutant of deaminase domain mutant of AID in *E*. *coli*, the DNA fragment containing mutated AID gene was released by BamHI-NotI digestion and cloned in pGEX-5X-3p to generate the pAP28 construct.

### Chromosomal translocation breakpoint mapping and analysis for the presence of cryptic nonamers and CpGs in close proximity

Human translocation breakpoint data were retrieved from specialized databases like TICdb (www.unav.es/genetica/TICdb/) (TICdb: a collection of gene-mapped translocation breakpoints in cancer) [76] and COSMIC (http://www.sanger.ac.uk/genetics/CGP/cosmic/) [51,52] and published literature for the genes from the most common chromosomal rearrangements in human lymphoid malignancies [48,53,54]. A total of 3760 breakpoint pairs were downloaded and used for analysis. Fusion sequences from TICdb were mapped to the hg19 human reference genome using the tool blast: BLAT-like Alignment Tool (v. 36x5) from the Kent utilities distribution. Nucleotide sequences flanking breakpoint locations were retrieved using the BEDTools suite and the breakpoint locations were plotted on to the genes using inhouse python scripts. Breakpoint locations from the COSMIC database available via the tab separated CosmicBreakpointsExport.tsv file was obtained and the corresponding genes and flanking DNA sequences were retrieved using the UCSC genome browser and the BEDTools suite, respectively.

We distributed the genome in windows of 100 bps and calculated the number of breaks, cryptic nonamers and CGs. To determine the contribution of CG sites and cryptic nonamers in the occurrence of breaks, we considered only those regions where there were at least 3 breaks and divided the data into two categories; windows with CGs and those without CpGs. We also categorized the regions into broken and unbroken regions to perform the same comparison. There were 30 regions with at least 3 breaks (as a cut off) which was considered as a normalization factor (Fig 2A).

Inhouse Python and Shell scripts were used to mark the presence of CpGs and cryptic nonamers (upto 4 mismatches) adjacent to translocation hot spots for analysis in 1420 genes. Nucleotide variation at every position of the cryptic nonamers was also calculated using in-house shell scripts. For a consolidated representation of the data, Circos, a tool for circular visualization of genomic information was used. A whole genome Circos was plotted comparing the number breakpoints in the outermost track to the number of nonamers in the middle track and the number of CpGs in the inner most track, each over a window of 1 Kb. Additionally, 26 different genes involved in human lymphoid/hematopoietic cancers are shown in the outer ring as a representation. Each gene has been assigned a specific color. The three connectors originating from each gene terminate at breakpoints, cryptic nonamers and CpGs, respectively implying the presence of either of them in the gene. The width of each colored ribbon depicts the percentage of breakpoints and frequency of cryptic nonamer or CpGs in the particular gene. The total number of patients and breakpoints were calculated and the number of breakpoints adjacent to cryptic nonamer alone or both cryptic nonamer and CpGs were plotted using a pie chart.

To perform comparative analysis of genome characteristics euchromatin vs heterochromatin regions were considered. Further, frequency occurrence of translocation breakpoints was evaluated in the promoter of the genes. The locations for promoters were obtained from the table browser utility of the UCSC genome browser. The euchromatin and heterochromatin regions were obtained from the Giemsa staining information available in the table browser utility of the UCSC genome browser. However, certain areas of the genome, like repeat regions, centromeric, telomeric, nucleolar organization regions, etc., were not considered since information related to these regions in the genome was not publicly available. The analysis of the breakpoint regions and unbroken regions overlapping with the above genomic regions were performed using BEDTools suite.

The distance (number of nucleotides) between CpGs and cryptic nonamers in the vicinity of breakpoints and at other chromosomal locations without breakpoints were calculated using in-house Python scripts. Chi-square statistics were applied to study the significance. The distance between CpG and cryptic nonamer in the vicinity of a cryptic nonamer was considered to be significantly lesser than the distance in the absence of breakpoint if the p-value obtained from Chi-square statistics was less than 0.001. The Bar graphs depicting the same were plotted in Microsoft Excel.

The gene body regions were obtained from the UCSC genome browser based on the genic boundaries of the hg19 gene coordinates. 100 base pair windows with more than 2 breaks were intersected with the gene coordinates to obtain broken windows belonging to genic regions using the BEDTools suite. Unbroken windows (without any breaks) were also obtained from the corresponding genes. In order to select the unbroken windows, we considered those that had genomic characteristics (AT/GC %) similar to that of the broken regions. There were 39 broken windows specific to the genic regions, whereas 24293 unbroken windows. From the unbroken windows, we selected 39 random unbroken regions of the similar GC content as that of the broken windows. Further, we performed 1000 random iterations using in-house shell scripts and analyzed 39 unbroken windows in every iteration.

The nucleotide sequences of the 100 base pair windows were extracted using the BEDTools suite. Occurrence of cryptic nonamers and CpGs were evaluated from window, along with the percentage of windows containing the cryptic nonamers and CpG, using in-house shell and python scripts. The pie charts and histogram were generated using Microsoft Excel

To check whether cryptic nonamers were present in RAG binding sites, we downloaded the wiggle files from a human thymocyte, RAG1-ChIP experiment (GSM1701805), converted them to the bedGraph format using the bigwigToBedGraph operation from Kent Utilities. The breakpoint locations in bed format and the peaks obtained above were visualized and plotted using the IGV browser. The nucleotide sequences of genomic regions, where an abundance of breakpoints was seen coincided with RAG1 binding peaks, were extracted and checked for the presence of cryptic nonamers.

A position weight matrix, based on the frequency of occurrence of nucleotides, was generated using all the RAG1 binding sites in the vicinity of breakpoints. A PWM logo was generated using the matrix using the online tool WebLogo [77].

All the comparisons for broken vs unbroken regions and regions with and without CGs were drawn using Chi-square statistics and the results were considered to be significant if p-value was less than 0.05.

### Protein expression and purification

The mammalian expression constructs harboring core RAG1 (cRAG1, amino acids 384–1040), core RAG2 (cRAG2, amino acids 1–383) each fused with either N-terminal GST tag or MBP tag were used for expression [31,37,58]. pAKN2 containing NBD deleted RAG1 was overexpressed with cRAG2 in HEK293T cell line and purified [37]. Nuclease dead RAG1 mutant construct pEBGRAG1 D708A and GST cRAG2 was overexpressed and purified to obtain nuclease dead mutant cRAGs [78]. Purity of the RAG proteins was assessed using 8% SDS-PAGE and identity was confirmed by western blotting.

The central domain of RAG1 (amino acids 528–760) fused with MBP tag encoded by plasmid pRS3 was expressed in *Escherichia coli* and purified as described previously [58,60]. NBD of RAG1 in pET28a was a kind gift from D.G. Schatz [79]. The protein was expressed and purified from *E*. *coli* BL21 [80]. In brief, the culture was induced with 1 mM IPTG at an OD of 0.5, allowed to grow at 16°C for 16 h and lysed by sonication in extraction buffer (20 mM sodium phosphate buffer (pH 7.2), 500 mM NaCl, 2 mM β-mercaptoethanol, 1% Triton X 100, 10% glycerol) with 10 mg/ml lysozyme [58,80]. The soluble fraction of the cell lysate was then purified using Ni-NTA affinity chromatography as per the manufacturers protocol (Invitrogen). HMGB1 protein was expressed in *E*.*coli* BL21 and purified using Ni-NTA affinity chromatography by virtue of histidine tag [81].

For AID purification, pGEX-5X-3p expression construct and mutant AID construct, pAP28 was transformed in BL21 (DE3) *E*. *coli*. Single colony was selected and cultured further for protein purification. Prior to induction, cells were grown up to 0.8 O.D., and protein expression was induced using IPTG (1 mM) for 16 h at 16°C. After induction, cells were pelleted down and lysed in buffer containing 1X PBS, 1% Triton X-100, 1 mM PMSF. Glutathione-sepharose column (GE, USA/Sweden), was used for purifying the protein [82]. Purity of the GST-AID was assessed using 8–10% SDS-PAGE and identity was confirmed by western blotting.

### AID deamination assay

Radiolabeled DNA substrate harboring AID hotspots, DGYW (AP73) and WRC motifs (ASM4) was incubated with purified fractions of AID and deaminase domain mutant of AID (for 60 min at 37°C) in a buffer containing 50 mM Tris (pH 7.5), 100 mM NaCl, and 20 μM MgCl_2_ in a volume of 10 μl. The protein was then heat inactivated at 75°C (15 min). 1 U of uracil DNA glycosylase (NEB, USA), and RNase Inhibitor were added to this in 1X base-excision buffer (20 mM Tris (pH 8), 1 mM DTT and 1 mM EDTA), followed by incubation at 37°C (for 60 min). Finally, NaOH (10 mM) was added to stop the reaction, and the samples were heated to 95°C for 8 min to cleave the abasic site generated by removal of uracil. The reaction products were resolved on a 18% denaturing PAGE and image was acquired using phosphorImager, FLA9000 (Fuji, Japan).

### Electrophoretic mobility shift assay (EMSA)

EMSA was carried out as described previously [38,83]. In brief, radiolabeled DNA substrates were incubated with appropriate proteins in a buffer (1X) containing 22.5 mM MOPS-KOH (pH 7.0), 20% DMSO, 2.2 mM DTT, 50 mM potassium glutamate, 2% (v/v) glycerol and BSA (100 ng/ml) for 2 h at 25°C. In the control, RAG reaction buffer alone was used. The DNA-protein complexes were then resolved on 5% or 6% native polyacrylamide gels. The gels were dried, and bands were visualized by FLA9000 phosphorImager (Fuji, Japan). EMSA using NBD was performed as described earlier [79,80]. Each experiment was repeated a minimum of two times with complete agreement.

### RAG cleavage assay

RAG cleavage was performed as described earlier in a buffer containing 25 mM MOPS (pH 7.0), 30 mM KCl, 30 mM potassium glutamate, 5 mM MgCl_2_ and 5 mM MnCl_2_ [31,37,38]. RAG proteins were added to radiolabeled oligomers corresponding to 12RSS, patient breakpoints or heteroduplex DNA harboring T/G or U/G or mismatch and cryptic or canonical nonamers and incubated at 37°C for 1 h. Reactions were terminated by addition of loading dye containing formamide, heated for 10 min at 95°C and electrophoresed on 15% denaturing polyacrylamide gels. To assess the requirement of manganese ion in RAG nicking at T/G-nonamer substrates, assay was also performed using increasing concentration of MnCl_2_ (100, 200, 300, 400, 500 μM). Each experiment described in the present study was done a minimum of three independent times with complete agreement.

### Competition assay for determining specificity of RAG binding

The specificity of binding of RAGs to regions of the genome wherein patient breakpoints are clustered around cryptic nonamers was confirmed by performing a competition assay with specific or non-specific competitor substrates as described [80]. EMSA was performed by incubating constant amount of labeled DNA representing *BCL1* MTC and RAGs with increasing concentration of unlabeled specific or nonspecific DNA (DNA substrate, C3). The concentrations used were 8 nM labeled substrate and 0, 4, 6, 8, 10 and 12 nM for nonspecific competitor and 0, 2, 4, 8, 16 and 32 nM for specific substrates [58]. Following RAG binding, the products were resolved on a 5% native polyacrylamide gel in either case.

### Assay for activity of preformed complexes to carry out coupled cleavage

The ability of preformed complexes to carry out coupled cleavage was assayed in reactions containing RAGs, radiolabeled heteroduplex DNA and cold 12 or 23RSS, in RAG cleavage buffer containing 5 mM MnCl_2_ (37°C for 1 h) in presence or absence of HMGB1 as described before [84–86]. RAG cleavage reactions were terminated, and the products were analyzed on a denaturing PAGE.

### Reconstitution of RAG induced breaks at chromosomal fragile regions

Plasmid DNA (4 μg) containing fragile regions KMT2A (pAP15), was denatured using alkali (0.2 N NaOH, 0.1 mM EDTA for 5 min at RT) and precipitated using 0.8 M ammonium acetate and chilled ethanol. Alternatively, plasmid DNA was heat denatured at 95°C for 10 mins, followed by snap chilling.

300 ng of denatured plasmid DNA (either by alkali or heat) was treated with 450 ng of AID protein at 37°C for 2 h in a buffer containing 50 mM Tris-HCl (pH 7.5), 100 mM NaCl, and 2 μM MgCl_2_. The reaction was then kept in boiling water for 10 min to denature the plasmid DNA, and later allowed to cool slowly for renaturation of the DNA. The annealed DNA was used for RAG cleavage assay with GST cRAGs in buffer containing 25 mM MOPS (pH 7.0), 30 mM KCl, 30 mM potassium glutamate, 5 mM MgCl_2_ and 5 mM MnCl_2_, reactions were incubated for 90 min at 37°C. Similarly, reactions were carried out with deaminase domain mutant of AID or nuclease dead RAGs. Products were purified using phenol: chloroform (1:1) method and precipitated using ethanol precipitation in presence of glycogen and resuspended in nuclease free water. 100 ng purified DNA was used for primer extension assay, with 1 U Vent exo polymerase in 1X Thermo polymerase buffer, 4 mM MgSO_4_, 200 μM dNTPs and 0.1 nM 5’ end labelled primer AP72 or AP73 (radioactively labelled) [87]. Primer extension was performed using the following conditions: 95°C for 5 min, (95°C for 1 min, 56°C for 45 sec, 72°C for 45 sec) for 25 cycles and the final extension at 72°C for 5 min. Following primer extension, reaction products were mixed with formamide dye, and were resolved on 8% denaturing polyacrylamide gels. The gels were dried, and the signal was detected using phosphorImager (Fuji, Japan).

### Ligation-mediated PCR to detect DSBs

Ligation-mediated PCR technique was carried out to assess for DNA double-strand breaks [12,88–90]. To do this, Nalm6 and CEM cells were transfected with the episome, pAP15 containing KMT2A fragile region, along with AID overexpression vector (pCMV-wtAID-3x FLAG). Besides, CEM cells were co-transfected with pAP15 and deaminase domain mutant overexpression vector putative-pRetroX-TRE3G-hAID-H56R,E58Q-puro(AI). Transfected cells were treated with 5 μg/ml doxycycline and 0.7 μg/ml puromycin 24 h post transfection for induction and selection respectively. AID overexpression was confirmed by western blotting. Transfection products were extracted 48 h post-transfection by Hirt-harvest method [91,92] and used for linker ligation. SCR58 (25 nt) and SCR59 (15 nt) were annealed to form linker of asymmetric nature which can ligate only in a single orientation. As a positive control, pAP15 digested with ScaI, blunt end cutter was subjected it linker ligation. Resulting ligated samples were purified by phenol/chloroform, ethanol precipitated and used for PCR using linker specific primer (AP116) and gene specific primer (either AP72 or AP73). When required, reamplification was performed using same set of primers. PCR conditions used were: initial denaturation at 95°C for 5 minutes, cycling conditions were 95°C for 30 s; 57°C for 30 s; 72°C for 30 s for 30 cycles. PCR products were gel purified, cloned and sequenced when required.

### Bio-layer interferometry

Bio-layer interferometry (BLI) was performed as described before [93,94]. ForteBio Octet RED 96 (Forte Bio, USA) system was employed for studying the binding of (His)6 tagged RAG1-NBD (analytes) to FGFR1-I and KMT2A-I (ligand) loaded onto the SAX (Forte Bio, USA) sensors. 1X PBS was used as the assay buffer and the study was conducted at 30°C.

Before use, all the sensor tips were hydrated in buffer (10 min). 96-microwell plate filled with 200 μl of buffer, containing proteins, was agitated at 1500 rpm. The program was set up in a sequence of steps, which included establishment of a stable baseline with buffer (1 min), loading of sensors with biotinylated double stranded DNA substrates, 1 μM (6 min). A reference sensor without bound ligand (DNA) was subjected to the same procedure as the sample sensors loaded with ligand and used for subtraction of the background signals. Following immobilization with different biotinylated double stranded DNA substrates (biotinylated 12RSS, FGFR1I and KMT2AI) in independent binding experiments with different concentrations of RAG1 NBD (0.36, 0.91 and 2.22 nM) were carried out, which included baseline (1 min), association (10 min), dissociation (10 min), baseline (8 min) and the KD values were calculated using curve fit (1:1, association: dissociation) model.

### Chromatin immunoprecipitation for RAG1

Chromatin Immunoprecipitation (ChIP) was performed using modified ChIP protocol described previously [95,96]. Nalm6 cells were cultured and about 1*10^7^ cells were for used ChIP studies. Cross-linking was performed using formaldehyde (1%) for 15 min at 22°C, followed by quenching (5 min) using glycine (125 mM). Crosslinked cells were pelleted down and washed twice with ice cold 1X PBS. Pellet was resuspended in 2 ml of lysis buffer (5 mM PIPES [pH 8.0], 85 mM KCl and 0.5% NP-40) supplemented with protease inhibitors and incubated on ice for 10 min, followed by centrifugation at 4°C (10 min). Nuclear pellet was then resuspended in 200 μL of MNase digestion buffer (50 mM Tris-HCl (pH 8.0), 5 mM CaCl_2_, 0.2% NP-40) to which 2 U of MNase (NEB, USA) was added and incubated (37°C for 10 min) with slow agitation. Reaction was terminated with the addition of 50 mM EDTA and the chromatin obtained was further sheared through sonication (10 cycles with 30 sec ON, 45 sec OFF pulse; Bioruptor, Diagenode, Belgium). This resulted invariably in chromatin of 100–500 bp in size.

Chromatin concentration was measured using Nanodrop 2000, ThermoFisher (Massachusetts, USA). 50 μg of chromatin was incubated overnight with 2 μg anti-RAG1 antibody (Santa Cruz, SC-363) and secondary antibody control, anti-rabbit IgG (Santa Cruz, SC-2025). 5% chromatin was kept as input. The chromatin-Ab complexes were then mixed with Protein A/G beads and incubated for 2 h, at 4°C on an end-to-end rotor. Beads were pelleted down (2000 RPM, 4°C) washed twice to remove non-specific binding in wash buffer 1 (50 mM Tris-HCl [pH 8.0], 1 mM EDTA, 0.1% Triton X-100, 0.1% Na-deoxycholate and 0.1% SDS), four times with wash buffer 2 (50 mM Tris-HCl [pH 8.0], 1 mM EDTA, 0.1% Triton X-100, 0.1% Na-deoxycholate, 0.1% SDS and 350 mM NaCl) and once with TE (10 mM Tris-HCl [pH 8.0], 1 mM EDTA). For each wash, beads were kept on end-to-end rotator for 20 min at 4°C, followed with centrifugation (4°C, 2000 RPM). The IP-ed complexes were eluted using 150 μl of elution buffer (50 mM Tris-HCl [pH 8.0], 1 mM EDTA, 1% SDS, 0.2 M NaCl) at 65°C for 10 min. To the eluate, RNase A (20 μg) was added and incubated for 1 h (at 37°C), followed by Proteinase K treatment (40 μg), for 1 h (at 65°C). Reverse crosslinking was performed at 65°C overnight. DNA was purified using phenol:chloroform followed by ethanol preparation in presence of glycogen. Samples were resuspended in 30 μl of nuclease free water and used for PCR.

Input samples were processed similar to experimental from the elution step. Analysis was done using semi-quantitative as well as qPCR (BioRad CFX96, USA) for all the regions of interest. Fold enrichment with respect to IgG was calculated using 2^-ΔΔCt^ method and is represented as a bar graph. Statistical significance was calculated using Student’s t-test with two tailed distribution using GraphPad Prism 5 (Version 5.01). Experiment was performed thrice with complete agreement. ChIP-PCR primers used in this study are listed in S2 Table.

SV50 and SV51 primers were used to amplify *VEGF* random region devoid of patient breakpoints (198 bp). SV68 and SV69 were used to amplify *MyoD* random region devoid of patient breakpoints (107 bp). SV52 and SV53 were used to amplify *Myc* random region devoid of patient breakpoints (293 bp). RR17 and RR18 were used to amplify *ABL1* (113 bp). AP88 and AP89 were used to amplify *BCL1 MTC* (203 bp). RR15 and RR16 were used to amplify *BCL2 MBR* (116 bp). RR13 and RR14 were used to amplify *BCL2 ICR* (352 bp). RR11 and RR12 were used to amplify *E2A* (293 bp). RR9 and RR10 were used to amplify *FGFR1* (239 bp). AP72 and AP73 primers were used to amplify *KMT2A* (188 bp). AP90 and AP91 primers were used to amplify *PDGFRA* (111 bp). AP84 and AP85 were used to amplify positive control *IgHV* (IGHV3-74, 237 bp). AP86 and AP87 were used to amplify positive control *IgHD* (IGHD2-21, 260 bp).

## Supporting information

S1 Table Selected examples of chromosomal translocations associated with lymphoid malignancies and its correlation with cryptic nonamers. The table shows detailed information on translocations, genes involved, associated different lymphoid malignancies along with number of CpG sites and cryptic nonamers in the vicinity of breakpoints found in the analysed genes.
(TIFF)

S2 Table List of oligomeric DNA substrates used in the study. (TIFF)

S1 Fig Sequence of selected breakpoint regions associated with lymphoid malignancies. **A-F.** Sequence of breakpoint regions mapped from lymphoid malignancies for the occurrence of chromosomal translocation breakpoints near cryptic nonamers and CpGs of selected genes BCL1 MTC (A), BCL2 ICR (B), BCL2 MBR (C), E2A (D), FGFR1 (E), KMT2A (F). Each breakpoint is represented by an arrowhead adjoining the breakpoint site. Red highlighted sequence indicates CpG sites. Green highlighted sequence indicates cryptic nonamers. Different intensity grey lines on the top of gene sequence corresponds to cryptic nonamers with different levels of conservations with respect to the canonical nonamer. Different colored lines at bottom of gene sequence indicated sequence of DNA substrates used in gel-based assay.
(TIFF)

S2 Fig Evaluation of association between occurrence of CpGs and cryptic nonamers in whole genome. **A.** Pie chart showing comparison of incidence of breakpoint region in promoter and nonpromoter regions of genes. The right panel shows comparison of occurrence of CpGs and nonamer when breakpoints are seen in promoters. **B.** A boxplot showing number of nonamers/kb in regions with breaks as compared to unbroken (control) regions of the human genome. **C.** The pie charts depict percentage of windows with and without CpGs in genic region that are broken (a) and unbroken (b). The percentage of windows with and without CpGs along with cryptic nonamers in broken (c) and unbroken (d) genic regions are also shown. In the case of unbroken region equivalent number of random windows containing genic regions were selected. **D.** comparison of distribution of breakpoints in euchromatin and heterochromatin regions and their correlation with occurrence of CpGs and nonamers. a) Pie chart showing distribution of breakpoint regions in euchromatin and heterochromatin regions. b) Pie chart showing distribution of equivalent length “no-break” random regions in euchromatin and heterochromatin regions. c) Pie chart showing distribution of euchromatic break regions containing CpG and cryptic nonamers. d) Pie chart showing distribution of euchromatic “no-break” regions containing CpG and cryptic nonamers. **E.** Table showing distribution of breakpoints, cryptic nonamers and CpGs in case of lymphoid and nonlymphoid tumor samples. **F**. A Venn diagram showing common genes (1.5%) between lymphoid and nonlymphoid translocations. **G.** Bar graph showing the difference in number of CpGs, cryptic nonamers per 100 bp region, between breakpoints of lymphoid and nonlymphoid translocations from the same genes. **H.** A ChIP seq plot showing the peak intensity of RAG1 binding over a locus of TCRVB gene, along with breakpoints (represented with black arrows) from our studies clustered around the same location. One of the peaks has been highlighted and the sequence has been expanded to show the presence of cryptic nonamers in the sequence.
(TIFF)

S3 Fig Evaluation of purity, identity and activity of cRAGs. **A.** Gel profile showing purified cRAGs. ‘M’ is molecular weight ladder. **B.** Western blot showing confirmation of identity of cRAGs from 293T cells. Identity of the proteins was checked using anti RAG1 and RAG2. **C.** Sequence of 12RSS substrate used for activity assay. **D.** Activity assay of purified cRAGs on 12RSS substrate. DNA was subjected to RAG mediated cleavage and the products were resolved on a 15% denaturing polyacrylamide gel. The cleavage products are indicated with an arrowhead. The fractions that exhibited DNA cleavage activity were used for the further assays.
(TIFF)

S4 Fig Binding and cleavage of RAGs on U/G mismatch substrates. **A.** General design of DNA substrates used in the assays. **B.** Schematic of heteroduplex DNA substrates harboring U/G mismatch and V(D)J nonamer (canonical). Oligomers are designed to harbor U/G mismatch alone (CI), or U/G mismatch and canonical nonamer placed 6 nt (UI), 12 nt (UII), 23 nt (UIII) downstream to mismatch. **C.** Gel profile showing RAG binding of DNA substrates containing U/G mismatch and canonical nonamer. **D, E.** Gel profile showing RAG cleavage assay on DNA substrates containing U/G mismatch and canonical nonamer. For cleavage assay, 15% denaturing gel was used. The cleaved product is marked with an arrowhead. M is molecular weight marker. C4 and CI are controls that correspond to random DNA control and U/G mismatch without nonamer, respectively.
(TIFF)

S5 Fig Schematic showing mutated heteroduplex DNA derived from MTCIV substrates. Corresponding oligomers were annealed to form a double stranded DNA with either T/G mismatch or mutated cryptic nonamer 1, 2 or both. CAC downstream to T/G mismatch is shown in blue. T/G mismatch is shown in orange oval, whereas cryptic nonamer is shown in purple and yellow. Mutated nucleotides to abrogate existing nonamer or CAC are highlighted in pink
(TIFF)

S6 Fig Evaluation of impact of mismatches and cryptic nonamers on RAG cleavage when present on different random regions of the genome. **A.** Schematic showing sequences of random DNA substrates (3 regions) used for the study with mismatches and cryptic nonamers, where no breakpoints are reported. Orange oval indicates T/G mismatch, purple highlight indicates nonamer whereas CpG nearby nonamer is indicated by yellow. Corresponding oligomers were annealed to form a double stranded DNA with either T/G mismatch and cryptic nonamer. **B**. Gel profile showing cleavage by RAGs on DNA substrates derived from regions devoid of breakpoints depicted in panel A. **C.** Quantification showing RAG cleavage on 3 random regions in presence of either a CpG or a mismatch. **D.** Schematic showing sequences of random DNA substrates (2 AT and 2 GC rich) used for the study with mismatches and cryptic nonamers, where no breakpoints are reported. Orange oval indicates T/G mismatch, purple highlight indicates main cryptic nonamer whereas CpG nearby nonamer is indicated by yellow. **E.** Gel profile showing cleavage by RAGs on DNA substrates derived from 2 AT and 2 GC rich regions devoid of breakpoints. ‘PC’ is positive control in which RAG cleavage reaction was performed using labelled 12RSS. ‘M’ is 1 nt Klenow ladder. **F.** Quantification showing RAG cleavage on AT and GC rich regions devoid of breakpoints. Error bar was calculated as mean ± SEM. *p < 0.05, **p < 0.005, ***p <0.0001. ns, not significant; AU is arbitrary unit.
(TIFF)

S7 Fig Binding of RAG1 domains with DNA substrates containing U/G mismatch and canonical nonamer. **A.** Diagrammatic representation of oligomeric DNA substrates used in the study. The oligomeric DNA substrates containing U/G mismatch alone (C1) and those with a canonical nonamer placed 6 (UI), 12 (UII) and 23 (UIII) bp downstream to it are shown. Besides, C6 bubble is also shown. **B.** Native gel profile showing the binding of nonamer binding domain (NBD) of RAG1. **C.** Native gel profile showing the binding of central domain of RAG1. **D.** Binding profile of NBD deleted cRAG1/ cRAG2 complex on DNA harboring U/G mismatch with a canonical nonamer placed 6, 12 and 23 nt away from it or a heteroduplex DNA with cytosine bubble. The bands due to RAG binding are indicated by arrows in panels B-D.
(TIFF)

S8 Fig Chromatin immunoprecipitation studies to evaluate binding of RAG1 to different translocation fragile regions within lymphoid cells. **A.** Procedure used for RAG1 chromatin immunoprecipitation. **B.** Melt curves for each of the 13 genes used in ChIP qPCR analysis. **C.** Representative amplification curves (green represents the control amplification curve and red depicts the experimental regions) for all genes analyzed are presented.
(TIFF)

S9 Fig Table showing binding of RAG1 to different translocation fragile regions within lymphoid cells. Ct values obtained for all 13 genes analyzed using ChIP qPCR are shown.
(TIFF)

S10 Fig Outline of reconstitution assay used for detecting AID/RAGs induced cleavage in fragile regions and identity and activity of purified AID. **A.** Schematic showing summary of steps involved in *in vitro* reconstitution assay. **B.** SDS-PAGE profile showing purified GST-AID. **C.** Western blot for purified GST tagged AID. **D.** Pictorial representation of primer extension assay used for detecting RAG-induced breaks. After the treatment of plasmid with AID and RAGs, DNA strand is amplified in primer extension assay separately using single primer so that RAGs induced single-stranded breaks can be detected as pause sites when resolved on 8% denaturing PAGE. **E.** Oligomeric DNA used in activity assay of purified AID. **F.** Oligomeric substrate-based activity assay for purified AID. Expected cleavage product of 13 bp is indicated with arrow.
(TIFF)

S11 Fig Overexpression, purification and activity check of nuclease dead RAG1 and deaminase domain mutated AID. **A.** Silver-stained gel profile of ΔcRAGs (pEBGRAG1 D708A and GST tagged cRAG2). ΔcRAGs were overexpressed in HEK293T cell line and purified. ΔcRAG1 and cRAG2 bands are indicated using arrows. **B.** Activity assay of purified ΔcRAGs on 12RSS substrate. Lane 1 is positive control in which 12RSS was incubated with cRAGs (wild type). Lane 3–8 are reactions products obtained following incubation of 12RSS with purified fractions of ΔcRAGs. Radiolabelled substrate DNA (12RSS) was loaded in lane 2. **C.** CBB profile of ΔAID in which deaminase domain is mutated (H56R, E58Q). **D.** Oligomeric DNA used for activity assay to test the purified ΔAID and its wild type. DGYW and CpG motifs are highlighted in yellow and green color, respectively. **E.** Comparison of activity of purified ΔAID with that of wild type. Bands of interest are indicated using arrows and blue box.
(TIFF)

S12 Fig Detection of DNA breaks induced at chromosomal fragile region, KMT2A in Nalm6 cells using LM-PCR. A. Schematic of KMT2A breakpoint seen in patents mapped along with LM-PCR breakpoints detected in our assays. Grey downward arrows represent patient breakpoints, while blue upward arrows indicate breakpoints determined following LM-PCR in this study. **B.** Chromatogram of breakpoint junctions obtained following PCR and cloning. Left side represents linker sequence whereas sequence in the right side of the vertical line indicates KMT2A sequence.
(TIFF)

S1 Data Chromosomal translocation breakpoints in patients with lymphoid malignancies and frequency of occurrence of CpGs and cryptic nonamer at or near breakpoint regions. Excel files showing the chromosomal translocation breakpoints, cryptic nonamers and CpGs in patients with lymphoid malignancies and frequency of occurrence of CpGs and cryptic nonamer at or near translocation breakpoint regions. This dataset is related to Fig 1.
(XLSX)

S2 Data Evaluation of occurrence of cryptic nonamer and CpGs in human genome and their correlation with incidence of patient breakpoints. Excel files representing the number of breaks, cryptic nonamers, and CpGs between regions in the human genome and their correlation with patient breakpoints in lymphoid and nonlymphoid cancers. This dataset is related to Fig 2.
(XLSX)

S3 Data EMSA studies to determine binding of RAGs with heteroduplex DNA containing T/G mismatch and a cryptic nonamer derived from patient breakpoint region. Excel files showing the values for quantification of binding efficiency of cRAGs to DNA substrates *BCL1* MTC I, *BCL1* MTC II, *BCL1* MTCIII, *BCL1* MTCIV, *BCL2* ICRI, *BCL2* ICRII, *BCL2* MBR, *KMT2A*, *FGFR1* and *E2A*. This dataset is related to Fig 3E.
(XLSX)

S4 Data EMSA studies to determine binding of RAGs with non-specific substrate. Excel files showing the values for quantification of RAG binding efficiency to *BCL1* MTCII in presence of increasing concentration of unlabeled nonspecific DNA. This dataset is related to Fig 3G.
(XLSX)

S5 Data EMSA studies to determine binding of RAGs with specific substrate. Excel files showing the values for quantification of RAG binding efficiency to *BCL1* MTCII in presence of increasing concentration of unlabeled specific DNA. This dataset is related to Fig 3I.
(XLSX)

S6 Data Effect of manganese ion on RAG cleavage of DNA heteroduplex containing T/G mismatch and cryptic nonamer. Excel files showing the values for quantification of RAG cleavage efficiency at increasing concentration of MnCl_2_ (100, 200, 300, 400 and 500 μM) for DNA substrate EII. This dataset is related to Fig 4F.
(XLSX)

S7 Data Effect of manganese ion on RAG cleavage of DNA heteroduplex containing T/G mismatch and cryptic nonamer. Excel files showing the values for quantification of RAG cleavage efficiency at increasing concentration of MnCl_2_ (100, 200, 300, 400 and 500 μM) for DNA substrate MTCIV. This dataset is related to Fig 4H.
(XLSX)

S8 Data Evaluation of RAG nicking specificity on DNA substrates with T/G mismatch when an adjacent cryptic nonamer/s is present. Excel files showing the values for quantification of RAG nicking intensity on MTCIV derived DNA substrates with mutated cryptic nonamers. This dataset is related to Fig 5C.
(XLSX)

S9 Data To evaluate the impact of CAC on efficiency of RAG mediated cleavage. Excel files showing the values for quantification of RAG nicking intensity on MTCIV derived DNA substrates with mutated CAC and cryptic nonamers. This dataset is related to Fig 5E.
(XLSX)

S10 Data Chromatin immunoprecipitation of RAG1 for fragile genomic regions rich in CpGs and cryptic nonamers. Excel files showing the values for quantification of qPCR analysis of RAG1 ChIP of gene regions rich in CpGs and cryptic nonamers. This dataset is related to Fig 7C.
(XLSX)

S11 Data Evaluation of RAG mediated cleavage on DNA substrates mimicking intermediate stages of DNA repair with a nick or a gap. Excel files showing the values for quantification of RAG cleavage efficacy on DNA substrates bearing U/G mismatches with nonamer, ds DNA with nonamer alone, nick, or gap. This dataset is related to Fig 10C.
(XLSX)

S12 Data Western blot analysis to examine overexpression of AID in CEM cells. Excel files showing the values for quantification of AID expression after normalizing with ponceau stained membrane as loading control. This dataset is related to Fig 11C.
(XLSX)

S13 Data Evaluation of association between occurrence of CpGs and cryptic nonamers in whole genome. Excel files showing the values for incidence of breakpoint region in promoter and nonpromoter regions of genes, number of nonamers/kb in regions with breaks as compared to unbroken (control) regions of the human genome, percentage of windows with and without CpGs in genic region along with CpGs that are broken and unbroken, distribution of breakpoints in euchromatin and heterochromatin regions and their correlation with occurrence of CpGs and nonamers, number of CpGs and cryptic nonamers per 100 bp region between breakpoints of lymphoid and nonlymphoid translocations from the same genes. This dataset is related to S2A–S2D, S2G Fig.
(XLSX)

S14 Data Evaluation of impact of mismatches and cryptic nonamers on RAG cleavage when present on different random regions of the genome where no breakpoints are reported. Excel files showing the values for quantification for RAG cleavage on 3 random regions in presence of either a CpG or a mismatch. This dataset is related to S6C Fig.
(XLSX)

S15 Data Evaluation of impact of mismatches and cryptic nonamers on RAG cleavage when present on different random regions of the genome where no breakpoints are reported. Excel files showing the values for quantification of RAG cleavage on AT and GC rich regions devoid of breakpoints. This dataset is related to S6F Fig.
(XLSX)

S16 Data Chromatograms showing breakpoint junctions obtained following PCR, cloning and sequencing of LM-PCR products derived from KMT2A gene when analyzed from CEM cells. (ZIP)

S17 Data Chromatograms showing breakpoint junctions obtained following PCR, cloning and sequencing of LM-PCR products when analysed from Nalm6 cells. (ZIP)
