# Supplementary figures and images for: Nonamer dependent RAG cleavage at CpGs can explain mechanism of chromosomal translocations associated to lymphoid cancers
Source: PLoS Genet. 2022 Oct 13;18(10):e1010421. doi: 10.1371/journal.pgen.1010421 (PMC9595545; doi:10.1371/journal.pgen.1010421)

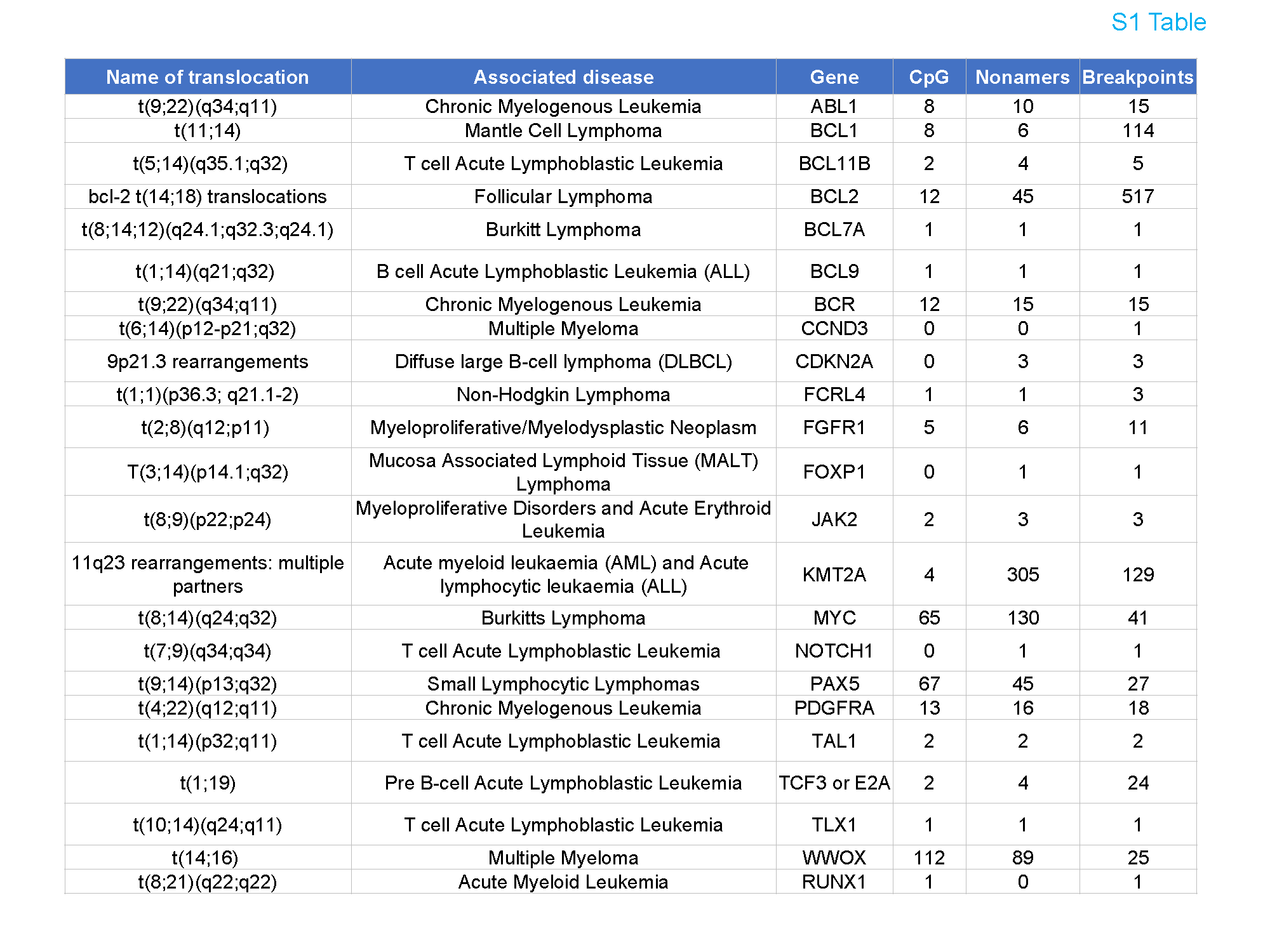

Supplement: S1 Table — The table shows detailed information on translocations, genes involved, associated different lymphoid malignancies along with number of CpG sites and cryptic nonamers in the vicinity of breakpoints found in the analysed genes. (TIFF) [file pgen.1010421.s001.tiff]

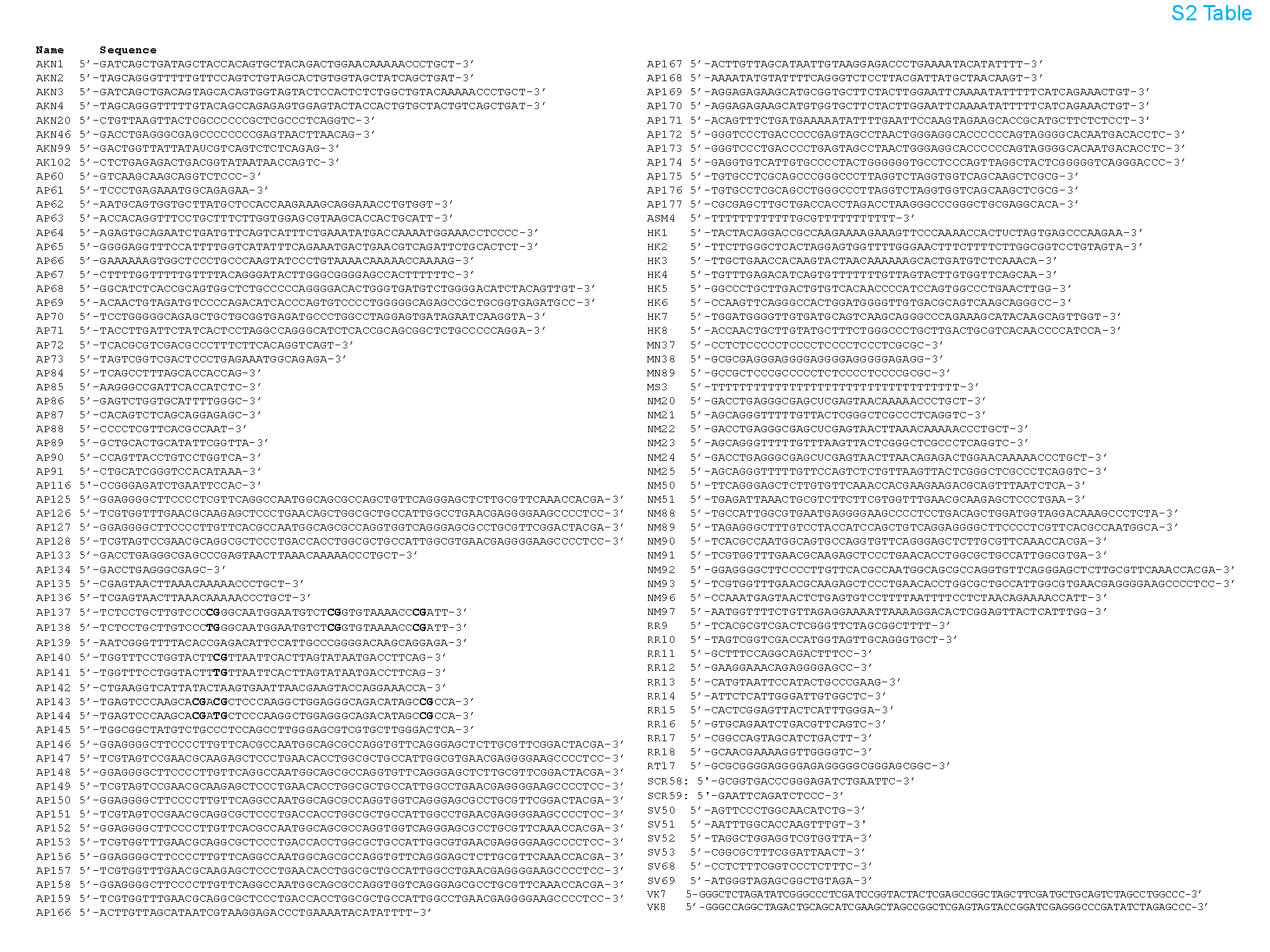

Supplement: S2 Table — (TIFF) [file pgen.1010421.s002.tiff]

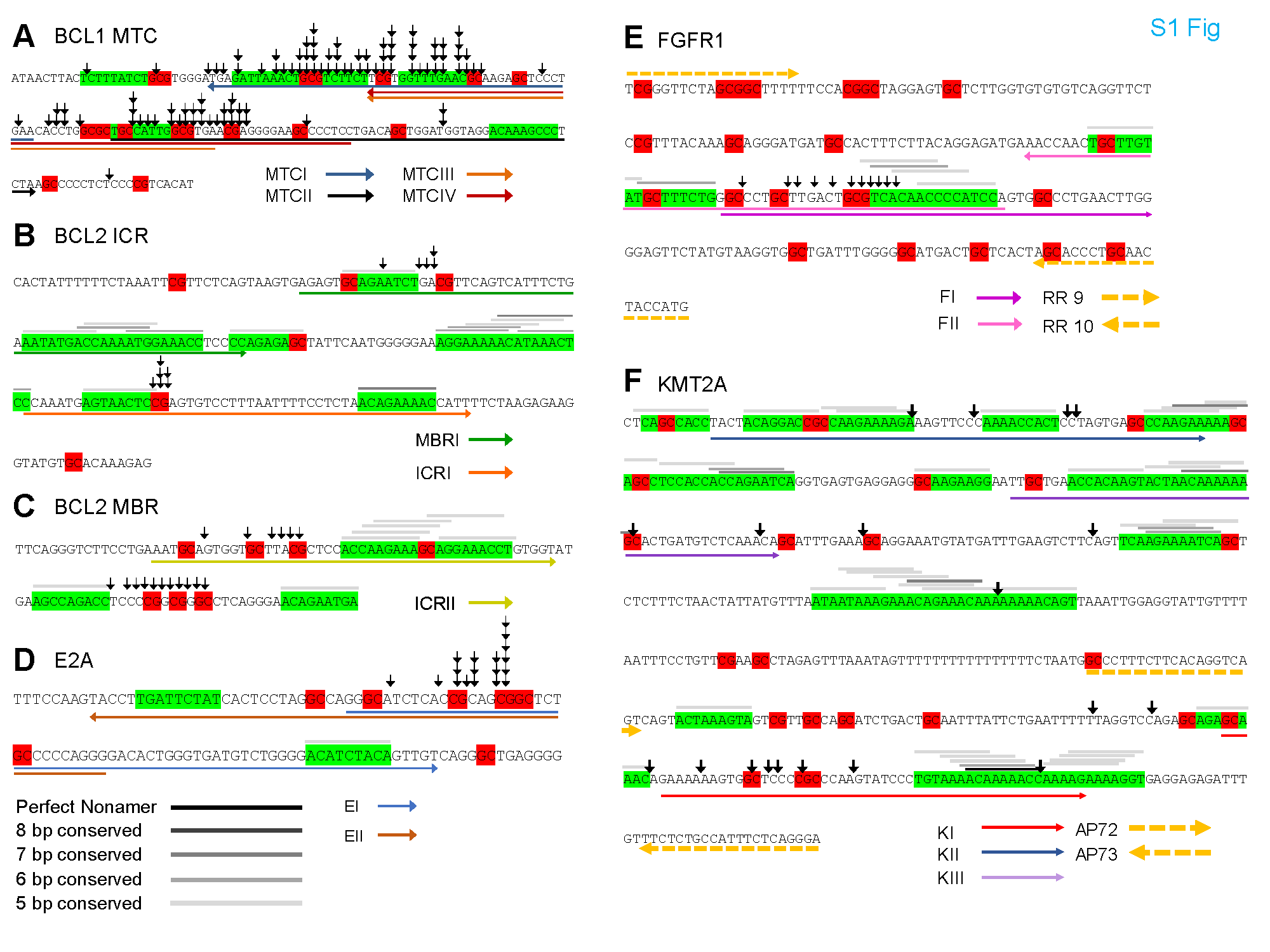

Supplement: S1 Fig — A-F. Sequence of breakpoint regions mapped from lymphoid malignancies for the occurrence of chromosomal translocation breakpoints near cryptic nonamers and CpGs of selected genes BCL1 MTC (A), BCL2 ICR (B), BCL2 MBR (C), E2A (D), FGFR1 (E), KMT2A (F). Each breakpoint is represented by an arrowhead adjoining the breakpoint site. Red highlighted sequence indicates CpG sites. Green highlighted sequence indicates cryptic nonamers. Different intensity grey lines on the top of gene sequence corresponds to cryptic nonamers with different levels of conservations with respect to the canonical nonamer. Different colored lines at bottom of gene sequence indicated sequence of DNA substrates used in gel-based assay. (TIFF) [file pgen.1010421.s003.tiff]

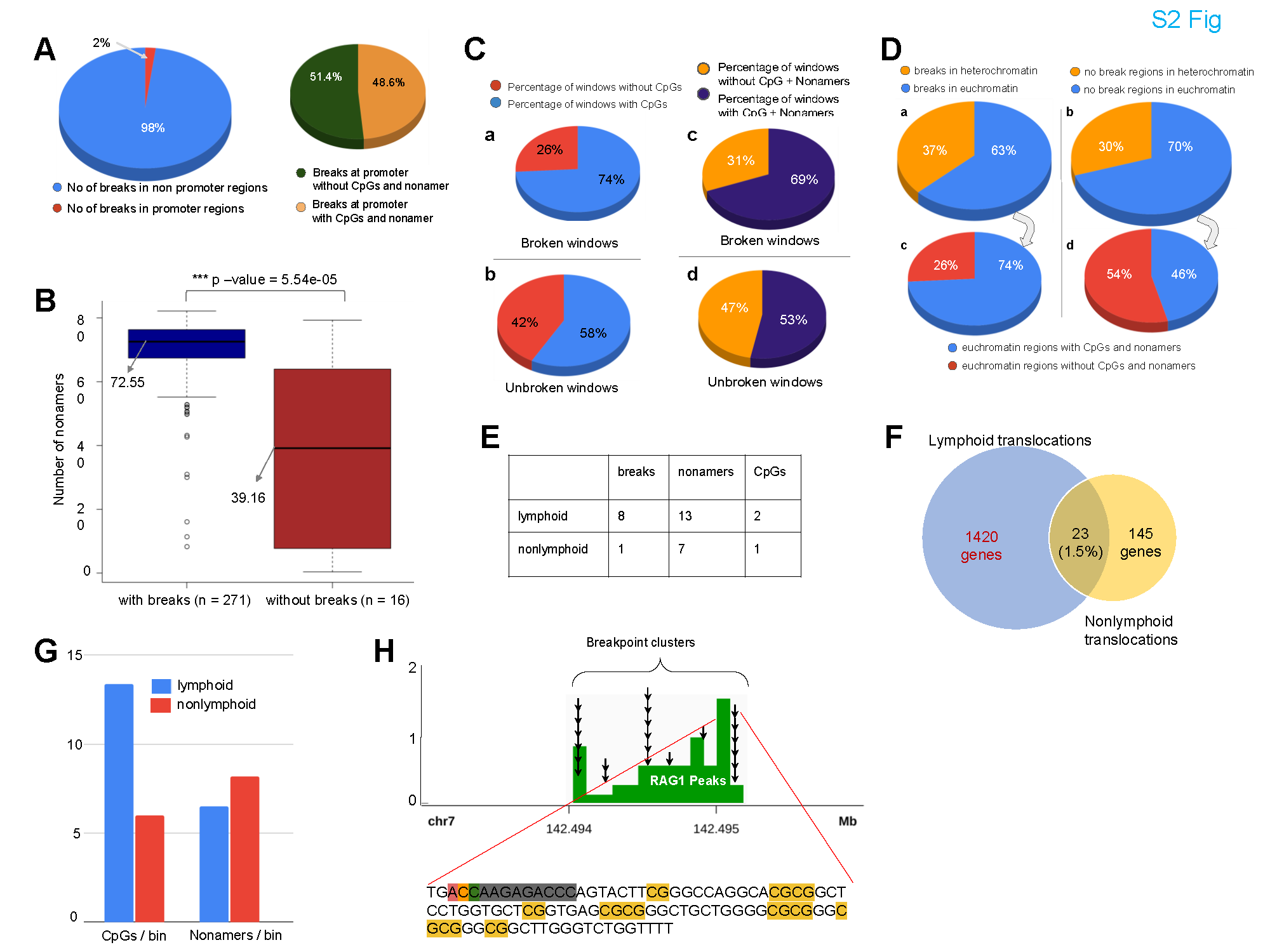

Supplement: S2 Fig — A. Pie chart showing comparison of incidence of breakpoint region in promoter and nonpromoter regions of genes. The right panel shows comparison of occurrence of CpGs and nonamer when breakpoints are seen in promoters. B. A boxplot showing number of nonamers/kb in regions with breaks as compared to unbroken (control) regions of the human genome. C. The pie charts depict percentage of windows with and without CpGs in genic region that are broken (a) and unbroken (b). The percentage of windows with and without CpGs along with cryptic nonamers in broken (c) and unbroken (d) genic regions are also shown. In the case of unbroken region equivalent number of random windows containing genic regions were selected. D. comparison of distribution of breakpoints in euchromatin and heterochromatin regions and their correlation with occurrence of CpGs and nonamers. a) Pie chart showing distribution of breakpoint regions in euchromatin and heterochromatin regions. b) Pie chart showing distribution of equivalent length “no-break” random regions in euchromatin and heterochromatin regions. c) Pie chart showing distribution of euchromatic break regions containing CpG and cryptic nonamers. d) Pie chart showing distribution of euchromatic “no-break” regions containing CpG and cryptic nonamers. E. Table showing distribution of breakpoints, cryptic nonamers and CpGs in case of lymphoid and nonlymphoid tumor samples. F. A Venn diagram showing common genes (1.5%) between lymphoid and nonlymphoid translocations. G. Bar graph showing the difference in number of CpGs, cryptic nonamers per 100 bp region, between breakpoints of lymphoid and nonlymphoid translocations from the same genes. H. A ChIP seq plot showing the peak intensity of RAG1 binding over a locus of TCRVB gene, along with breakpoints (represented with black arrows) from our studies clustered around the same location. One of the peaks has been highlighted and the sequence has been expanded to show the presence of cryptic nonamers in [file pgen.1010421.s004.tiff]

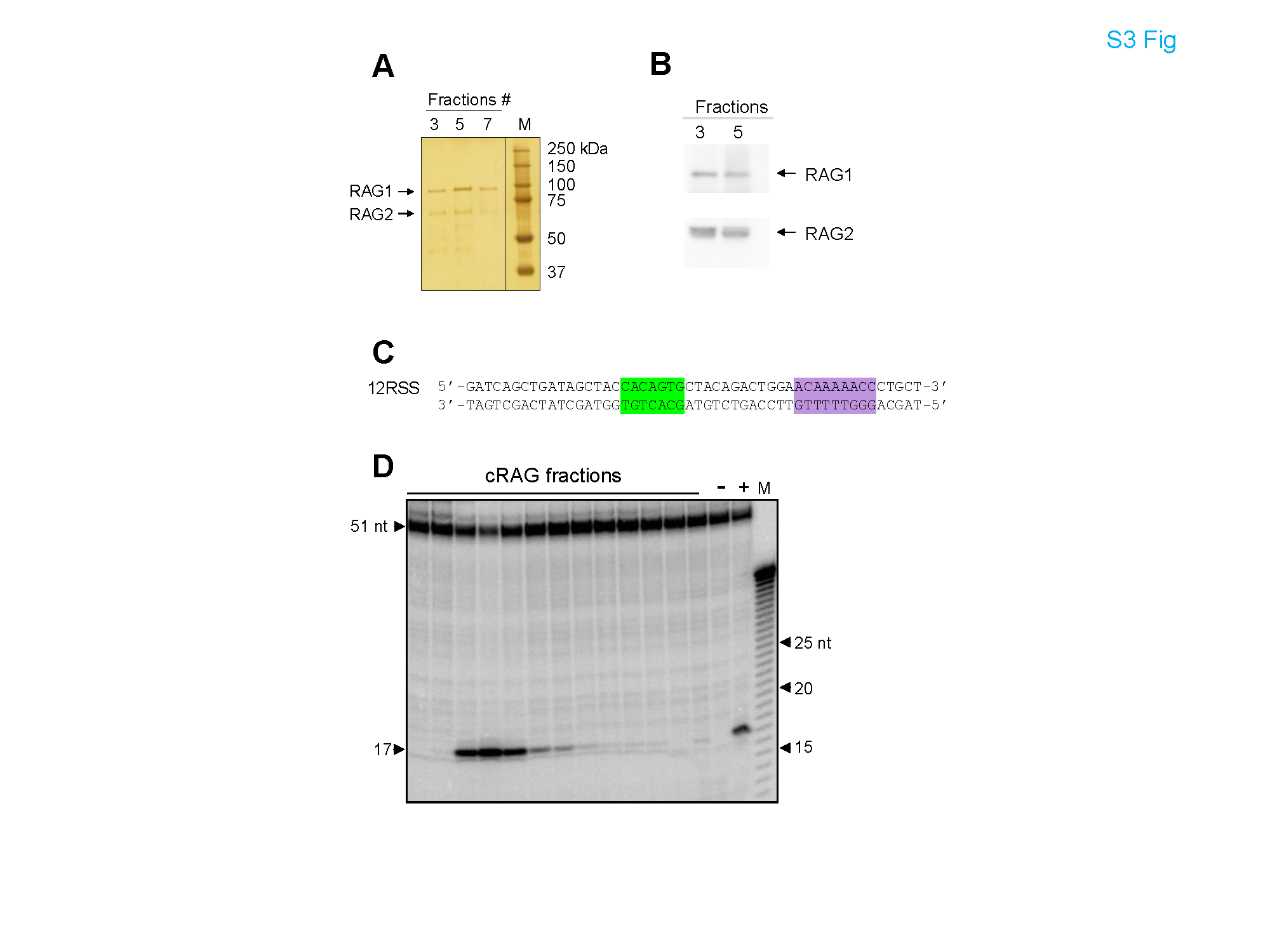

Supplement: S3 Fig — A. Gel profile showing purified cRAGs. ‘M’ is molecular weight ladder. B. Western blot showing confirmation of identity of cRAGs from 293T cells. Identity of the proteins was checked using anti RAG1 and RAG2. C. Sequence of 12RSS substrate used for activity assay. D. Activity assay of purified cRAGs on 12RSS substrate. DNA was subjected to RAG mediated cleavage and the products were resolved on a 15% denaturing polyacrylamide gel. The cleavage products are indicated with an arrowhead. The fractions that exhibited DNA cleavage activity were used for the further assays. (TIFF) [file pgen.1010421.s005.tiff]

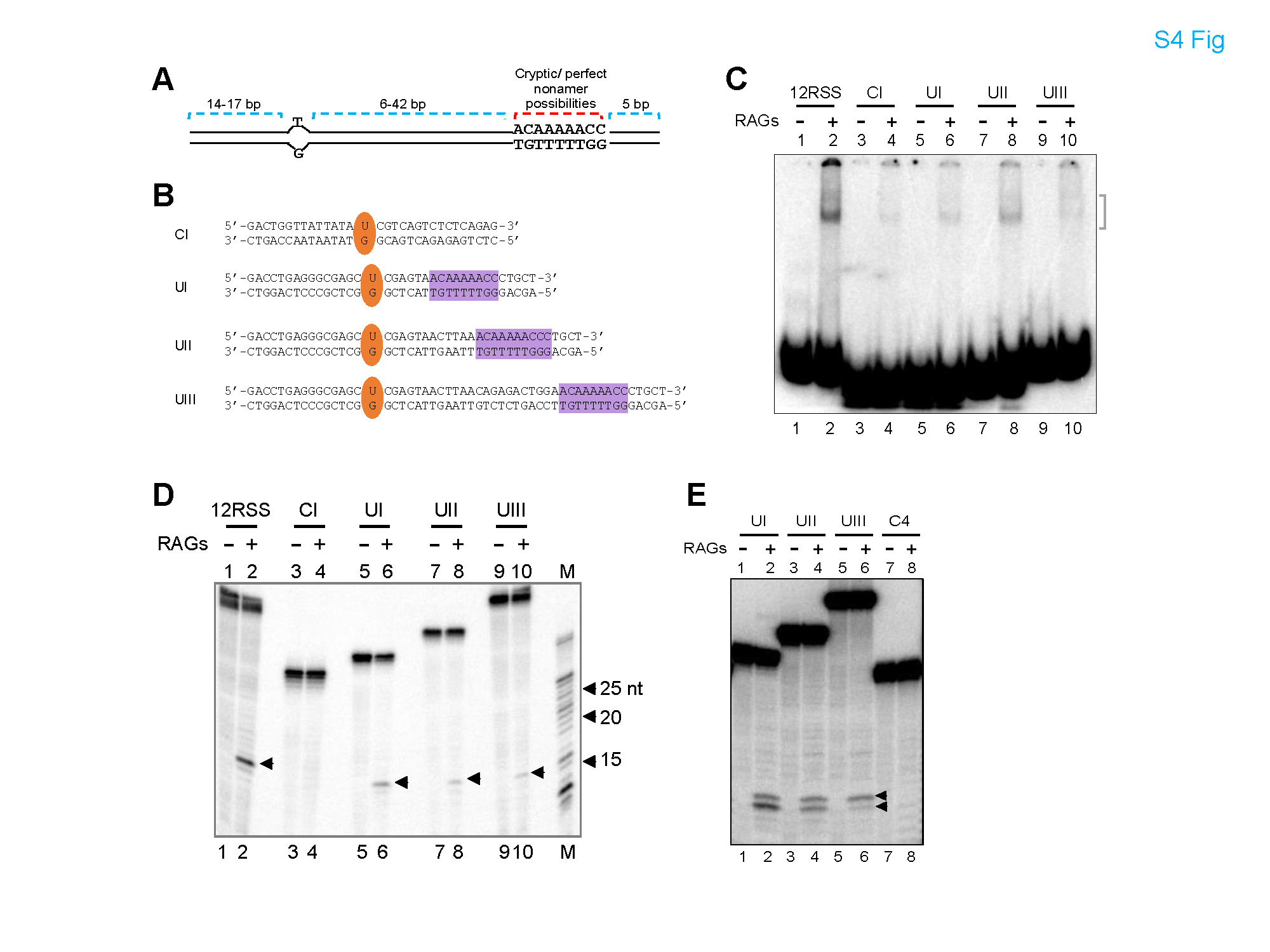

Supplement: S4 Fig — A. General design of DNA substrates used in the assays. B. Schematic of heteroduplex DNA substrates harboring U/G mismatch and V(D)J nonamer (canonical). Oligomers are designed to harbor U/G mismatch alone (CI), or U/G mismatch and canonical nonamer placed 6 nt (UI), 12 nt (UII), 23 nt (UIII) downstream to mismatch. C. Gel profile showing RAG binding of DNA substrates containing U/G mismatch and canonical nonamer. D, E. Gel profile showing RAG cleavage assay on DNA substrates containing U/G mismatch and canonical nonamer. For cleavage assay, 15% denaturing gel was used. The cleaved product is marked with an arrowhead. M is molecular weight marker. C4 and CI are controls that correspond to random DNA control and U/G mismatch without nonamer, respectively. (TIFF) [file pgen.1010421.s006.tiff]

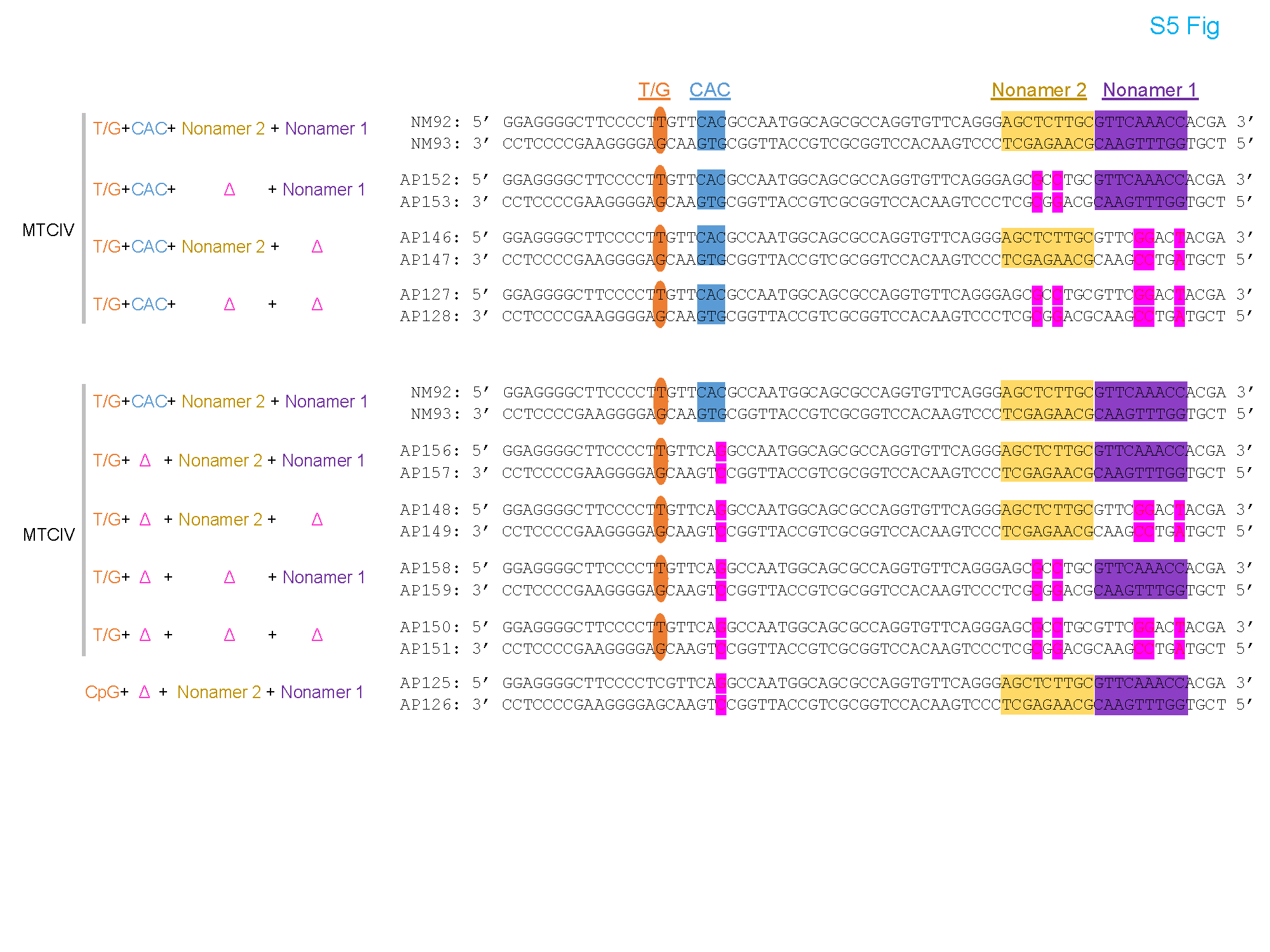

Supplement: S5 Fig — Corresponding oligomers were annealed to form a double stranded DNA with either T/G mismatch or mutated cryptic nonamer 1, 2 or both. CAC downstream to T/G mismatch is shown in blue. T/G mismatch is shown in orange oval, whereas cryptic nonamer is shown in purple and yellow. Mutated nucleotides to abrogate existing nonamer or CAC are highlighted in pink (TIFF) [file pgen.1010421.s007.tiff]

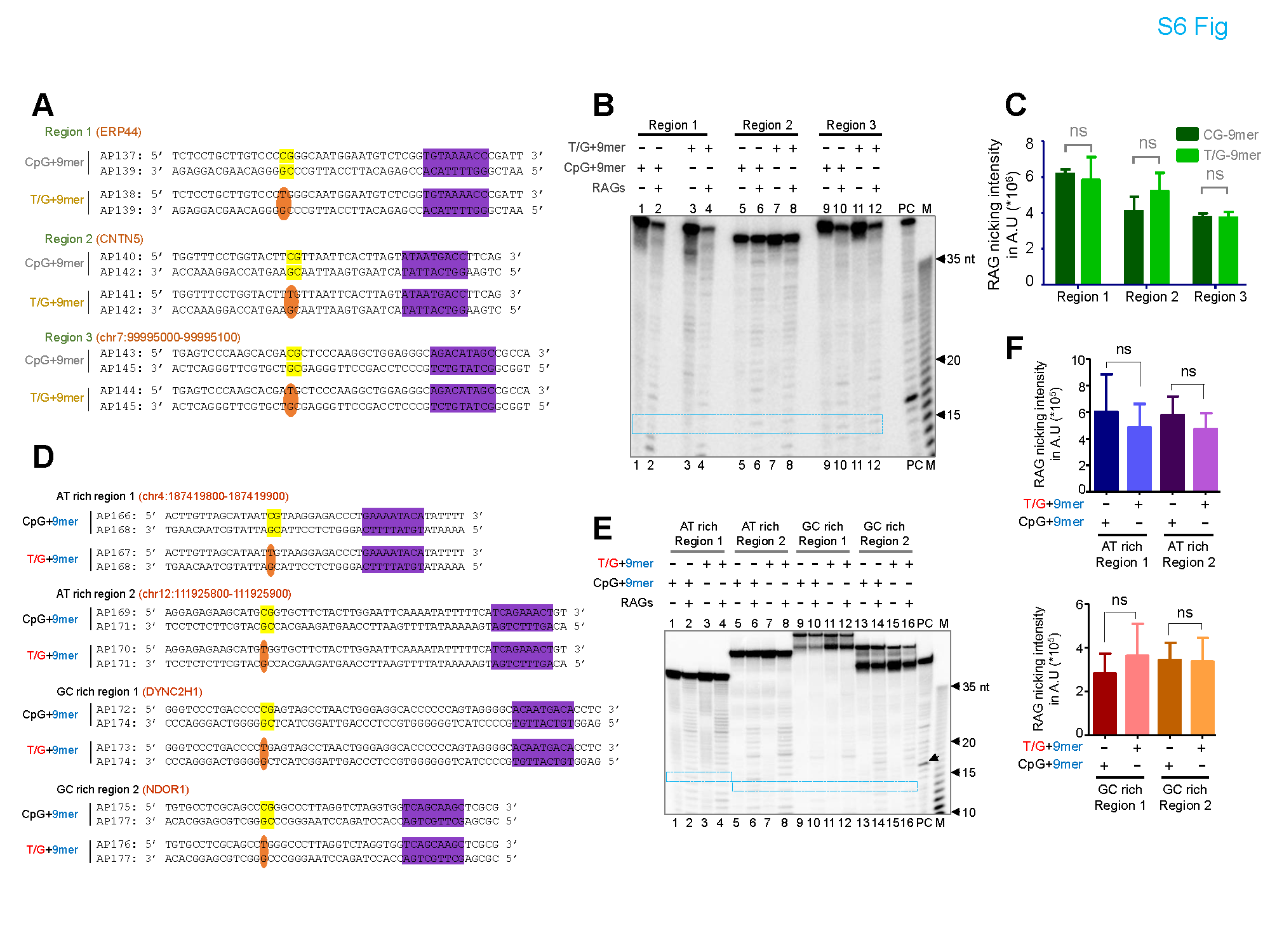

Supplement: S6 Fig — A. Schematic showing sequences of random DNA substrates (3 regions) used for the study with mismatches and cryptic nonamers, where no breakpoints are reported. Orange oval indicates T/G mismatch, purple highlight indicates nonamer whereas CpG nearby nonamer is indicated by yellow. Corresponding oligomers were annealed to form a double stranded DNA with either T/G mismatch and cryptic nonamer. B. Gel profile showing cleavage by RAGs on DNA substrates derived from regions devoid of breakpoints depicted in panel A. C. Quantification showing RAG cleavage on 3 random regions in presence of either a CpG or a mismatch. D. Schematic showing sequences of random DNA substrates (2 AT and 2 GC rich) used for the study with mismatches and cryptic nonamers, where no breakpoints are reported. Orange oval indicates T/G mismatch, purple highlight indicates main cryptic nonamer whereas CpG nearby nonamer is indicated by yellow. E. Gel profile showing cleavage by RAGs on DNA substrates derived from 2 AT and 2 GC rich regions devoid of breakpoints. ‘PC’ is positive control in which RAG cleavage reaction was performed using labelled 12RSS. ‘M’ is 1 nt Klenow ladder. F. Quantification showing RAG cleavage on AT and GC rich regions devoid of breakpoints. Error bar was calculated as mean ± SEM. *p < 0.05, **p < 0.005, ***p <0.0001. ns, not significant; AU is arbitrary unit. (TIFF) [file pgen.1010421.s008.tiff]

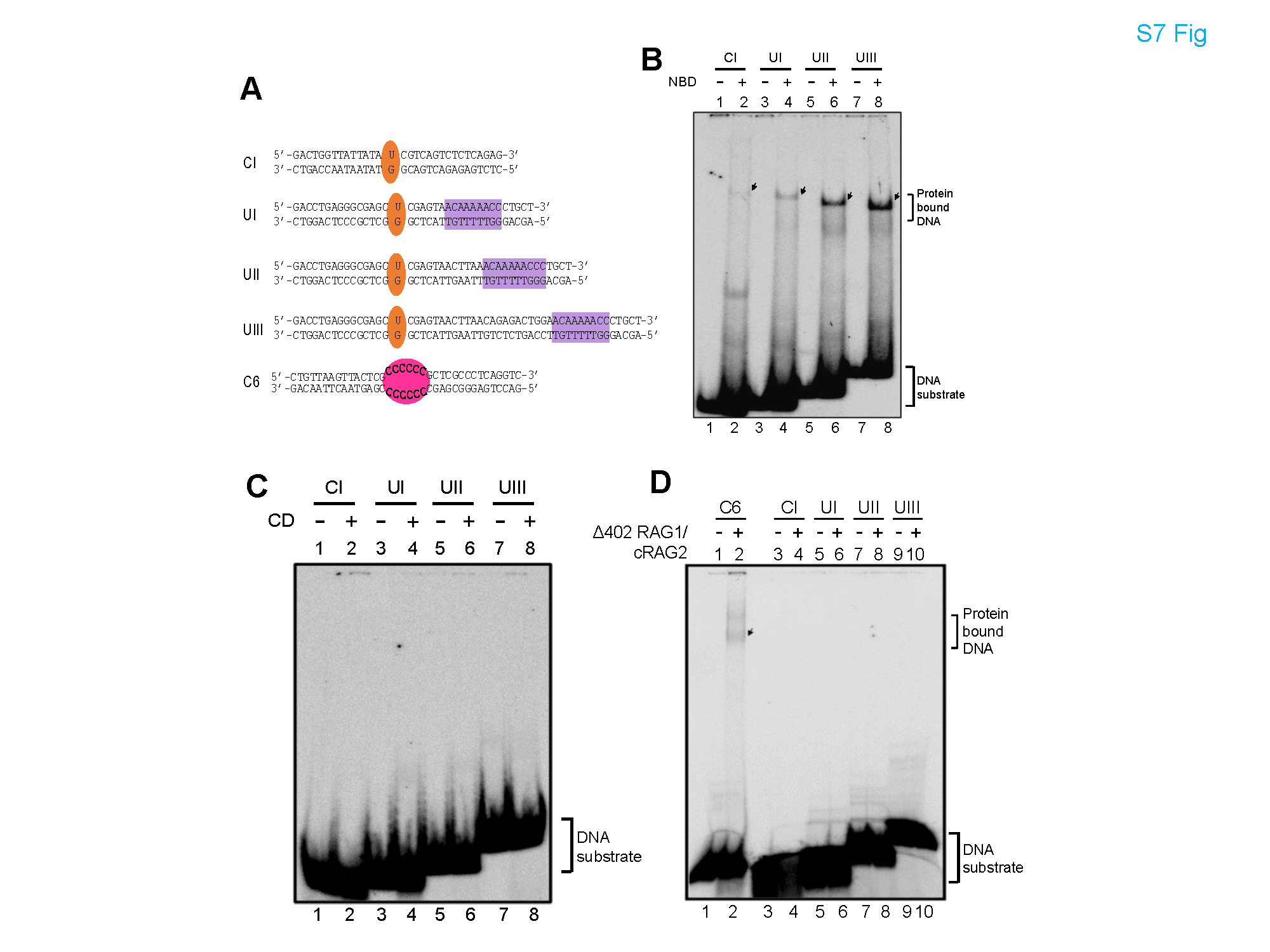

Supplement: S7 Fig — A. Diagrammatic representation of oligomeric DNA substrates used in the study. The oligomeric DNA substrates containing U/G mismatch alone (C1) and those with a canonical nonamer placed 6 (UI), 12 (UII) and 23 (UIII) bp downstream to it are shown. Besides, C6 bubble is also shown. B. Native gel profile showing the binding of nonamer binding domain (NBD) of RAG1. C. Native gel profile showing the binding of central domain of RAG1. D. Binding profile of NBD deleted cRAG1/ cRAG2 complex on DNA harboring U/G mismatch with a canonical nonamer placed 6, 12 and 23 nt away from it or a heteroduplex DNA with cytosine bubble. The bands due to RAG binding are indicated by arrows in panels B-D. (TIFF) [file pgen.1010421.s009.tiff]

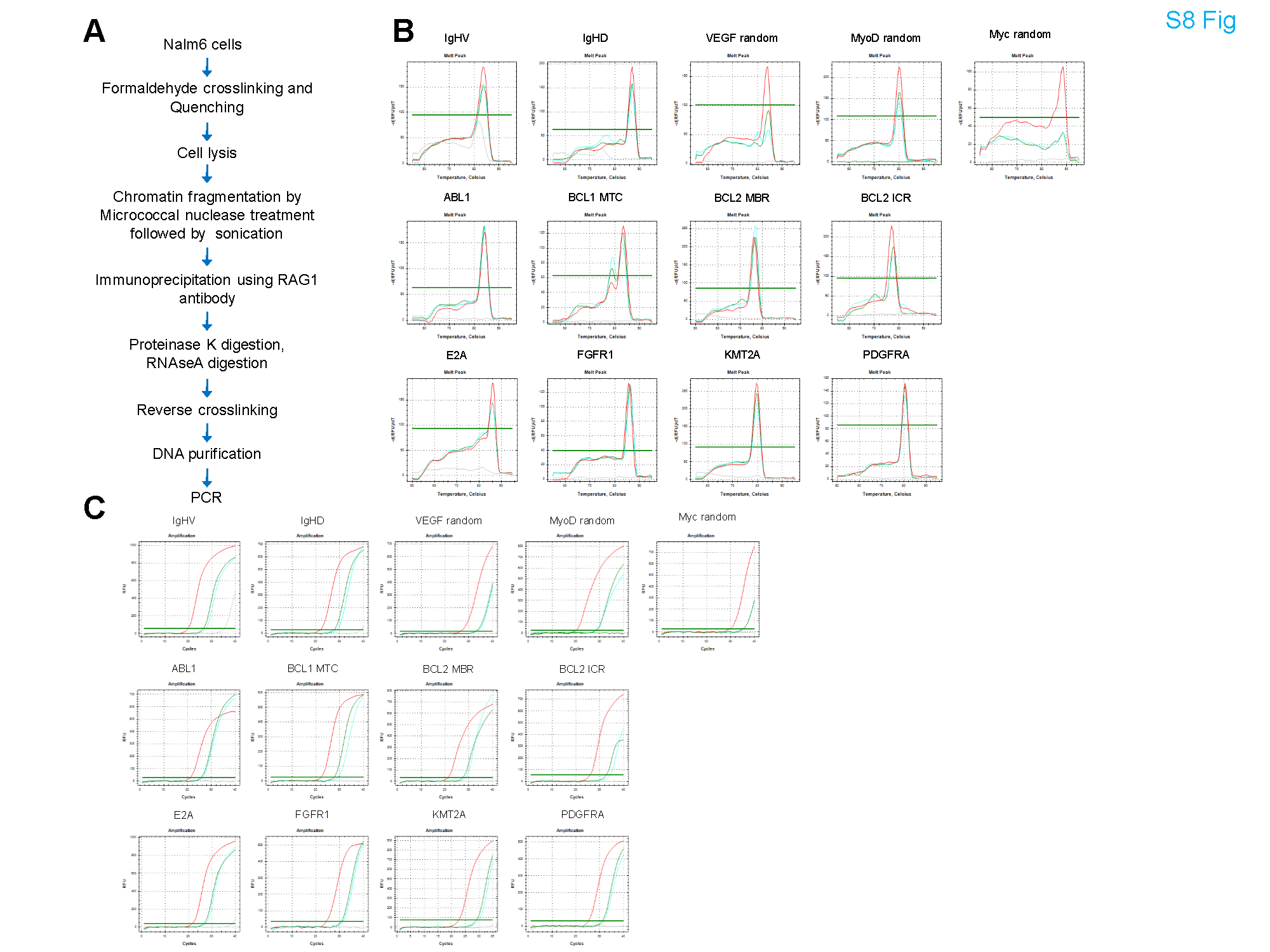

Supplement: S8 Fig — A. Procedure used for RAG1 chromatin immunoprecipitation. B. Melt curves for each of the 13 genes used in ChIP qPCR analysis. C. Representative amplification curves (green represents the control amplification curve and red depicts the experimental regions) for all genes analyzed are presented. (TIFF) [file pgen.1010421.s010.tiff]

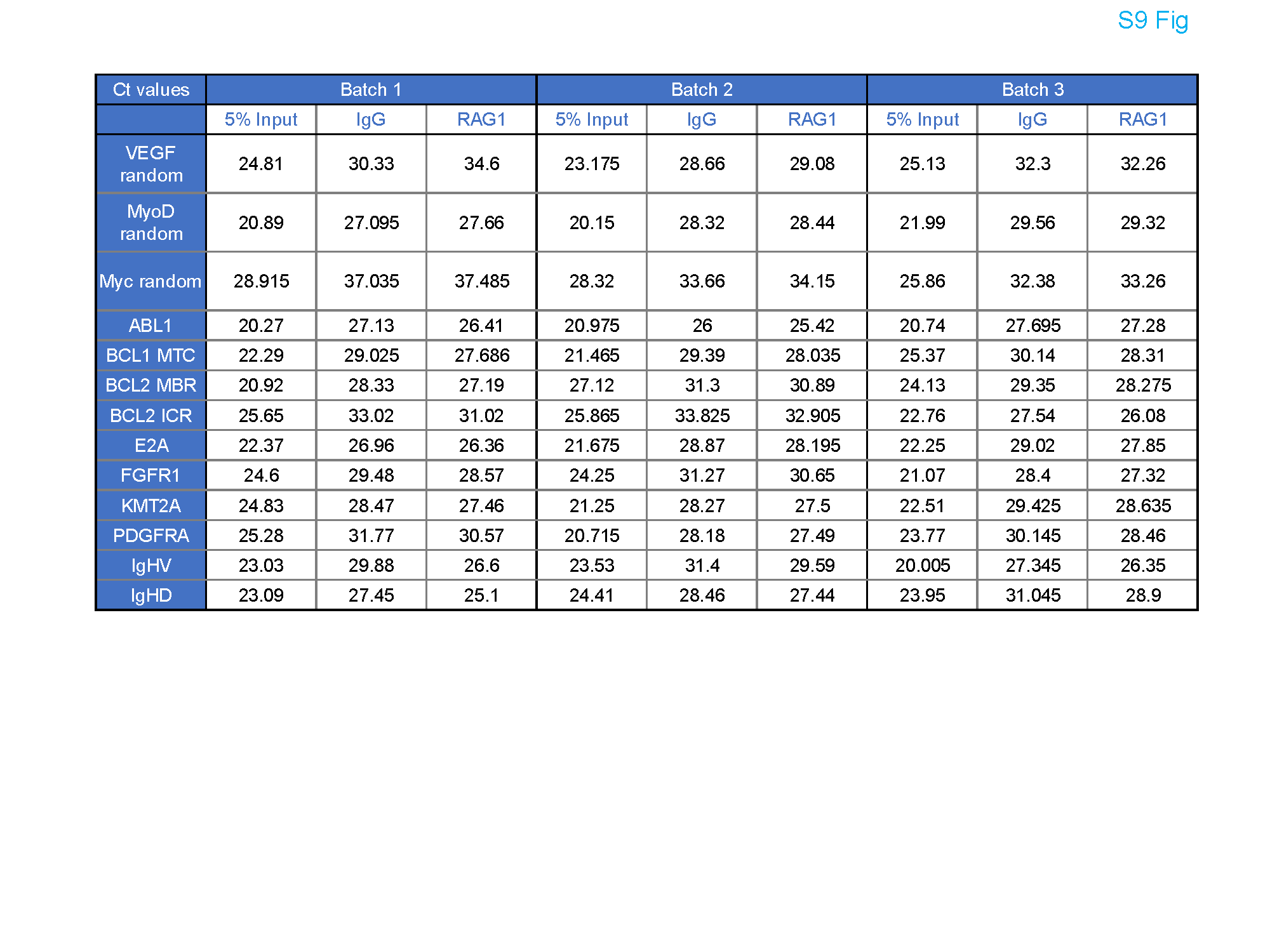

Supplement: S9 Fig — Ct values obtained for all 13 genes analyzed using ChIP qPCR are shown. (TIFF) [file pgen.1010421.s011.tiff]

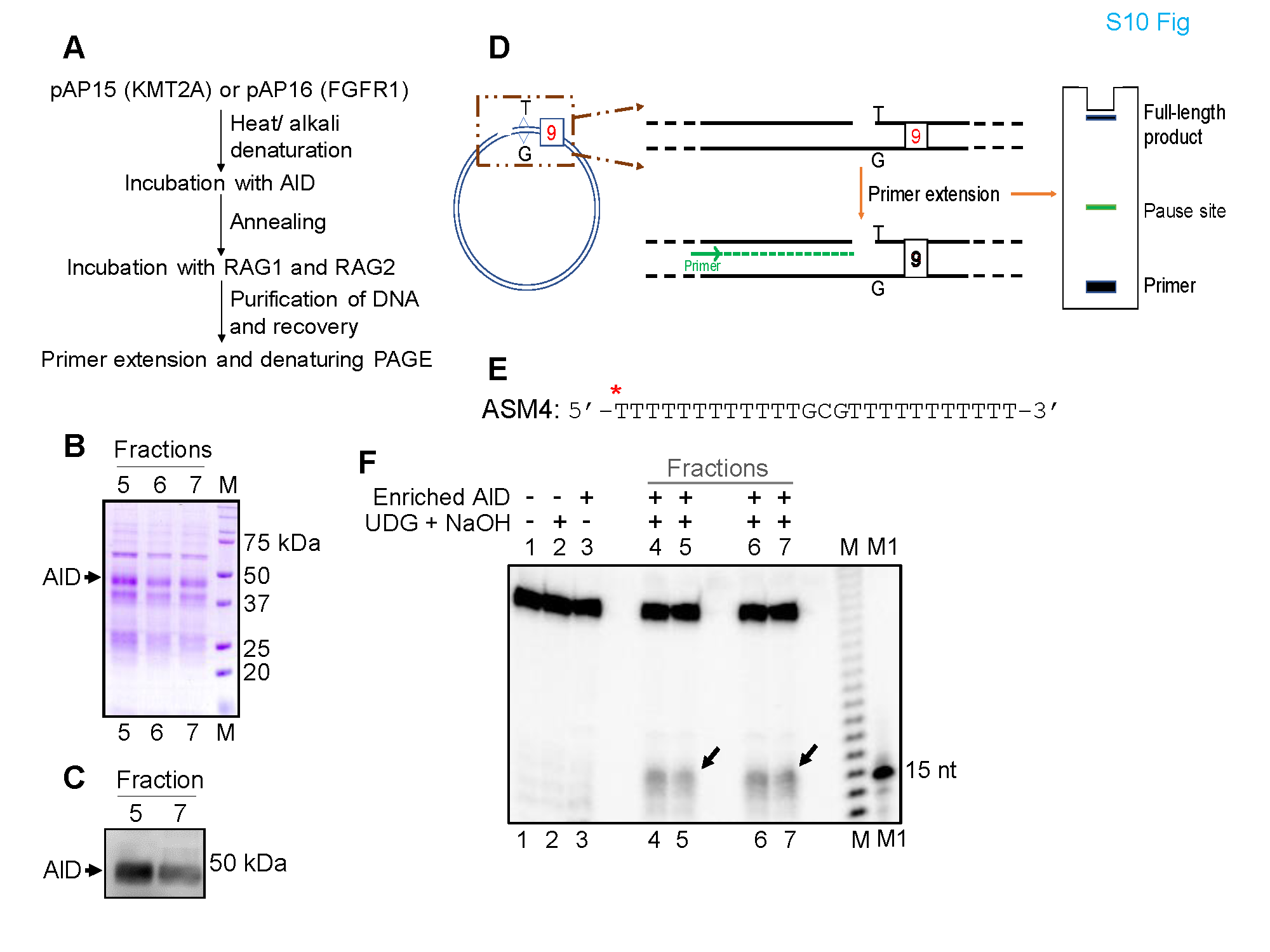

Supplement: S10 Fig — A. Schematic showing summary of steps involved in in vitro reconstitution assay. B. SDS-PAGE profile showing purified GST-AID. C. Western blot for purified GST tagged AID. D. Pictorial representation of primer extension assay used for detecting RAG-induced breaks. After the treatment of plasmid with AID and RAGs, DNA strand is amplified in primer extension assay separately using single primer so that RAGs induced single-stranded breaks can be detected as pause sites when resolved on 8% denaturing PAGE. E. Oligomeric DNA used in activity assay of purified AID. F. Oligomeric substrate-based activity assay for purified AID. Expected cleavage product of 13 bp is indicated with arrow. (TIFF) [file pgen.1010421.s012.tiff]

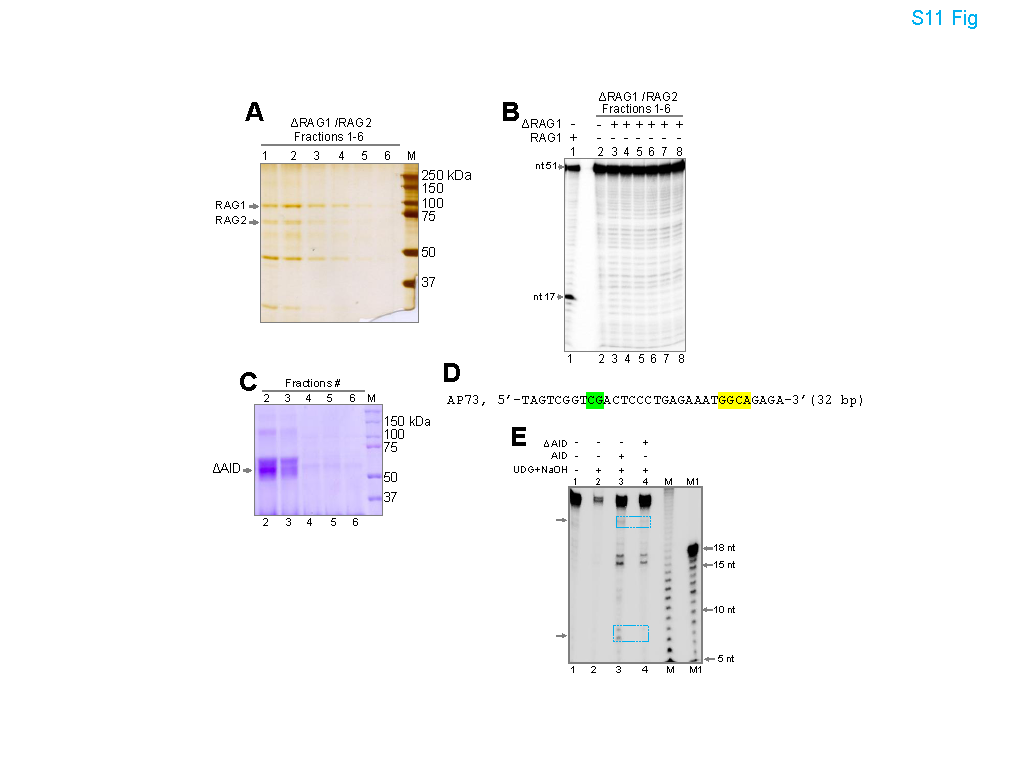

Supplement: S11 Fig — A. Silver-stained gel profile of ΔcRAGs (pEBGRAG1 D708A and GST tagged cRAG2). ΔcRAGs were overexpressed in HEK293T cell line and purified. ΔcRAG1 and cRAG2 bands are indicated using arrows. B. Activity assay of purified ΔcRAGs on 12RSS substrate. Lane 1 is positive control in which 12RSS was incubated with cRAGs (wild type). Lane 3–8 are reactions products obtained following incubation of 12RSS with purified fractions of ΔcRAGs. Radiolabelled substrate DNA (12RSS) was loaded in lane 2. C. CBB profile of ΔAID in which deaminase domain is mutated (H56R, E58Q). D. Oligomeric DNA used for activity assay to test the purified ΔAID and its wild type. DGYW and CpG motifs are highlighted in yellow and green color, respectively. E. Comparison of activity of purified ΔAID with that of wild type. Bands of interest are indicated using arrows and blue box. (TIFF) [file pgen.1010421.s013.tiff]

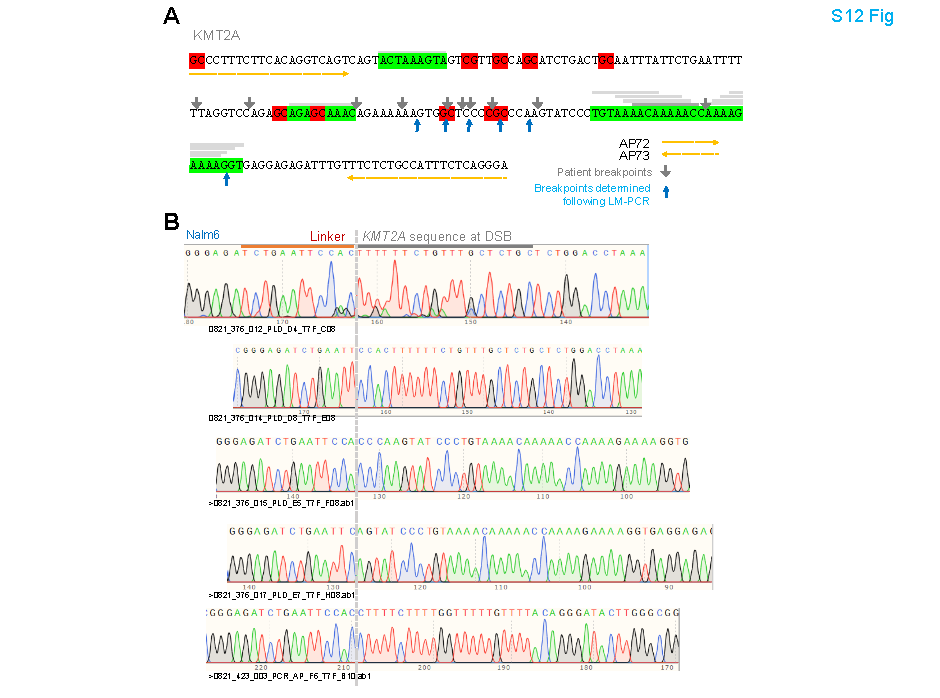

Supplement: S12 Fig — Schematic of KMT2A breakpoint seen in patents mapped along with LM-PCR breakpoints detected in our assays. Grey downward arrows represent patient breakpoints, while blue upward arrows indicate breakpoints determined following LM-PCR in this study. B. Chromatogram of breakpoint junctions obtained following PCR and cloning. Left side represents linker sequence whereas sequence in the right side of the vertical line indicates KMT2A sequence. (TIFF) [file pgen.1010421.s014.tiff]

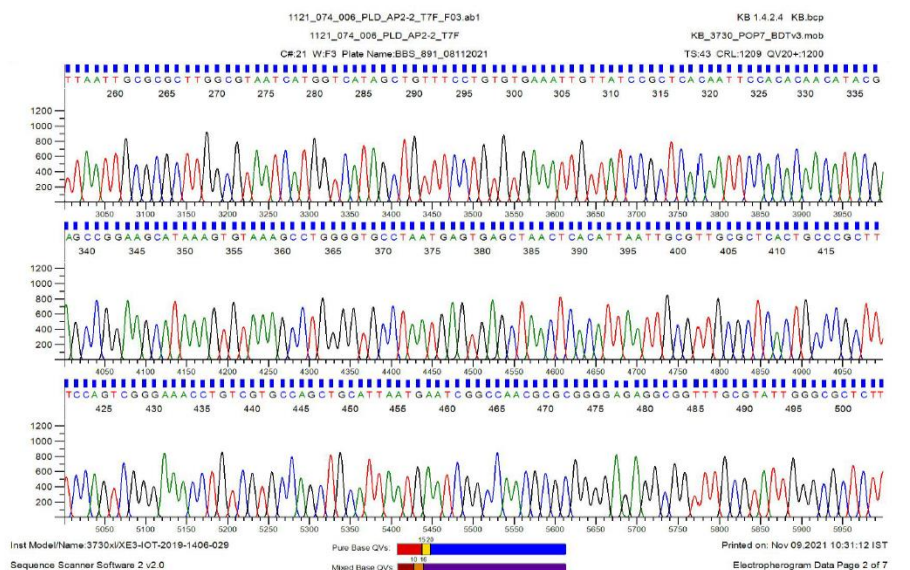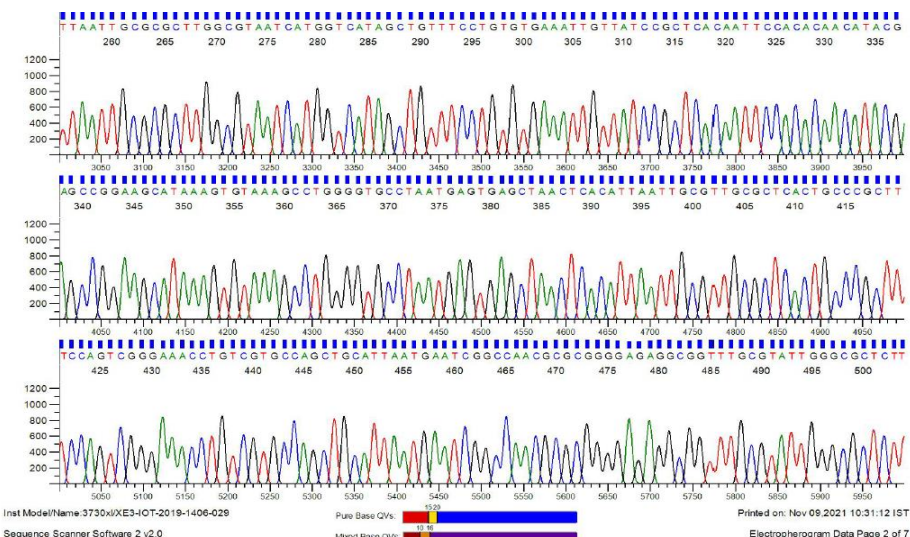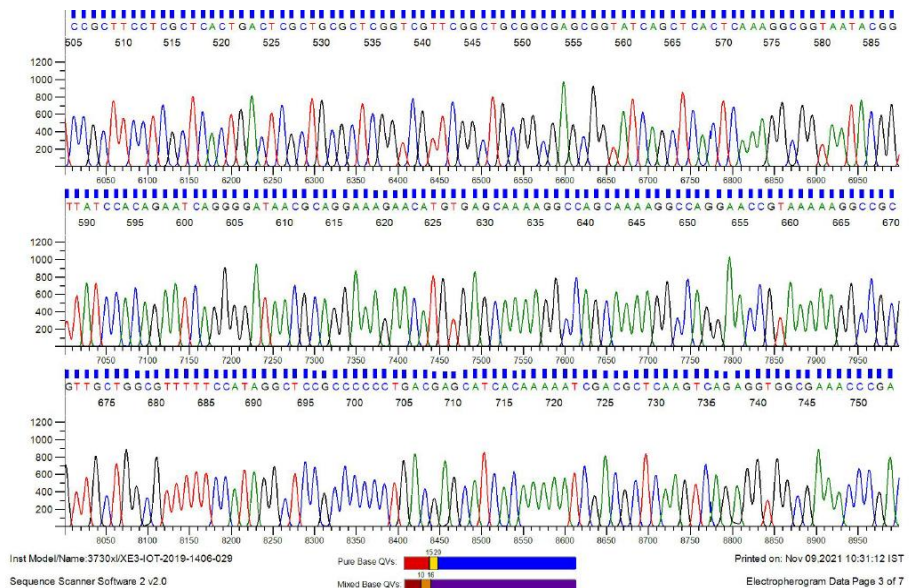

Supplement: S16 Data — (ZIP) [file pgen.1010421.s030.zip › Fig11_E_chromatograms_pdf/1121_074_006_PLD_AP2-2_T7F_F03.pdf]

KB 1.4.2.4 KB.bcp  
KB\_3730\_POP7\_BDTv3.mob  
TS:43 CRL:1122 QV20+:1171

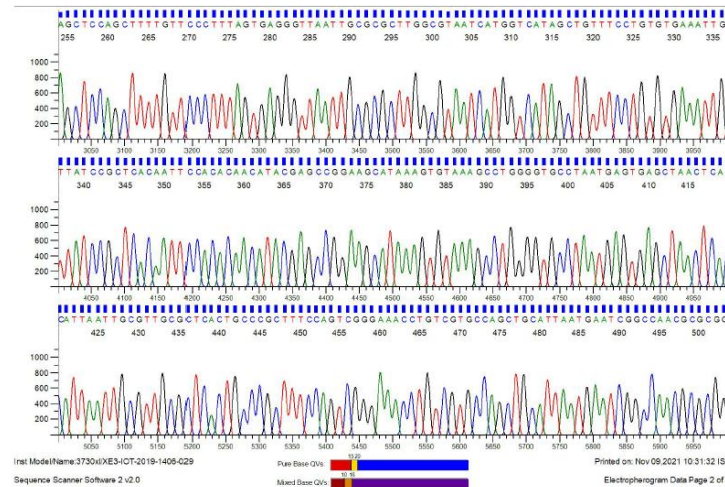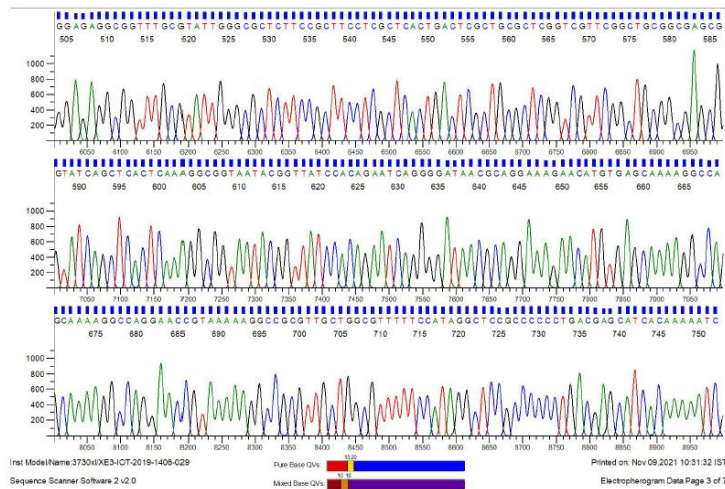

Supplement: S16 Data — (ZIP) [file pgen.1010421.s030.zip › Fig11_E_chromatograms_pdf/1121_074_010_PLD_AP3-2_T7F_B04.pdf]

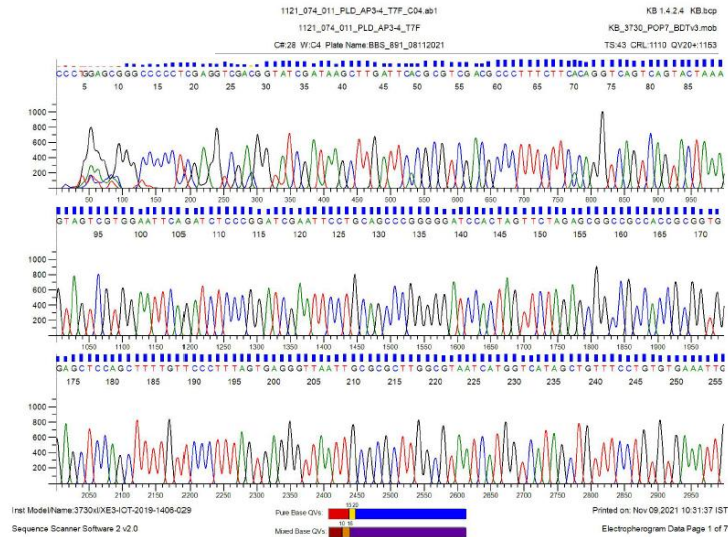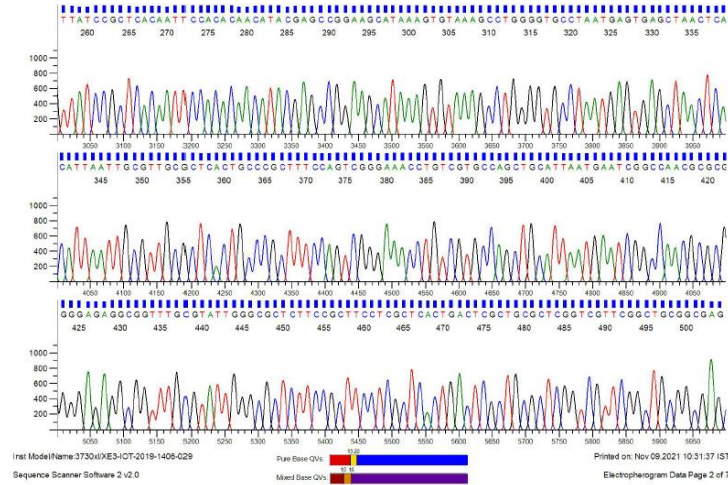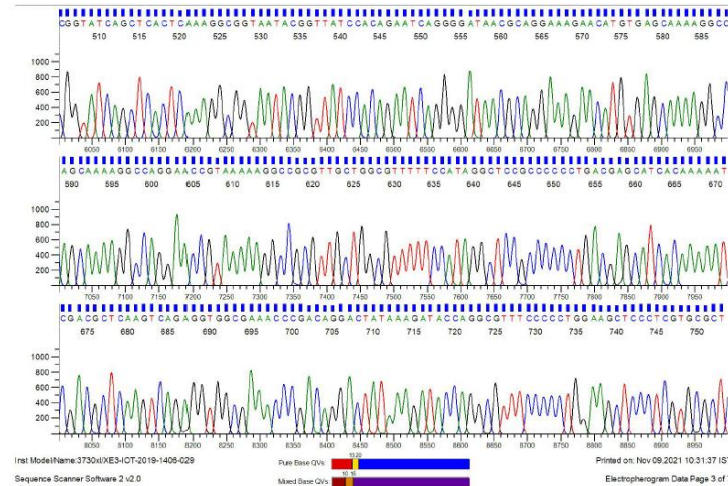

Supplement: S16 Data — (ZIP) [file pgen.1010421.s030.zip › Fig11_E_chromatograms_pdf/1121_074_011_PLD_AP3-4_T7F_C04.pdf]

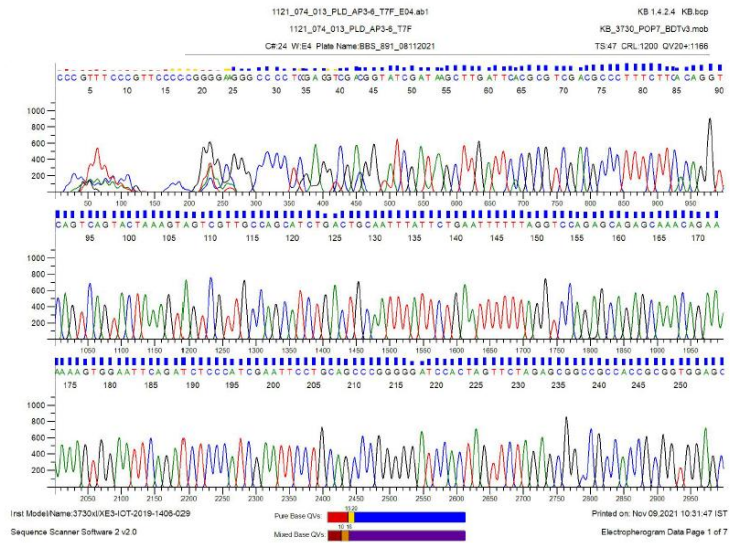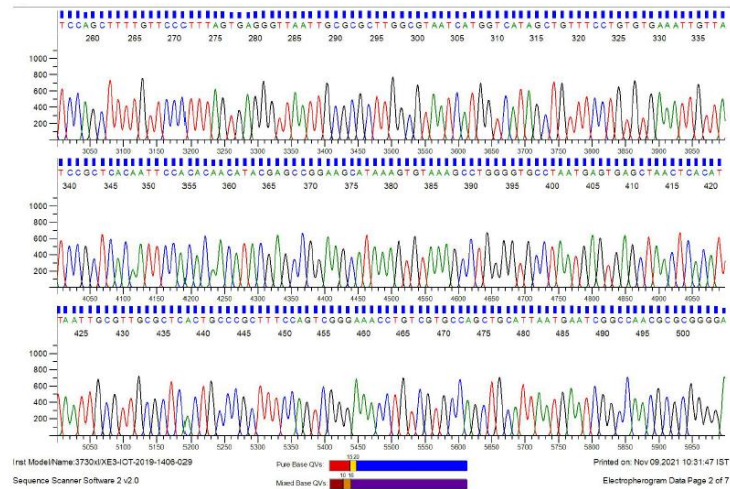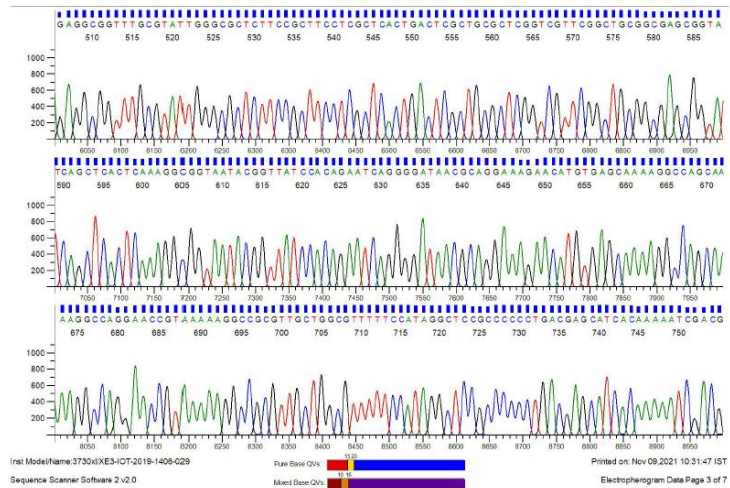

Supplement: S16 Data — (ZIP) [file pgen.1010421.s030.zip › Fig11_E_chromatograms_pdf/1121_074_013_PLD_AP3-6_T7F_E04.pdf]

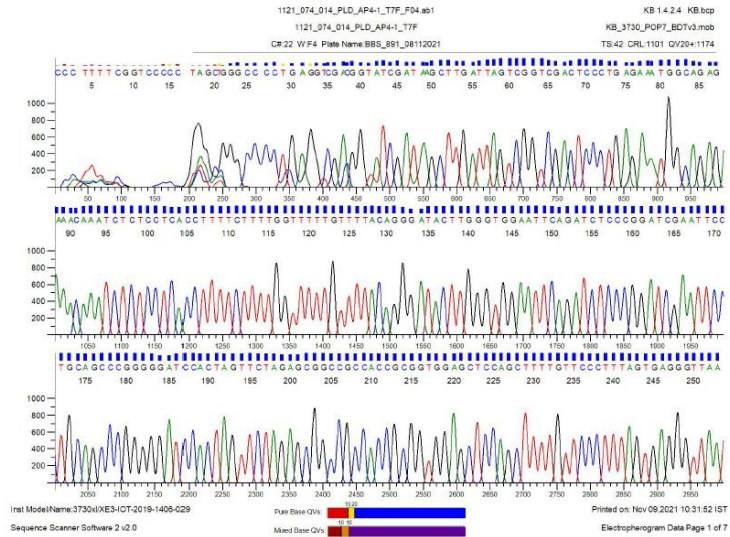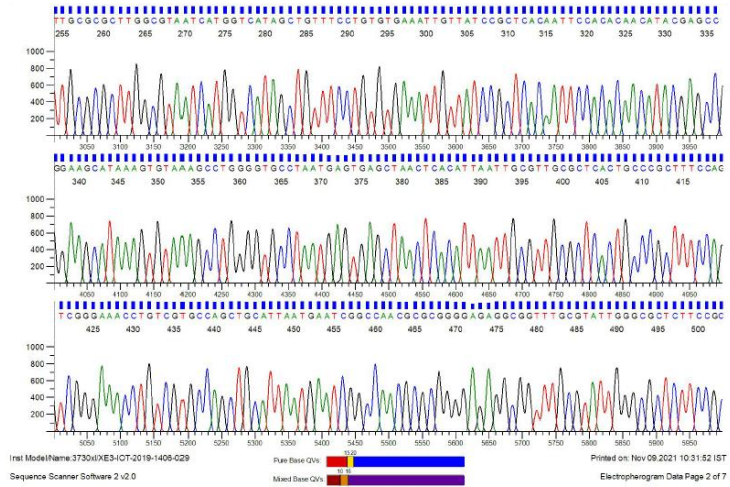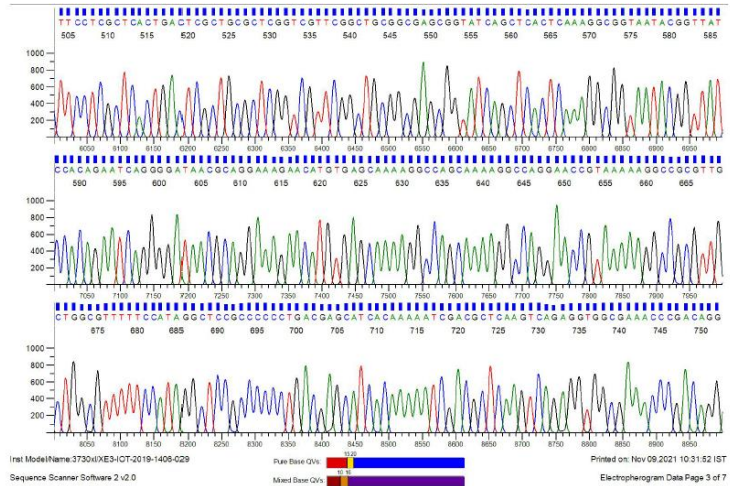

Supplement: S16 Data — (ZIP) [file pgen.1010421.s030.zip › Fig11_E_chromatograms_pdf/1121_074_014_PLD_AP4-1_T7F_F04.pdf]

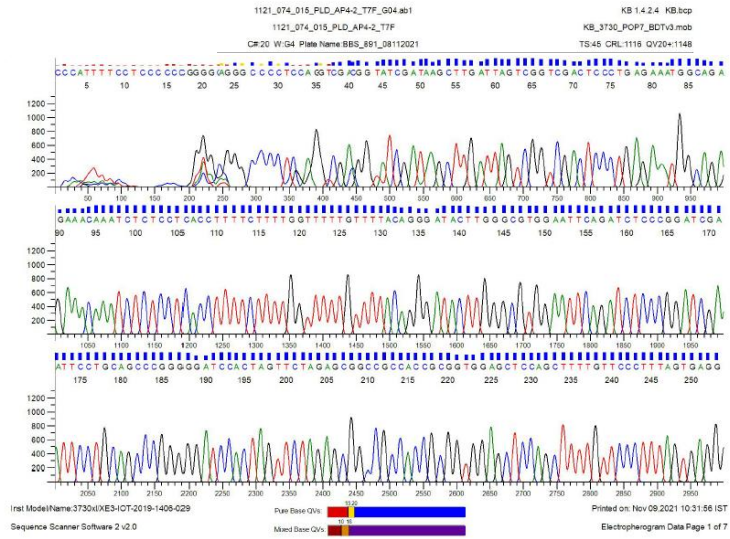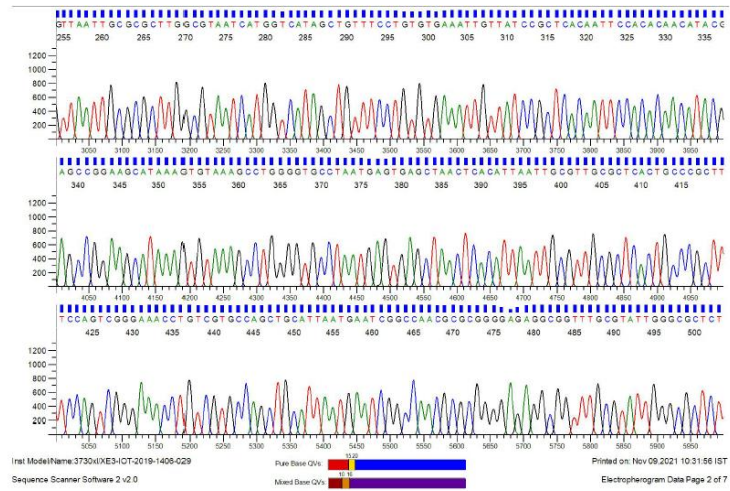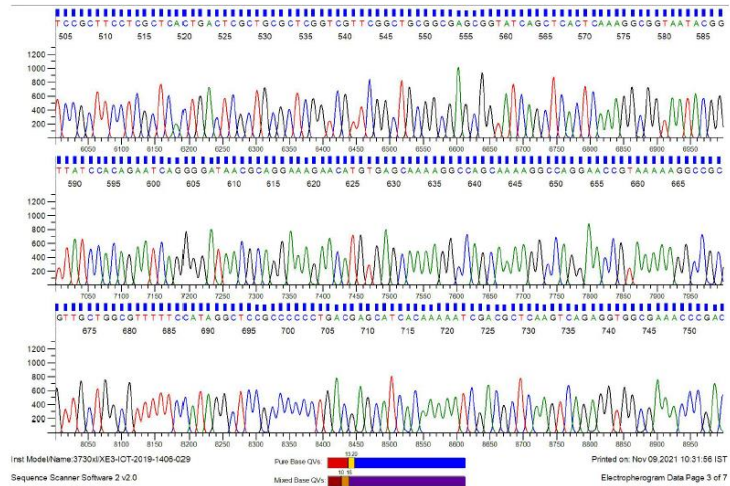

Supplement: S16 Data — (ZIP) [file pgen.1010421.s030.zip › Fig11_E_chromatograms_pdf/1121_074_015_PLD_AP4-2_T7F_G04.pdf]

KB 1.4.2.4 KB.bcp

KB\_3730\_POP7\_BDTv3.mob

TS:55 CRL:853 QV20+:876

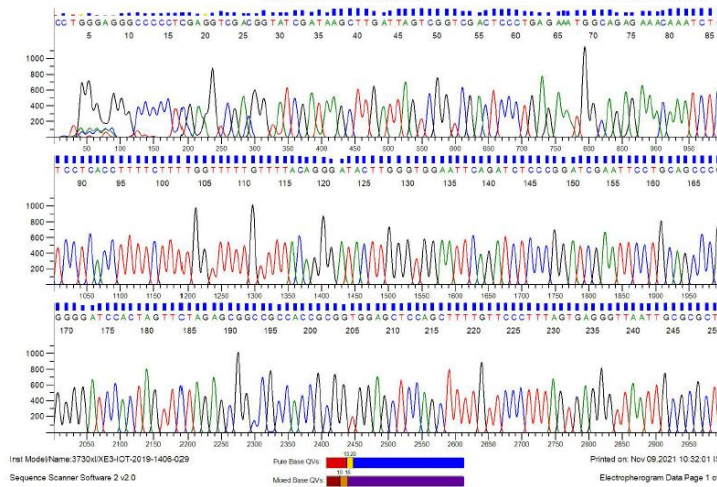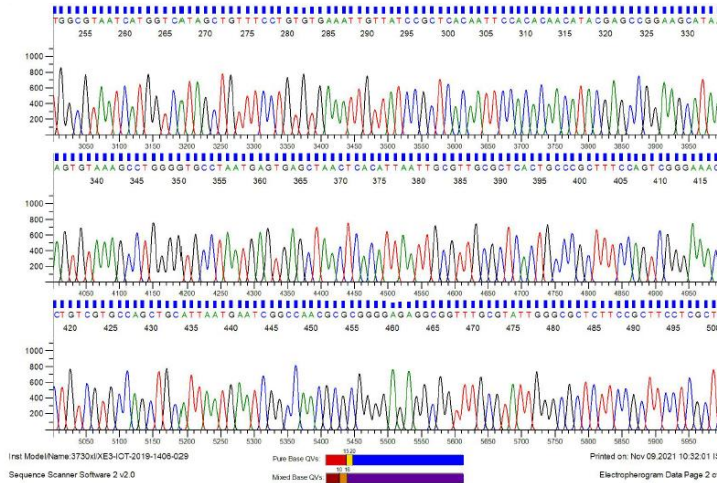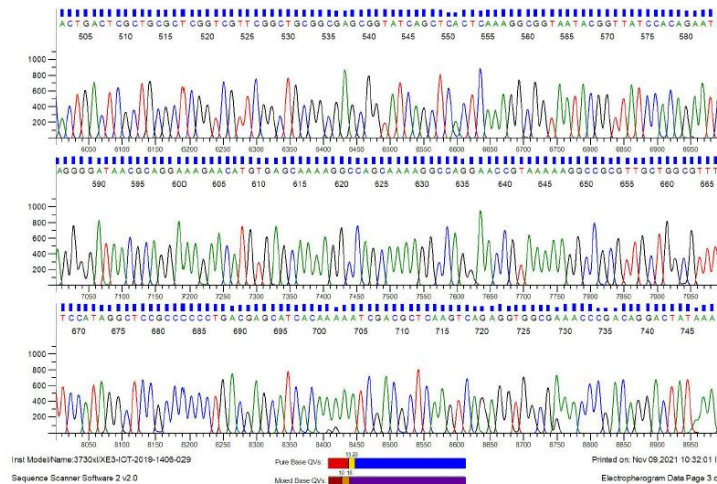

Supplement: S16 Data — (ZIP) [file pgen.1010421.s030.zip › Fig11_E_chromatograms_pdf/1121_074_016_PLD_AP4-11_T7F_H04.pdf]

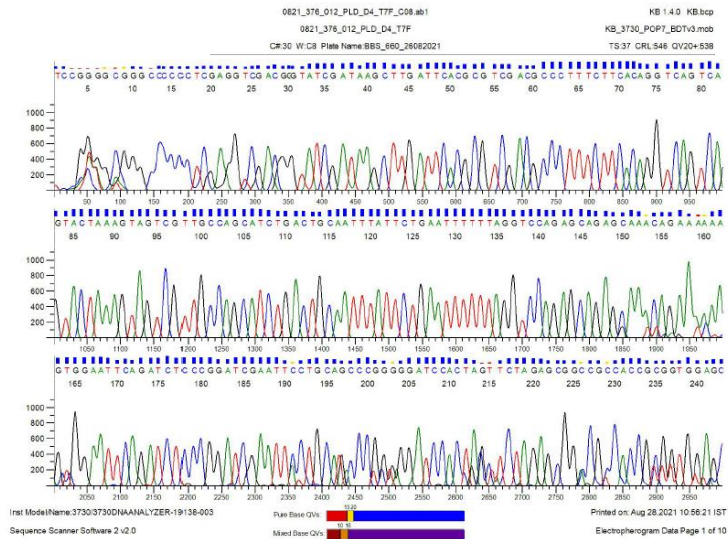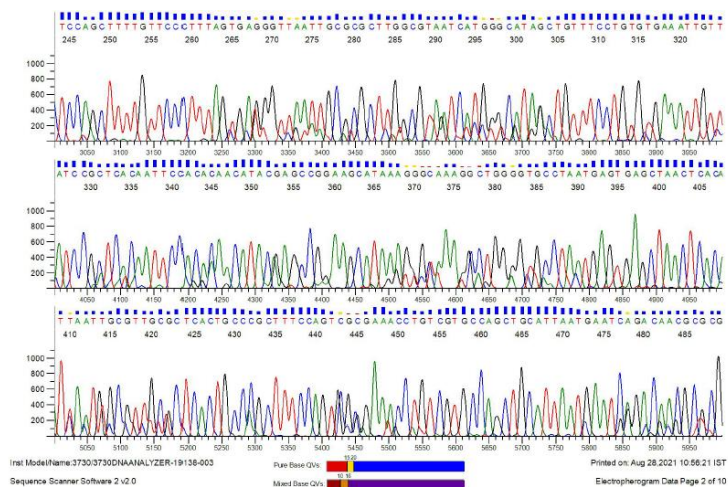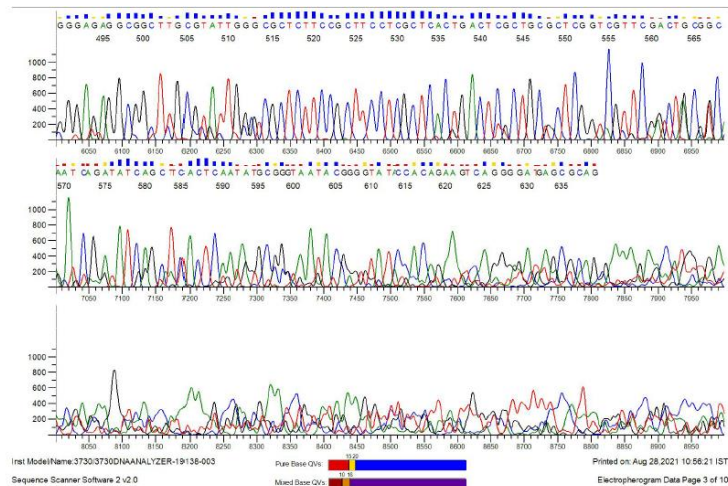

Supplement: S17 Data — (ZIP) [file pgen.1010421.s031.zip › S11_B_chromatograms_pdf/0821_376_012_PLD_D4_T7F_C08.pdf]

KB 1.4.0 KB.bcp

KB\_3730\_POP7\_BDTv3.mob

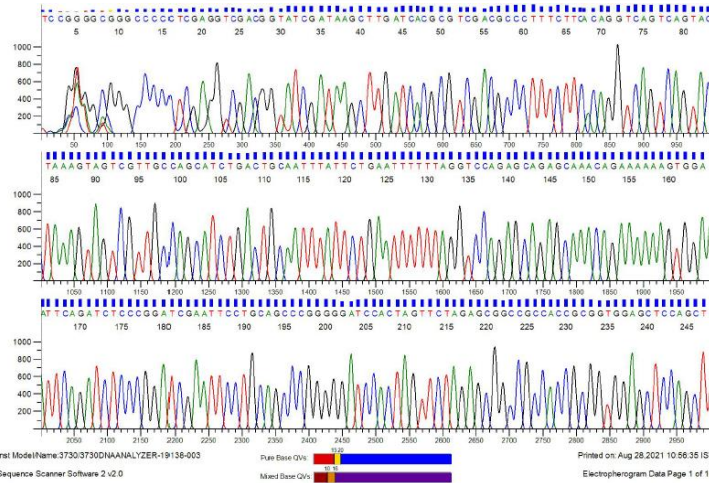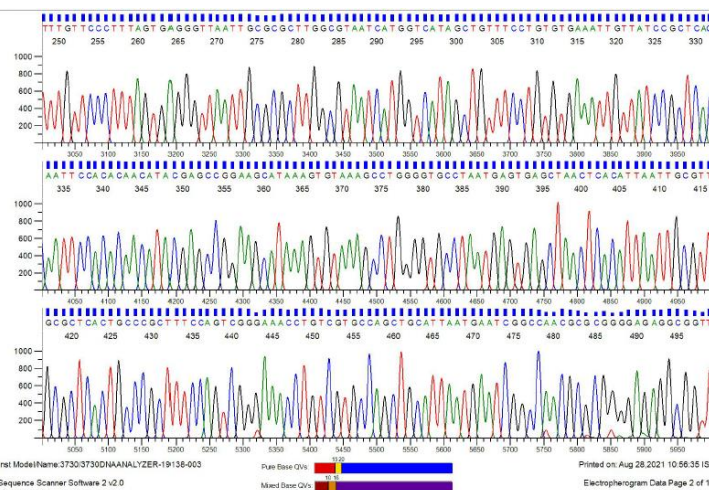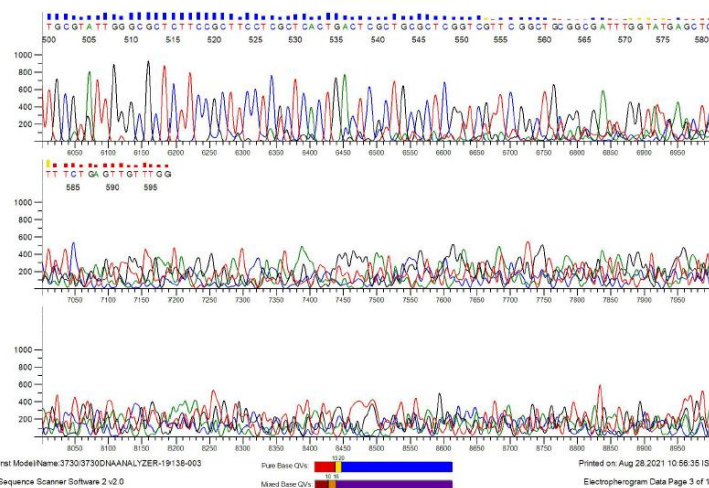

Supplement: S17 Data — (ZIP) [file pgen.1010421.s031.zip › S11_B_chromatograms_pdf/0821_376_014_PLD_D8_T7F_E08.pdf]

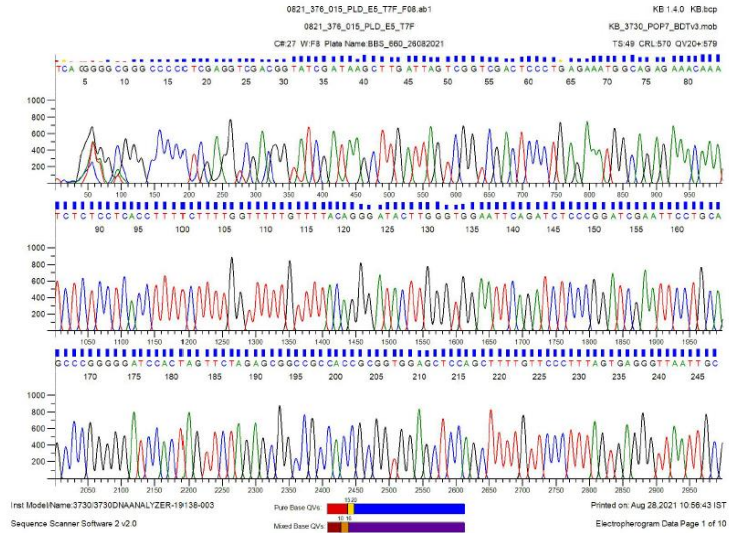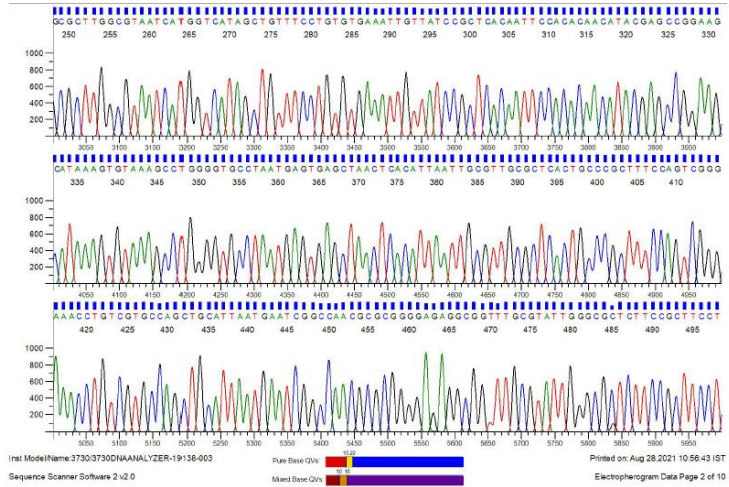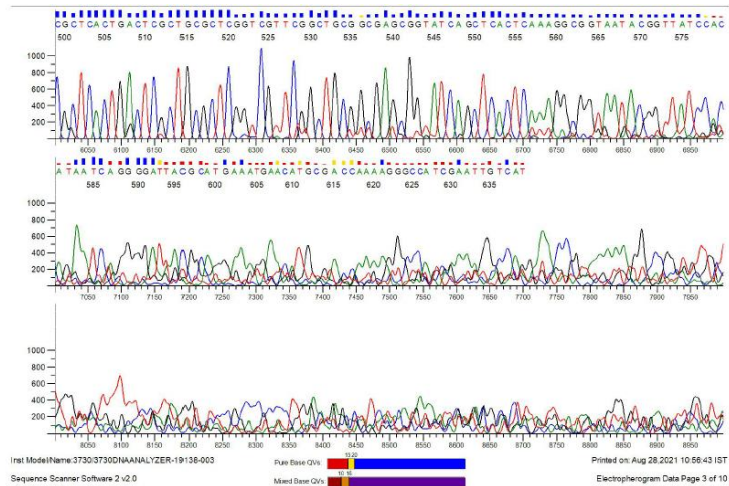

Supplement: S17 Data — (ZIP) [file pgen.1010421.s031.zip › S11_B_chromatograms_pdf/0821_376_015_PLD_E5_T7F_F08.pdf]

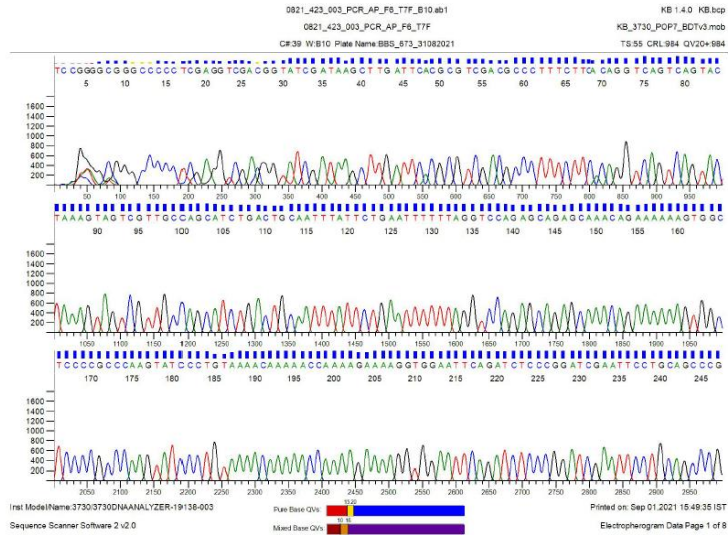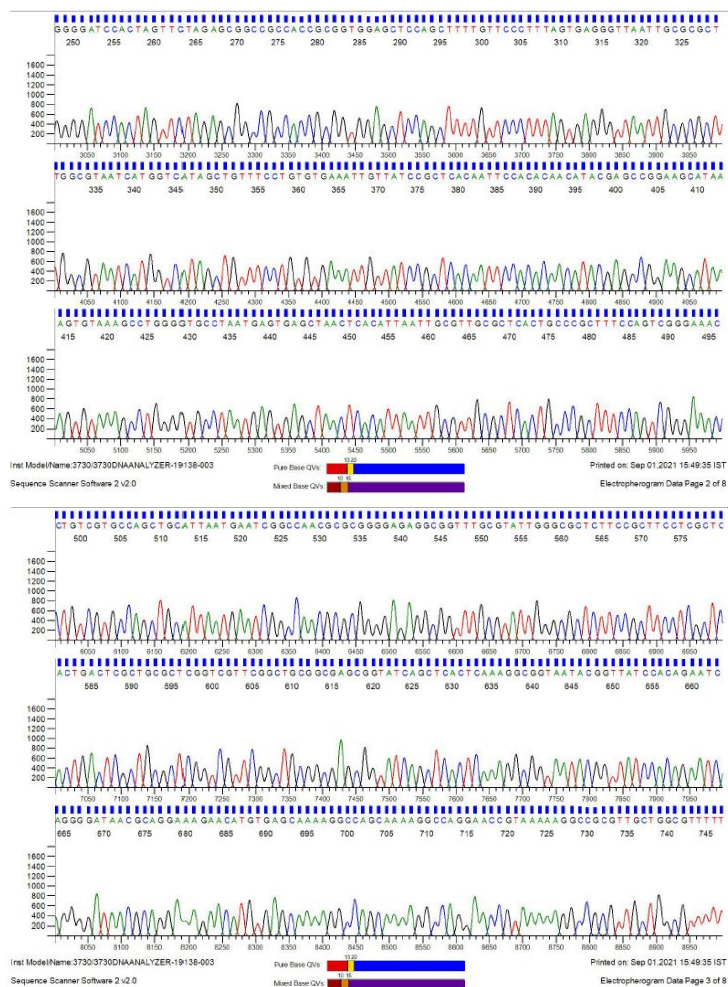

Supplement: S17 Data — (ZIP) [file pgen.1010421.s031.zip › S11_B_chromatograms_pdf/0821_423_003_PCR_AP_F6_T7F_B10.pdf]
