# Supplementary material for: Nonamer dependent RAG cleavage at CpGs can explain mechanism of chromosomal translocations associated to lymphoid cancers
Source: PLoS Genet. 2022 Oct 13;18(10):e1010421. doi: 10.1371/journal.pgen.1010421 (PMC9595545; doi:10.1371/journal.pgen.1010421)
Supplement: S17 Data — (ZIP) [file 0821_376_017_PLD_E7_T7F_H08.pdf › S11_B_chromatograms_pdf/0821_376_017_PLD_E7_T7F_H08.pdf]

0821_376_017_PLD_E7_T7F_H08.ab1 KB 1.4.0 KB.bcp
0821_376_017_PLD_E7_T7F KB_3730_POP7_BDTv3.mob
C#:25 W:H8 Plate Name:BBS_660_26082021 TS:51 CRL:728 QV20+:745

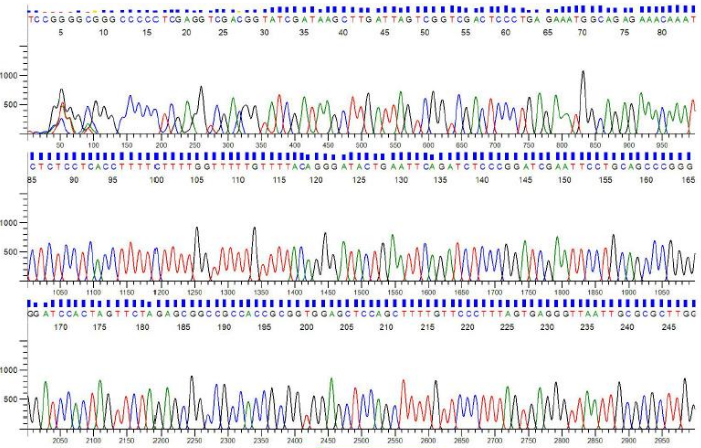

Inst Model/Name:3730/3730DNAANALYZER-19138-003 Pure Base QVs: Printed on: Aug 28,2021 10:56:56 IST
Sequence Scanner Software 2 v2.0 Mixed Base QVs: Electropherogram Data Page 1 of 9

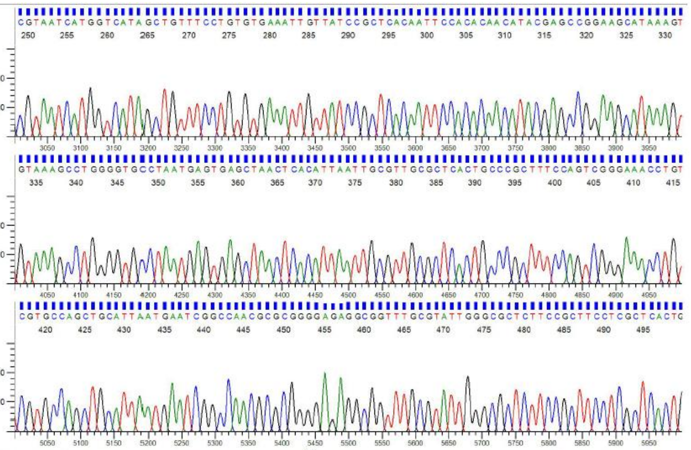

Inst Model/Name:3730/3730DNAANALYZER-19138-003 Pure Base QVs: Printed on: Aug 28,2021 10:56:56 IST
Sequence Scanner Software 2 v2.0 Mixed Base QVs: Electropherogram Data Page 2 of 9

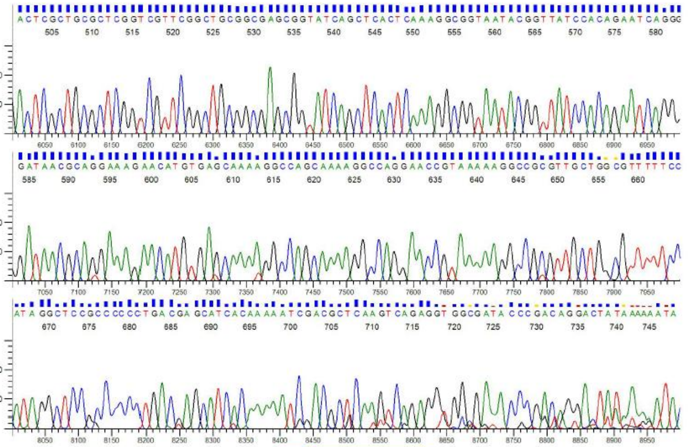

Inst Model/Name:3730/3730DNAANALYZER-19138-003 Pure Base QVs: Printed on: Aug 28,2021 10:56:56 IST
Sequence Scanner Software 2 v2.0 Mixed Base QVs: Electropherogram Data Page 3 of 9
